# Cellular Immune Response Induced by DNA Immunization of Mice with Drug Resistant Integrases of HIV-1 Clade A Offers Partial Protection against Growth and Metastatic Activity of Integrase-Expressing Adenocarcinoma Cells

**DOI:** 10.3390/microorganisms9061219

**Published:** 2021-06-04

**Authors:** Maria Isaguliants, Olga Krotova, Stefan Petkov, Juris Jansons, Ekaterina Bayurova, Dzeina Mezale, Ilze Fridrihsone, Athina Kilpelainen, Philip Podschwadt, Yulia Agapkina, Olga Smirnova, Linda Kostic, Mina Saleem, Oleg Latyshev, Olesja Eliseeva, Anastasia Malkova, Tatiana Gorodnicheva, Britta Wahren, Ilya Gordeychuk, Elizaveta Starodubova, Anastasia Latanova

**Affiliations:** 1 Department of Microbiology, Tumor and Cell Biology, Karolinska Institutet, 17177 Stockholm, Sweden; stefan.petkov@ki.se (S.P.); athina.kilpelainen@stud.ki.se (A.K.); philip.podschwadt@ki.se (P.P.); Linda.Kostic@smi.se (L.K.); Mina.saleem@stud.ki.se (M.S.); britta.wahren@ki.se (B.W.); 2 Department of Research, Riga Stradins University, LV-1007 Riga, Latvia; jansons@biomed.lu.lv (J.J.); Dzeina.Mezale@rsu.lv (D.M.); Ilze.Fridrihsone@rsu.lv (I.F.); 3 N.F. Gamaleya National Research Center for Epidemiology and Microbiology of the Ministry of Health of the Russian Federation, 123098 Moscow, Russia; olga.a.krotova@gmail.com (O.K.); bayurova_eo@chumakovs.su (E.B.); o.smirnova.imb@gmail.com (O.S.); oleglat80@mail.ru (O.L.); olesenka80@mail.ru (O.E.); gordeychuk_iv@chumakovs.su (I.G.); 4 Chumakov Federal Scientific Center for Research and Development of Immune-and-Biological Products of Russian Academy of Sciences, 108819 Moscow, Russia; 5 Engelhardt Institute of Molecular Biology, Russian Academy of Sciences, 119991 Moscow, Russia; estarodubova@yandex.ru; 6 Latvian Biomedical Research and Study Centre, LV-1067 Riga, Latvia; 7 Department of Chemistry and Belozersky Institute of Physicochemical Biology, Moscow State University, 119991 Moscow, Russia; agapkina@belozersky.msu.ru; 8 Institute of Medical Biological Research and Technologies, 143090 Krasnoznamensk, Russia; Malkova@imbiit.com; 9 Evrogen, 117997 Moscow, Russia; tatiana.gorod@evrogen.ru; 10 Institute for Translational Medicine and Biotechnology, Sechenov First Moscow State Medical University, 127994 Moscow, Russia; 11 Center for Precision Genome Editing and Genetic Technologies for Biomedicine, Engelhardt Institute of Molecular Biology, Russian Academy of Sciences, 119991 Moscow, Russia

**Keywords:** HIV-1, ART, therapeutic DNA vaccine, integrase, immunotoxicity, T-cell response, antibodies, murine adenocarcinoma 4T1luc2 cells, lentiviral transduction, tumor growth, metastasis, resistance to tumor growth

## Abstract

Therapeutic DNA-vaccination against drug-resistant HIV-1 may hinder emergence and spread of drug-resistant HIV-1, allowing for longer successful antiretroviral treatment (ART) up-to relief of ART. We designed DNA-vaccines against drug-resistant HIV-1 based on consensus clade A integrase (IN) resistant to raltegravir: IN_in_r1 (L74M/E92Q/V151I/N155H/G163R) or IN_in_r2 (E138K/G140S/Q148K) carrying D64V abrogating IN activity. INs, overexpressed in mammalian cells from synthetic genes, were assessed for stability, route of proteolytic degradation, and ability to induce oxidative stress. Both were found safe in immunotoxicity tests in mice, with no inherent carcinogenicity: their expression did not enhance tumorigenic or metastatic potential of adenocarcinoma 4T1 cells. DNA-immunization of mice with INs induced potent multicytokine T-cell response mainly against aa 209–239, and moderate IgG response cross-recognizing diverse IN variants. DNA-immunization with IN_in_r1 protected 60% of mice from challenge with 4Tlluc2 cells expressing non-mutated IN, while DNA-immunization with IN_in_r2 protected only 20% of mice, although tumor cells expressed IN matching the immunogen. Tumor size inversely correlated with IN-specific IFN-γ/IL-2 T-cell response. IN-expressing tumors displayed compromised metastatic activity restricted to lungs with reduced metastases size. Protective potential of IN immunogens relied on their immunogenicity for CD8+ T-cells, dependent on proteasomal processing and low level of oxidative stress.

## 1. Introduction

Integrase (IN) is a key enzyme in HIV-1 replication cycle; the 3′-processing and strand transfer activities of IN are required for integration of proviral DNA into human genome [1]. As such, it is an important target of antiretroviral therapy (ART). Inhibitors in use block strand transfer activity of IN. The first integrase strand transfer inhibitor (INSTI) raltegravir (RAL) was licensed by FDA in 2007 [2]; the second, elvitegravir (EVG) in 2012 [3], and the third, dolutegravir (DTG) in 2013 [4]. RAL and EVG INSTIs displace the 3′-end of the viral DNA from the active site, and also chelate the divalent cation (Mg^2+^ or Mn^2+^) required for integrase enzymatic activity. Both have been successful in clinical settings, but have relatively low genetic barriers to resistance, resulting in the emergence of HIV isolates with mutations of drug-resistance (DR) to INSTI [5]. Primary resistance substitutions arise in response to INSTI drug pressure, most often through alterations to the enzyme’s active site where the inhibitors bind, causing a decrease in drug susceptibility at the expense of viral fitness [6]. Secondary resistance substitutions arise after continued drug pressure; they alleviate negative effects of the primary mutations and increase the levels of INSTI resistance [7,8]. Of note, RAL and EVG share a high degree of cross-resistance, most of the changes that cause resistance to RAL also cause resistance to EVG, and vice versa [6]. 

We hypothesized that therapeutic vaccination against DR mutations in HIV can induce an immune response against primary mutations of resistance which would act as a “bottleneck” for viral evolution towards the resistant phenotype(s) [9,10,11,12]. Under successful ART, anti-HIV immune response gradually subsides due to extremely low levels of virus replication [9]. Therapeutic vaccination would help to sustain the antiviral immune response aiding in suppression of viral replication [13,14], increasing the efficacy of ART.

Based on these considerations, we proposed to complement functional HIV cure by DNA vaccination against primary DR mutations in the main targets of ART, reverse transcriptase, protease, integrase and gp41. By now, we have developed the prototypes of DNA vaccines against protease (PR) and reverse transcriptase (RT), including their DR forms and demonstrated their immunogenicity in mice [15,16,17]. In DNA-immunized mice, PR behaved as a pure Th1 immunogen inducing cytolytic CD8+ T-cell response dominated by secretion of IFN-γ, and RT, as a Th2 immunogen with immune response dominated by secretion of IL-2 and production of anti-RT antibodies [16,17]. Furthermore, we found that intradermal DNA immunization of mice with DNA-encoding inactivated consensus RT of HIV-1 clade A confers partial protection against growth and metastatic activity of RT-expressing tumor cells, which, in mice, serve as a mimic of HIV-1 challenge [18]. 

HIV-1 integrase is an indispensable component of vaccine against DR HIV-1, feasible to design due to enzyme conservation and high immunogenicity for CD8+ T cells [19,20,21]. We have started with IN with primary mutations of resistance to EVG. EVG blocks replication of HIV, murine leukemia virus (MLV), simian immunodeficiency virus (SIV), and multidrug-resistant strains of HIV-1. A series of clinical trials and cohort studies have identified multiple mutations which lead to treatment failure reducing susceptibility to EVG by >30-fold [22]. This motivates reinforcement of INSTI-based ART with vaccination against EVG-resistant IN. DNA immunogen based on EVG-resistant IN of HIV-1 clade A strain (IN_e3) was designed and tested in mice [23]. In vivo bioluminescence experiments indirectly demonstrated the capacity of CD4+ and CD8+ T-cells specific to EVG-resistant IN to clear cells co-expressing IN and luciferase (Luc) reporter protein [23]. 

This first positive experience had to be expanded to other DR IN variants, first of all, to RAL, the first IN inhibitor approved by U.S. FDA based on the evidence of rapid, potent, and sustained anti-HIV activity in clinical trials. Initially approved for salvage therapy of patients who had developed resistance to first- and second-line ART, RAL was soon promoted to become a first-line drug [22]. It has high bioavailability with minimal interaction with other ART drugs. RAL is highly efficacious with only few differences from the therapy with a second generation INSTI dolutegravir (DTG) [5]. These considerations motivate continued use of this first generation IN inhibitor despite its low genetic barrier to resistance [24]. For RAL, as for EVG, a low genetic barrier to resistance can be counteracted by immune response against primary mutations of resistance to RAL, preventing or hindering their emergence. 

Meeting this challenge, we designed and tested two prototype DNA vaccines against RAL-resistant variants of IN, complementing EVG-resistant IN DNA vaccine variant. Properties of DR IN variants, specifically their proteolytic stability, degradation by proteasome, lysosome, and capacity to induce oxidative stress were assessed and related to the performance of these genes in DNA immunization of mice. Previously, we could not directly assess the protective potential of IN-specific immune response, namely its capacity to kill IN-expressing cells, due to the absence of HIV-1 challenge system for large scale immunization/screening experiments in mice at a low biosafety risk level (outside of BSL 2 settings). Meeting this challenge, we created clones of murine mammary gland adenocarcinoma cells expressing IN variants to use them as a mimic of challenge with HIV-1-infected cells. Here, we demonstrate that DNA immunization with RAL resistant variants of IN can induce potent cellular immune response that partially protects mice against growth and metastatic activity of tumor cells expressing HIV-1 IN variants. 

## 2. Materials and Methods

### 2.1. Synthetic Integrase Genes/Cloning of Integrase Genes to Vectors

Full-length HIV-1 clade A integrase sequences from treatment-naïve patients followed up in the territory of the former Soviet Union (*n* = 34; Belarus, Estonia, Georgia, Russia, Ukraine, and Uzbekistan) were selected [23,25]. Integrase consensus was created using BioEdit software (Hall T.A., BioEdit 7.2.5.; BioEdit Sequence Alignment Editor; Ibis Biosciences, Carlsbad, CA, USA, 2013). A humanized synthetic gene encoding the respective amino acid sequence (IN_a) was designed using the web service utility at http://genomes.urv.es/OPTIMIZER (accessed on 12 April 2015) [26]. The design of humanized genes included modeling of RNA folding done using the web services at http://www.invitrogen.com (accessed on 12 April 2015), http://www.unafold.org/RNA_form.php (accessed on 12 April 2015) and http://genomes.urv.es/OPTIMIZER/ (accessed on 12 April 2015). Met-Gly dipeptide was added to the N-terminal Phe residue of IN. Together with the insertion of an ATT triplet upstream of the AUG codon, this introduced the consensus Kozak’s sequence ANNATGG required for the efficient initiation of IN gene translation [27]. The resulting mRNA was checked for the absence of undesirable folding (UNAFold at http://www.unafold.org/RNA_form.php (accessed on 12 April 2015), and OPTIMIZER at http://genomes.urv.es/OPTIMIZER/ (accessed on 12 April 2015). Synthetic DNA encoding IN_a (Evrogen, Moscow, Russia) was flanked with extra 5′- and 3′-terminal sequences: GGATCC prior to the ATT-ATG-GGC sequence at the 5′-terminus introducing *BamHI*, and GAATTC following TGA stop-codon at the 3′-terminus introducing *EcoRI* restriction sites, and cloned into pVax1, cleaved by *BamHI/EcoRI*, to generate a vector for eukaryotic IN expression pVaxIN_a. Generation of plasmid pVaxIN_in encoding inactivated parental IN_a (IN_in) was described earlier [23]. DNA fragment encoding IN_a was PCR-amplified and ligated into the *NdeI/BamHI*-cleaved plasmid pET15b in frame with the codons for the N-terminal 6His-tag to generate vector pETIN_a for prokaryotic IN expression [25]. Raltegravir-resistant IN_a variants with two patterns of primary resistance to RAL: L74M, E92Q, V151I, N155H, G163R (IN_a_r1) and E138K, G140S, Q148K (IN_a_r2), and their variants inactivated by mutation D64V (IN_in_r1 and IN_in_r2) were generated by site-directed mutagenesis of pETIN_a using the QuikChange II Site-Directed Mutagenesis kit (Agilent Technologies, Santa Clara, CA, USA) with generation of plasmids pETIN_a_r1, pETIN_a_r2, pETIN_in_r1, and pETIN_in_r2, respectively. IN_a_r1, IN_a_r2, IN_in_r1, and IN_in_r2 coding sequences were recloned into a pVax1 vector (Invitrogen, Waltham, MA, USA) using *BamHI* and *EcoRI* restriction sites, generating plasmids pVaxIN_a_r1, pVaxIN_a_r2, pVaxIN_in_r1, and pVaxIN_in_r2, respectively. 

IN-expressing lentiviral vectors were obtained as described previously [28]. In brief, coding sequences for IN variants were recloned from pVax-based vectors into lentiviral vector pRRLSIN.cPPT.PGK (Addgene; https://www.addgene.org/12252/ (accessed on 10 December 2016)) under the control of 3-phosphoglycerate kinase (PGK) promoter, generating respective IN-encoding plasmids. Plasmids were purified using endotoxin-free DNA purification kits (Qiagen, Germantown, MD, USA) and sequenced (Eurofins MWG Operon, Ebersberg, Germany). 

### 2.2. Recombinant Proteins

IN proteins used in the in vitro tests of enzymatic activity, in cell culture studies and tests of IN-specific immune response were expressed from respective pET15b-based prokaryotic expression vectors in *E. coli* BL21(DE3) strain carrying pRARE plasmid from Rosetta (DE3) strain (Novagen^®^, Merck Millipore, Darmstadt, Germany, and Billerica, MA, USA). Competent *E. coli* cells were transformed with pETIN_a, or pETIN_in, or pETIN_a_r1, or pETIN_a_r2, or pETIN_in_r1, or pETIN_in_r2. IN expression was induced by adding IPTG. The recombinant IN variants were purified as described previously [29]. 

### 2.3. Synthetic Peptides

Amino acid sequences of IN variants were aligned with the sequences of known B- and T-cell epitopes of integrase of HIV-1 clades A, B, and C recognized in different species [19,30,31,32,33,34,35,36,37,38,39,40,41,42,43,44,45,46,47,48,49,50,51,52,53], including the epitope data from Los Alamos HIV (www.hiv.lanl.gov) (accessed on 07 March 2013) and IEDB databases (http://www.immuneepitope.org) (accessed on 10 March 2013). Regions homologous to the known epitopes were selected. IN was also assessed for peptides with strong binding to HLA-A*2 (http://tools.immuneepitope.org/analyze/html/mhc_binding.html) (accessed on 10 March 2013) (Supplementary Figure S1). Respective regions were represented by synthetic peptides (GL Biochem Ltd., Shanghai, China) (Supplementary Table S1). Peptides were used in Fluorospot tests and multiparametric FACS to assess cellular responses against IN. Peptide Luc (GFQSMYTFV; GL Biochem Ltd., Shanghai, China), representing a CTL epitope of firefly luciferase restricted to H2-Kd in mice, was used as a control [54]. 

### 2.4. Assays of Integrase Enzymatic Activities 

**DNA duplexes for assessing integrase activity.** Integrase activities were assessed using synthetic DNA duplexes (Supplementary Table S2). DNA duplex U5 consisting of the oligonucleotides U5B and U5A, which mimicked the end of HIV-1 U5 LTR, served as a substrate for 3′-processing activity. Duplex U5-2, formed by U5B-2 and U5A, was used as a substrate for strand transfer and duplex Ran formed by oligonucleotides RanB and RanA, to verify the specificity of 3′-processing. To measure integrase catalytic activities, the oligonucleotides U5B, U5B-2, and RanB (10 pmol each) were labeled using T4 polynucleotide kinase and 50 μCi of [γ-^32^P]ATP (3000 Ci/mmol). After 1 h of incubation at 37 °C, EDTA was added to the final concentration of 50 mM, and the reaction mixture was heated for 5 min at 65 °C to inactivate the kinase. Labeled oligonucleotides were supplemented with equimolar amounts of unlabeled complementary oligonucleotides and annealed by first heating for 3 min at 90 °C and then cooling slowly to room temperature. Resulting duplexes were purified using Micro Bio-Spin columns P-6 (Bio-Rad, Berkeley, CA, USA).

**3′-end processing and strand transfer reactions.** All assays were carried out as described previously [55]. In brief, DNA duplexes (10 nM) were incubated for 2 h with 100 nM IN protein in 20 μL of the buffer containing 20 mM HEPES, pH 7.2, 7.5 mM MgCl_2_, and 1 mM DTT, at 37 °C. DNA fragments were precipitated with ethanol and separated in denaturing 20% polyacrylamide gels. Gels were analyzed on a Storm 840TM Phosphorimager (Molecular Dynamics, Sunnyvale, CA, USA) and quantified with Image-QuantTM 4.1 software (Amersham Biosciences Corp., Piscataway, NJ, USA). Integrase activity was defined as the percent of a substrate converted to a product; activities of IN variants were quantified in relation to values exhibited by the parental IN_a variant. Each experiment was repeated at least three times. 

### 2.5. Transient Expression of Integrases in Eukaryotic Cells

Eukaryotic expression of IN directed by pVax1-based vectors was assessed as described previously [23]. In brief, cultured HEK293T, HeLa and NIH3T3 cells (ATCC, Manassas, VA, USA) were transfected with pVaxIN_a, pVaxIN_a_r1, pVaxIN_a_r2, pVaxIN_in, pVaxIN_in_r1, pVaxIN_in_r2, or empty vector pVax1 using Lipofectamine LTX (Invitrogen Corporation, Carlsbad, CA, USA); 48 h post-transfection, their lysates were analyzed in 12% SDS-PAAG with subsequent Western blotting using monoclonal mouse antibodies against IN (anti-IN Mab IN-2 #ANT0071, kindly provided by Dr. Elisa Gargiullo, Diatheva, Fano, PU, Italy). To normalize for the total protein content, membranes were re-stained with monoclonal mouse anti-actin antibody (Sigma-Aldrich, St. Louis, MO, USA). Films were scanned and the relative intensity of the bands was estimated using ImageJ software (US National Institutes of Health, Bethesda, MD, USA). To assess the level of IN expression per cell, the percent of cells expressing IN was estimated from the efficacy of transfection established in a control co-transfection with a reporter GFP plasmid; % transfection gave the number of cells expressing IN among 5000 cells resolved by PAGE and Western blotting in one PAGE well. Samples of recombinant IN in a range from 0.1 to 10 ng were resolved on the same gel as calibration controls. IN protein content in a lysate was quantified by plotting the intensity of the respective IN band on the film (grey units; Image J) against the IN calibration curve; IN content per cell was calculated by dividing this value by the number of expressing cells.

### 2.6. Generation of IN Expressing Lentiviral Particles

To obtain IN-encoding lentiviral particles, plasmids pLVIN_a, pLVIN_a_r1, and pLVIN_a_r2 and plasmids encoding glycoprotein pMD.G and structural lentiviral proteins deltaR8.91 were used to co-transfect HEK293T cells [56]. Lentiviral particles were concentrated 10-fold with Amicon Ultra-15 100 K centrifuge concentrators (Merck-Millipore, Darmstadt, Germany). Infectious titers of the lentiviral particles were determined in HT1080 cells by quantitative real-time PCR with primers and probes indicated in Supplementary Table S3 [56] using standard samples of HT-1080 DNA with a known number of viral genome copies. Use of pLVIN_a and pLVIN_a_r2 allowed us to generate viable lentiviral particles encoding IN_a and IN_a_r2. No viable lentivirus particles were generated using pLVIN_a_r1.

### 2.7. Lentiviral Transduction of 4T1luc2 Cells and Isolation of Clones Expressing IN Variants 

Lentivirus particles encoding IN_a and IN_a_r2 were used to transduce murine mammary gland adenocarcinoma cells expressing firefly luciferase 4T1luc2 (Bioware Ultra Cell Line 4T1luc2, caliperls.com/assets/014/7158.pdf (accessed on 20 February 2015); Caliper Life Sciences Inc., Hopkinton, MA, USA), as was described previously [28]. Transduction was performed at the multiplicity of infection (MOI) of 1, 5, or 20 of transducing units per cell. Attempts to transduce with MOI >1 for IN_a and MOI > 5 for IN_a_r2 expressing variants failed due to massive cell death (Supplementary Figure S3) which was attributed to the toxicity of IN variants to expressing cells. Transduced cells lines were cloned to single cells by limiting dilution in 96-well plates resulting in IN_a expressing clone 4T1luc2_IN_a_1.2 and IN_a_r2 expressing clone 4T1luc2_IN_a_r2_1.5. Clones were cultured in full RPMI-1640 medium with 10% FBS and 100 mg/mL penicillin/streptomycin mix at 37 °C in an 5% CO_2_ and split every 2–3 days. Genomic DNA isolated from the cells using DNeasy Blood & Tissue Kit (Qiagen, Germantown, MD, USA) was analyzed for the presence of genomic inserts of IN DNA by PCR with a pair of primers specific for the lentiviral vector backbone flanking the inserts (Supplementary Table S3), which confirmed the presence of IN_a and IN_a_r2 inserts in 4T1luc2_IN_a_1.2 and 4T1luc2_IN_a_r2_1.5 clones, respectively (Supplementary Figure S4). 

### 2.8. Proteolytic Degradation of Integrase Variants in Eukaryotic Cells

**Proteasome and lysosome inhibition assays**. HeLa cells were transfected with pVaxIN_a, pVaxIN_a_r1, pVaxIN_a_r2, pVaxIN_in, pVaxIN_in_r1, pVaxIN_in_r2 plasmids, or pVax1 vector. Inhibitors were added to the medium 30 h post-transfection, and cells were incubated for additional 18 h. Degradation of IN variants by proteasome was probed with MG132 (10 μM) or epoxomicin (0.5 μM), and by lysosome, with chloroquine (10 μM) as a general inhibitor of lysosome proteolysis, and with inhibitors of individual classes of lysosomal proteases leupeptin (10 μg/mL), aprotinin (100 μg/mL), pepstatin (5 μg/mL), or E-64 (10 μМ) (all from Calbiochem, San Diego, CA, USA). After incubation, cells were lysed and analyzed by SDS-PAGE with subsequent Western blotting with anti-IN monoclonal antibodies. Western blots were quantified using ImageJ.

**Pulse-chase/cycloheximide-chase.** For cycloheximide-chase assay, HeLa cells were transfected with pVaxIN_a, pVaxIN_a_r1, pVaxIN_a_r2, pVaxIN_in, pVaxIN_in_r1, pVaxIN_in_r2 plasmids, or pVax1 vector, and 48 h post-transfection treated with cycloheximide (Sigma-Aldrich, St. Louis, MO, USA) at the final concentration of 100 μg/mL. Cells, harvested prior to and 2, 4, 6 h after the addition of cycloheximide, were lysed and analyzed by SDS-PAGE with subsequent Western blotting with anti-IN monoclonal antibodies, quantified using ImageJ.

### 2.9. Measurement of the Reactive Oxygen Species 

Measurement of reactive oxygen species (ROS) was performed as described earlier [57]. In brief, 40 h post-transfection, HEK293T cells were incubated for 30 min in cell culture medium containing 25 μM 2′,7′-dichlorodihydrofluorescein diacetate (DCFH-DA). Fluorescence intensities were measured using Plate CHAMELEON V reader (Hidex Ltd., Turku, Finland) with the excitation at 485 nm and emission at 535 nm.

### 2.10. Animal Experiments, Ethics Statement

Experiments in laboratory mice were carried in compliance with the bioethical principles adopted by the European Convention for the Protection of Vertebrate Animals Used for Experimental and Other Scientific Purposes (Strasbourg, 1986). Experimental procedures were approved by the Northern Stockholm’s Unit of the Ethics of Animal Research on 2010-08-26, ethical permission N197/10 “Evaluation of the new generation of vaccines against highly dangerous infectious diseases and cancer” and ethical committee of the Gamaleya Research Center for Epidemiology and Microbiology, Moscow, Russia (protocol N10, 14 March 2017). Tumor implantation experiments were done in 8 week old BALB/c mice purchased from “Pushchino” breeding facility of the Institute of Bioorganic Chemistry RAS (Pushchino, Russia). Testing of IN toxicity was performed in BALB/c mice received from the Scientific Center of Biomedical Technologies of the Federal Medical and Biological Agency of Russia (Andreevka, Russia). Mice were housed at the animal facility of Gamaleya Research Center of Epidemiology and Microbiology (Moscow). For DNA immunization experiments including those with tumor challenge, BALB/c (H2-Dd) mice (females, 8 weeks old) were purchased from Charles River Laboratories (Sandhofer, Germany) and housed at the Astrid Fagrius Laboratory (Karolinska Institute, Stockholm, Sweden). In all experiments, mice were housed in the environment-enriched cages, 5–8 animals per cage under a 12 h/12 h light-dark cycle with ad libitum access to water and food. Animals were regularly controlled for the food and water intake, weight development, skin and fur changes and microscopic alterations at the site of immunization. All procedures were evaluated as having low to average degree of difficulty. Possible mouse discomfort under immunization, monitoring, and sample collection was relieved by the inhalation anesthesia. Animals were sacrificed by cervical dislocation.

### 2.11. Implantation of 4T1luc2 Clones and Follow-up of Tumor Growth 

The capacity of subclones 4T1luc2_IN_a_1.2 and 4T1luc2_IN_a_r2_1.5 to form tumors and metastases was tested by their ectopic implantation into 8-week-old female BALB/c mice. Prior to injection, 4T1luc2_IN_a_1.2 and 4T1luc2_IN_a_r2_1.5 and parental 4T1luc2 cells grown in the selective medium were detached, sedimented, washed with serum-free RPMI-1640, stained for viability with Trypan Blue dye (Life Technologies, Carlsbad, CA, USA), and counted in a hemocytometer. Aliquots of 10^4^ cells were injected subcutaneously into the right and left flanks of a mouse using a 25G needle mounted on an insulin syringe (B Braun, Melsungen, Germany). Implantation success by day 1 and further tumor growth were assessed by bioluminescent imaging (BLI) as described earlier [23]. BLI was performed on days 2–4, and then every 2–3 days until the first mouse in the experiment reached the clinical observation-based endpoint (https://oacu.oir.nih.gov/sites/default/files/uploads/arac-guidelines/b13_endpoints_guidelines.pdf) (accessed on 02 September 2014). Mice were weighed at each monitoring point. For this, mice were intraperitoneally injected with PBS-solved XenoLight D-luciferin potassium salt (PerkinElmer, Waltham, MA, USA) (150 mg/kg based on the actual weight), left for 5 min, anesthetized for 5 min with 2–2.5% isoflurane/air delivered in the inhalation chamber or via nasal masks (Baxter Medical AB, Kista, Sweden), and monitored for photonic emissions in the in vivo imager Spectrum CT (PerkinElmer, Waltham, MA, USA) using Living Image software (Living Image®, version 4.5, PerkinElmer, Waltham, MA, USA) Bioluminescence from each implantation site was presented as total flux (photon/sec) per site of injection. Tumor size was also assessed by morphometric measurements done at regular intervals using calipers; tumor volume was calculated using the standard formula for xenograft volume: *V* = *xy*^2^/2 [58,59].

At the experimental endpoint mice were humanely euthanized, the tumors and organs, such as spleen, liver, and lungs, known to be affected by distant metastases in 4T1 tumor model [60,61], were dissected with surgical scissors. Tumors and organs were transferred into the wells of a 24-well tissue culture test plate (Wallac, Turku, Finland) containing 2 mL RPMI-1640 medium. Ex vivo bioluminescent imaging of organs was performed as described for in vivo BLI. Number of Luc-expressing cells was calculated using in vitro calibration curves built with 10^2^ to 10^5^ 4T1luc2 cells [62]. Thereafter, tumors and all organs, except for spleens, were transferred to 5 mL of 4% formaldehyde solution in PBS, incubated from 24 to 48 h at +6 °C, and then washed five times with PBS and used to prepare FFPE blocks to be analyzed by histological methods as described below. Spleens were washed from luciferin with PBS, and used to isolate splenocytes (see below). Parts of tumors were also frozen in liquid nitrogen at −80 °C to be analyzed for mRNA expression of IN. All the experiments were done in two independent runs.

### 2.12. Expression of IN mRNA by 4T1luc2 Subclones

Total RNA was isolated from tumors frozen in liquid nitrogen using ExtractRNA reagent (Evrogen, Moscow, Russia). Residual genomic DNA in the samples was removed by treatment with DNAse I (NEB) for 30 min at 37 °C. RNA was isolated and cleaned with CleanRNA Standard kit (Evrogen, Moscow, Russia). The efficiency of DNAse I treatment was controlled by Real-Time PCR. Transcription of IN genes was assessed by RT-PCR using OneTube SYBR-RT PCR kit (Evrogen, Moscow, Russia) using primers specific to IN and to murine GAPDH (Supplementary Table S3). The RT-PCR reaction was performed on BioRad PCR-machine at 45 °C for 15 min, 95 °C 1 min followed by 40× [95 °C 15 s, 62 °C 20 s] cycles. 

### 2.13. Tumor Histology and Ex Vivo Assessment of the Metastases 

FFPE blocks were prepared from the formalin-fixed tumor tissues and murine lungs, sectioned on microtome according to the standard protocols [63] (https://www.protocolsonline.com/histology/sample-preparation/paraffin-processing-of-tissue/) (accessed on 05 August 2016). Sections mounted on slides were dewaxed, rehydrated, stained with Mayer’s hematoxylin solution, then washed, rinsed, and counterstained with eosin Y solution, after that, dehydrated, washed with absolute alcohol and covered with cover slips for microscopic evaluation. Histological evaluation was based on the standard parameters, such as acinar formation, nuclear size, and pleomorphism and mitotic activity [63]. Grade of the tumors was calculated according to Nottingham system [64]. The slides were examined by light microscopy (Leica DM500, Wetzlar, Germany). Formalin-fixed, Paraplast-embedded lung tissues were used to diagnose and evaluate the formation of metastases. For each mouse, the area of tumor metastases was quantified in 25 high-power (×400) microscope fields of hematoxylin-eosin-stained slides by computer-assisted morphometry using specialized NIS Elements software (Nikon, Tokyo, Japan). 

Frozen tumor samples were finely dispersed under aseptic conditions, and genomic DNA was isolated using the DNA Extract reagent kit (Evrogen, Moscow, Russia) according to the protocol recommended for cell cultures. The presence of sequences encoding IN_a variants was confirmed by PCR using a pair of primers specific to lentiviral vector (PGKseq and LVT-200R and a pair of primers to the actin gene as a control (Supplementary Table S3). PCR reaction was performed on the BioRad PCR machine at 95 °C for 5 min followed by 30 cycles (95 °C 15 s, 65 °C 30 s, 72 °C 90 s), and 72 °C 5 min. 

### 2.14. DNA Immunization of Mice with IN Variants

Four series of DNA immunization experiments were performed; the first two were intended to evaluate the immunogenicity of IN-encoding plasmids, the third, to study toxicity of these plasmids, and the fourth, to estimate the potential of IN-specific immune response to eliminate IN-expressing tumor cells (Table 1). In the first series, groups of female 8 week old BALB/c (H2-Dd) mice (*n* = 4) were immunized with 2 × 10 µg of DNA immunogen (pVaxIN_a, pVaxIN_in, pVaxIN_in_r1, pVaxIN_in_r2) or empty pVax1 (Table 1, Series I) mixed with an equal amount of reporter pVaxLuc plasmid. In the second, immunization with 2 × 10 µg of pVaxIN_in_r1 and pVaxIN_in_r2 mixed with an equal amount of pVaxLuc (*n* = 6) was repeated to obtain additional splenocytes to further type T-cell response using FACS (Table 1, Series II). In both series, DNA was delivered as two intradermal injections with a 29G insulin-grade syringe (Micro-Fine U-100; BD Consumer Healthcare, Franklin Lakes, NJ, USA) in the lower back to the left and to the right from the base of the tail. Immediately after, a needle array electrode (1.5 × 4 mm gaps; Cellectis, Romainville, France) was placed over the injection site and voltage was applied using DermaVax electroporator (Cellectis) in a regimen optimal for small rodents [65]. On days 4, 9, 15, and 21 after the injection, mice were subjected to in vivo imaging of the luciferase expression as described earlier [23]. At day 15, the mice were bled, and, at day 22, they were bled and sacrificed, and spleens were collected. Prior to intradermal injection, electroporation, and bleeding, the mice were anesthetized with 2–2.5% isoflurane/air. 

In the third and fourth series, immunization was performed using DNA immunization regimen optimized in our previous studies [16] (Table 1, Series III and IV). In brief, plasmids were delivered as intradermal injections with a 29G insulin-grade syringe under the left shoulder blade and on the lower back to the left and to the right from the base of the tail (three injections; Series III) or only the latter two sites (two injections; Series IV). Immunizations were followed by in vivo electroporation (CUY21EditII, BEX, Tokyo, Japan) with fork-plate electrode (CUY663-5_10; BEX) with a poration pulse of 400V (0.1 ms with a 20 ms interval) followed by 8 altering polarity (+/−) driving pulses of 10 ms performed at 100V with 20 ms intervals (10 ms 100V pulses with opposed polarity) [16]. Three (Series III) or four (Series IV) weeks later mice received plasmid boosts with the same regimen. In Series IV, boost was followed by challenge with tumorigenic cells expressing IN variants (Table 1 and text below). 

### 2.15. Isolation of Murine Splenocytes 

Murine spleens collected at experimental endpoints were homogenized. Single-cell suspensions were treated with Red Blood Cell lysing buffer and re-suspended in RPMI supplemented with 2 mM L-glutamine, 2 mM Penicillin-Streptomycin (all from Sigma-Aldrich, St. Louis, MO, USA), and 10% FBS (Gibco, Invitrogen, Carlsbad, CA, USA) (complete media) to obtain splenocytes.

### 2.16. IFN-γ ELISpot and IFN-γ/IL-2 Fluorospot 

IN-specific reactivity of murine splenocytes was assessed by IFN-γ ELISpot and IFN-γ/IL-2 Fluorospot. Cells were incubated in RPMI medium supplemented with 2 mM L-glutamine, 2 mM penicillin-streptomycin, and 10% FBS (Gibco, Invitrogen, Carlsbad, CA, USA) (complete media). Splenocytes were stimulated at 37 °C and 5% CO_2_ with IN peptides (10 μg/mL) (Supplementary Table S1) or recombinant IN_HXB2 protein at 6 and 12 μg/mL, or luciferase-derived peptide GFQSMYTFV (LucP) at 10 μg/mL, or mitogen control Concanavalin A (ConA) at 5 μg/mL. All stimuli were diluted in RPMI 1640 supplemented with 5% FBS, 100 U/mL penicillin, 100 μg/mL streptomycin, and 0.3 mg/mL glutamine (all from Gibco, Life Technologies Co., Carlsbad, CA, USA); cell culture medium served as a negative control. After 20 h incubation, IFN-γ and IL-2 secretion by splenocytes was assessed in IFN-γ ELISpot or dual IFN-γ/IL-2 Fluorospot tests (Mabtech, Nacka, Sweden) in accordance with the protocols provided by the manufacturer. The number of cytokine-producing spot-forming units (sfu) per million was evaluated using AID ELISpot or AID iSpot FluoroSpot Reader Systems (Autoimmun Diagnostika GmbH, Strassberg, Germany). A net SFC/10^6^ cells in response to stimulation in individual animals was calculated by subtraction of the background response registered in cell culture medium. The response to specific antigens was considered specific if it exceeded the mean net response to these antigens in the empty vector-immunized mice + 3SD. 

### 2.17. Flow Cytometry with Intracellular Cytokine Staining 

All reagents used in flow cytometry with intracellular cytokine staining (ICCS) were from BD Biosciences (Franklin Lakes, NJ, USA) if not mentioned otherwise. Splenocytes were incubated for 4 h in one time-point assessment (immunization Series I, Table 1) or for 18 h and 90 h for kinetics assessment (immunization Series II, Table 1). Incubations were done with an equimolar mixture of peptides representing mouse CD4^+^ and CD8^+^ epitopes (MIN pool; Supplementary Table S1), or a mixture of recombinant IN_a_r1 and IN_a_r2 proteins (5 μg/mL each), or positive controls ConA at 2 µg/mL, or phorbol myristate acetate (PMA) at 50 ng/mL, or medium alone. Medium with stimuli was supplemented with GolgiPlug containing Brefeldin A (1:2000). Ten minutes before the end of incubation, anti-mouse CD16/CD32 antibody was added to block non-antigen-specific binding of immunoglobulins to Fcγ receptors. In one time-point assessment, surface staining was performed by incubating re-stimulated cells with Pacific Blue (PB)-conjugated anti-mouse CD8 and peridinin chlorophyll protein complex (PerCP)-conjugated anti-mouse CD4 antibodies. After that, cells were fixed and permeabilized at room temperature for 20 min in 100 μL Cytofix/Cytoperm solution, washed with Perm/Wash buffer, and stained at 4 °C for 30 min with fluorescein isothiocyanate (FITC)-conjugated anti-IFN-γ, allophycocyanin (APC)-conjugated anti-IL-2, phycoerythrin (PE)-conjugated anti-IL-4, and phycoerythrin-cyanine dye 7 (PeCy7)-conjugated anti-TNF-α antibodies specific to murine cytokines. In the assessment of kinetics of cytokine response (Series II, Table 1), fixed and permeabilized cells were stained at 4 °C for 30 min with anti-murine Granzyme B conjugated to FITC (eBioscience, San Diego, CA, USA), perforin conjugated to PE (eBioscience, San Diego, CA, USA), IFN-γ conjugated to PECy7, and IL-2 conjugated to APC. Samples were processed on a FACS Canto flow cytometer (BD Biosciences, San Diego, CA, USA). Data analysis was carried using FlowJo software (Tree Star Inc, Ashland, OR, USA). A general lymphocyte area was defined and single living cells within this population were defined by their expression of CD4 or CD8 receptors, and further, by the production of IFN-γ, IL-2, IL-4, TNF-α, granzyme B, and perforin. Frequencies of CD8^+^ and CD4^+^ cells producing cytokines and/or cytolytic molecules in response to IN- or Luc-specific stimulation were quantified, and values for unstimulated cells were subtracted. Gating for the detection of (multi)cytokine expressing cells was performed as described previously [23]. 

### 2.18. ELISA for Anti-IN Antibodies

Anti-integrase antibodies were assessed in immunization Series I (Table 1). For this, Maxisorp 96-well microtiter plates (Nunc Maxisorp, Roskilde, Denmark) were coated with one of the IN protein variants (IN_a, IN_r1, IN_r2, IN_in, IN_in_r1, IN_in_r2, or IN of HIV-1 clade B HXB2 strain) diluted in PBS at 0.3 μg/mL and incubated overnight at 6–8 °C. Plates were washed six times with PBS containing 0.05% Tween-20. Individual mouse sera diluted step-wise from 1:100 in HIV-Scan Buffer (HSB; 2% normal goat serum, 0.5% BSA, 0.05% Tween-20, 0.01% sodium merthiolate) were applied and incubated overnight at 6–8 °C. Plates were washed as above and HRP-conjugated goat anti-mouse IgG antibody (Sigma) diluted in HSB was applied and incubated for 1.5 h at 37 °C. After incubation, plates were washed as above and color was developed with 3,3′,5,5′-tetramethylbenzidine substrate solution (TMB; Medico-Diagnostic Laboratory, Moscow, Russia). The reaction was stopped by adding 50 μL 2.5 M sulfuric acid, and optical density (OD) was measured at a dual wavelength of 450–620 nm. The cut-off for specific anti-IN antibody response at each time-point was set to the mean OD-values demonstrated by the preimmune sera of mice in the given group + 3SD. For positive sera showing OD values exceeding the cut-off, end-point dilution titers were established from the titration curves. Specific titers were presented as the end-point dilution titers minus the average end-point dilution titer exhibited by sera of mice receiving empty vector collected at the same time point. 

### 2.19. Toxicity Assessment

The toxicity of IN encoding plasmids was assessed in immunization Series III (Table 1). BALB/c mice were immunized with plasmids encoding IN_in, or IN_in_r1 and IN_in_r2 in a 1:1 (*w*/w) mixture, or empty vector pVax1, or PBS; all treatments followed by electroporation (Table 1). Assessment was done using Expanded Type 2 toxicity tests (https://www.fda.gov/media/72305/download) (accessed on 23 October 2018). The general health condition of the mice was evaluated by a veterinary doctor daily; an in-depth clinical examination of the parameters was performed, registered and scored weekly (Supplementary Table S7). Electroporation caused round-shaped burns 3–4 mm in diameter on the skin of all animals, which resolved before the first weekly examination. The condition of all animals at all timepoints was marked as normal. No differences were observed between study groups.

Body mass was measured daily after the first, at 1–3 day intervals after the second DNA immunization, and weekly thereafter (Supplementary Table S8). Two mice died during the experiment: one mouse in group IN_in died on day 12 after DNA prime, the cause of death unknown; one mouse in vector group did not recover from anesthesia. On day 36, 13 days after the boost, all remaining animals were humanely euthanized after 18 h of starvation. Blood samples were obtained immediately after by decapitation. Tissues were collected with subsequent measurement of the mass of lymph nodes, thymus, and liver (Supplementary Table S12), and assessment of bone marrow composition.

Complete blood count analysis included red blood cells (RBC), hematocrit (HCT), platelets (PLT), hemoglobin (HGB), white blood cells (WBC), lymphocytes (LYM), unclassified leukocytes (MID), and granulocytes (GRAN) (Supplementary Table S9). Analysis was performed using Мedonic СА-620 hematology analyzer (Boule Medical, Spånga, Sweden) according to manufacturer’s protocols. Two blood samples (one from group IN_in and one from vector group) did not pass quality check procedure on the hematology analyzer and were excluded from the analysis of complete blood counts. 

Biochemical blood analysis included measurement of the levels of alanine aminotransferase (ALT), aspartate aminotransferase (AST), alkaline phosphatase (ALP), lactate dehydrogenase (LDH), total protein (TP), albumin (ALB), urea (UREA), glucose (GLC), cholesterol (CHOL), triglycerides (TG), sodium (Na), and potassium (K) (Supplementary Table S10). Analysis was performed using Stat Fax 4500+ biochemical analyzer (Awareness Technology, Palm City, FL, USA) according to manufacturer’s protocols. Four serum samples (one from each group) did not pass quality check procedure of the biochemical analyzer and were excluded from the biochemical blood analysis. 

Bone marrow smears were stained by Romanowsky-Giemsa procedure, and cell composition was assessed by microscopy [66] (Supplementary Table S11). 

### 2.20. Challenge of IN DNA-Immunized Mice with IN-Expressing 4T1luc2 Subclones 

Protective potential of IN DNA immunization was assessed in immunization Series IV (see Table 1 for details). Twelve days after the 2-nd DNA injection, mice were challenged with 4T1luc2_IN_a_1.2, 4T1luc2_IN_a_r2_1.5, or parental 4T1luc2 cells suspended in 50 μL of RPMI-1640 in amount of 10^4^ cells per injection site. Cells were implanted subcutaneously into the left and right flanks using a 25G needle mounted on an insulin syringe (B Braun, Melsungen, Germany). Injections were placed 1.0 to 1.5 cm apart from the site of DNA immunization. Tumor growth was monitored by in vivo BLI (Spectrum CT) and presented as a total photon flux from individual implantation sites. First BLI was performed directly after the implantation, and following, every 2–3 days until the size of the tumors in the control mice reached a plateau (by day 19 after the booster injection). At this point, mice were sacrificed, primary tumors, lungs, livers, and spleens were dissected and assessed for infiltrating tumor cells by ex vivo BLI as described earlier [28]. After that, each spleen was split into halves; one half was washed from luciferin with PBS and used to prepare splenocytes for T cell tests. The other, as well as livers and lungs, were fixed with 0.4% paraformaldehyde for 24 h, washed with PBS, and casted into paraffin. FFPE blocks were stored at room temperature for further use in histochemical analysis. In vivo, ex vivo BLI assessments and histochemical analysis were done as described in sections on implantation of tumor, with follow up of tumor growth, and histology and ex vivo assessment of the metastases. 

### 2.21. Statistics and Software

Data were presented as individual entries, or expressed as median values or as mean ± standard deviation (SD). Graphical presentation of the data and statistical calculations were performed using Microsoft Excel, GraphPad Prism version 8.0.0 software (GraphPad Software, San Diego, CA, USA), STATISTICA AXA 10.0 software (StatSoft Inc., Tulsa, OK, USA), and Statistical Package for Social Sciences (IBM SPSS, version 17.0, Armonk, NY, USA). Significance of the differences between in vitro parameters were analyzed using non-parametric Mann–Whitney test and Wilcoxon paired-sample test. Continuous but not normally distributed variables, such as the antibody levels, number of cytokine-producing spot-forming cells, radiance per area, and different parameters of clinical and biochemical blood analysis, bone marrow composition, and organ mass were compared by the non-parametric F-test, and Kruskal–Wallis and Mann–Whitney *U* tests, with Bonferroni or Holm and Hochberg corrections applied for multiple comparisons. Comparisons of overall profile of in vitro and in vivo properties of immunogens were performed by Friedman ANOVA test with Kendall coefficient of concordance with subsequent Sign-test. Statistical analysis of body mass data was done using mixed-effects model with Geisser-Greenhouse correction followed by Tukey’s multiple comparisons of data from individual time points. Linear correlations between variables were analyzed using the Spearman rank-order test. *p* values <0.05 were considered as significant. 

## 3. Results

### 3.1. Design of DNA Immunogens Encoding Integrase Variants Resistant to RAL

**Figure 1:**
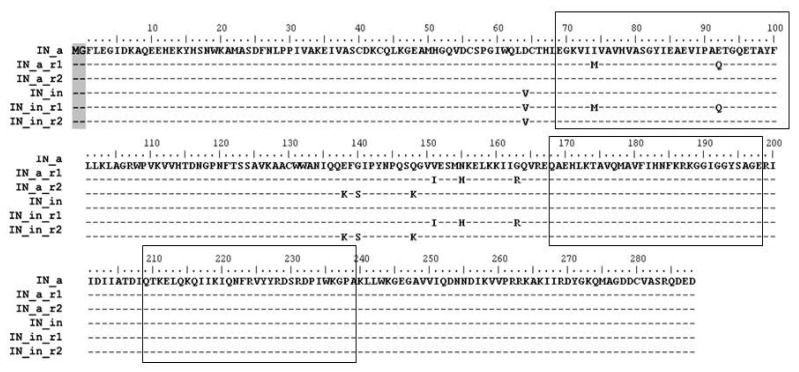
Consensus integrases of HIV-1 clade A FSU_A strain and their enzymatically active and inactivated variants with mutations of resistance to RAL. Amino acid sequences of the consensus IN of HIV-1 subtype A (IN_a), RAL-resistant INs with mutations L74M, E92Q, V151I, N155H, G163R (IN_a_r1) or E138K, G140S, Q148K (IN_a_r2) [25] and their inactivated variants with D64V mutation (IN_in, IN_in_r1, IN_in_r2). All IN variants carry Met-Gly dipeptide on the N-terminus (shadowed) originating from introduction of the Kozak sequence at the 5′-terminus of IN gene. Amino acid numeration is started at Phe as the 1-st amino acid in the wild-type IN. Boxes mark clusters of B- and T-cell epitopes of IN recognized in different species (see Supplementary Figure S1 for details).

We have earlier designed a synthetic gene encoding a consensus integrase of HIV-1 subtype A strain FSU_A circulating in the former Soviet Union (IN_a) [23] (Figure 1). Using this gene as a platform, we further designed IN variants harboring two sets of mutations conferring resistance to RAL [25], one with primary resistance mutation N155H and secondary mutations L74M, E92Q, V151I, G163R (IN_a_r1); and the other, with primary resistance mutation Q148K and secondary mutations E138K and G140S (IN_a_r2) [5,67]. Both integrases were enzymatically active, and insensitive to inhibitors of strand transfer at a cost of a 30–90% loss of the efficacies of both strand transfer and 3′-processing [25]. 

Here, we subjected genes of IN variants to site-directed mutagenesis introducing D64V mutation to abrogate both LTR processing and joining activities deemed harmful to expressing cells [23,68,69], generating integrase variants IN_in_r1 and IN_in_r2 (Figure 1). All IN variants were expressed in *E coli*, purified, and subjected to in vitro tests of the 3′-processing and strand transfer activities. The tests demonstrated that D64V mutation completely abrogated specific IN enzymatic activities (Supplementary Figure S5a,b; IN_in_r1 variant retained a non-specific exonuclease activity, Supplementary Figure S5c). Thus, both IN variants were safe to use as DNA immunogens. We recloned DNA encoding all IN variants into eukaryotic expression vector pVax1 generating plasmids directing expression of active (pVaxIN_a, pVaxIN_a_r1, pVaxIN_a_r2) and inactivated integrases (pVaxIN_in, pVaxIN_in_r1, pVaxIN_in_r2) (Figure 1).

Further, we composed an epitopic map of IN bringing together sequences of B- and T-cell epitopes of IN recognized by humans, primates and mice (extracted from IEDB database and original publications referred in Materials and Methods; Supplementary Figure S1). Analysis identified three epitopic clusters localized at aa 69–100, 168–198, and 209–239 of integrase (Supplementary Figure S1; “boxed” in Figure 1). Respective regions were represented by synthetic peptides (Supplementary Table S1) to be used in screening of immune response induced by IN DNA immunization.

### 3.2. IN Variants Are Efficiently Expressed in Eukaryotic Cells 

**Figure 2:**
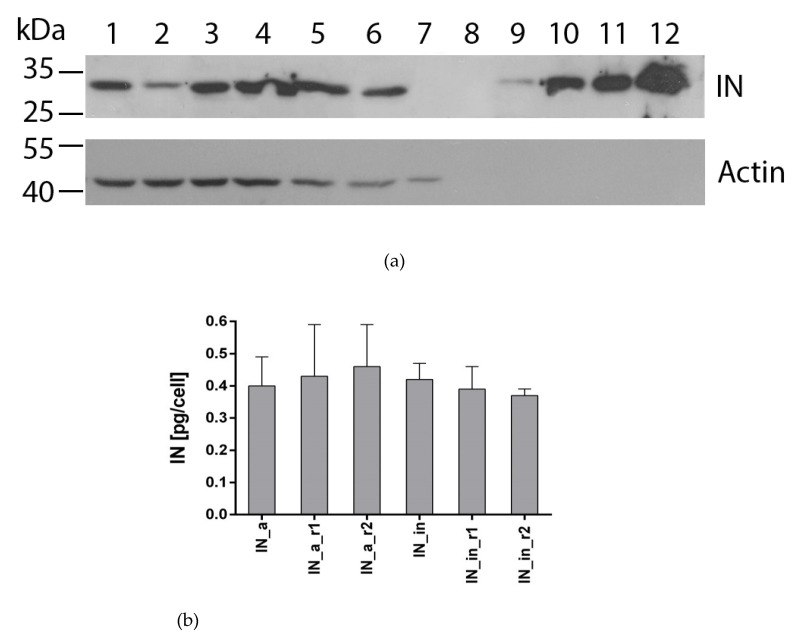
Expression of IN variants in eukaryotic cells. Western blotting of the lysates of HeLa cells transiently transfected with the pVaxIN_a (lane 1), pVaxIN_a_r1 (lane 2), pVaxIN_a_r2 (lane 3), pVaxIN_in (lane 4), pVaxIN_in_r1 (lane 5), pVaxIN_in_r2 (lane 6), or empty vector pVax1 (lane 7); Page Ruler Prestained Protein Ladder (Fermentas) (lane 8); recombinant IN of LAV/BRU strain of HIV-1 loaded in the amounts of 0.5, 2.5, 5, and 10 ng/well (lanes 9 to 12, respectively) (**a**); average amount of IN variant expressed per transfected HeLa cell assessed by ImageJ (**b**). Western blotting was done with murine monoclonal anti-IN antibody (IN-2 #ANT0071, Diatheva, Cartoceto, Italy), and blots were stripped and re-stained with the monoclonal anti-actin antibodies. (**b**) The results of two independent runs, each done in duplicate, mean± SD. Levels of expression of IN variants did not differ (*p* > 0.1; Kruskal–Wallis test).

To evaluate the level of expression of IN variants in eukaryotic cells, we transiently transfected human (HeLa, HEK293T) or murine (NIH3T3) cells with plasmids encoding active (pVaxIN_a, pVaxIN_a_r1, pVaxIN_a_r2) or inactivated IN variants (pVaxIN_in, pVaxIN_in_r1, pVaxIN_in_r2). Western blot with monoclonal anti-IN antibodies demonstrated that all INs were expressed at the level of 0.4–0.6 pg per cell independently of the origins of expressing cell, (Figure 2, Supplementary Figure S6) in line with our earlier observations for EVG resistant integrases [23]. 

### 3.3. IN Variants Demonstrate a Mixed Proteasomal/Lysosomal Pattern of Proteolytic Degradation 

**Figure 3:**
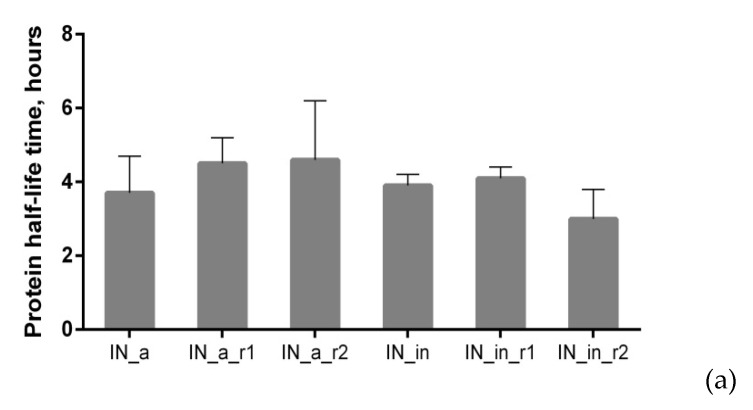

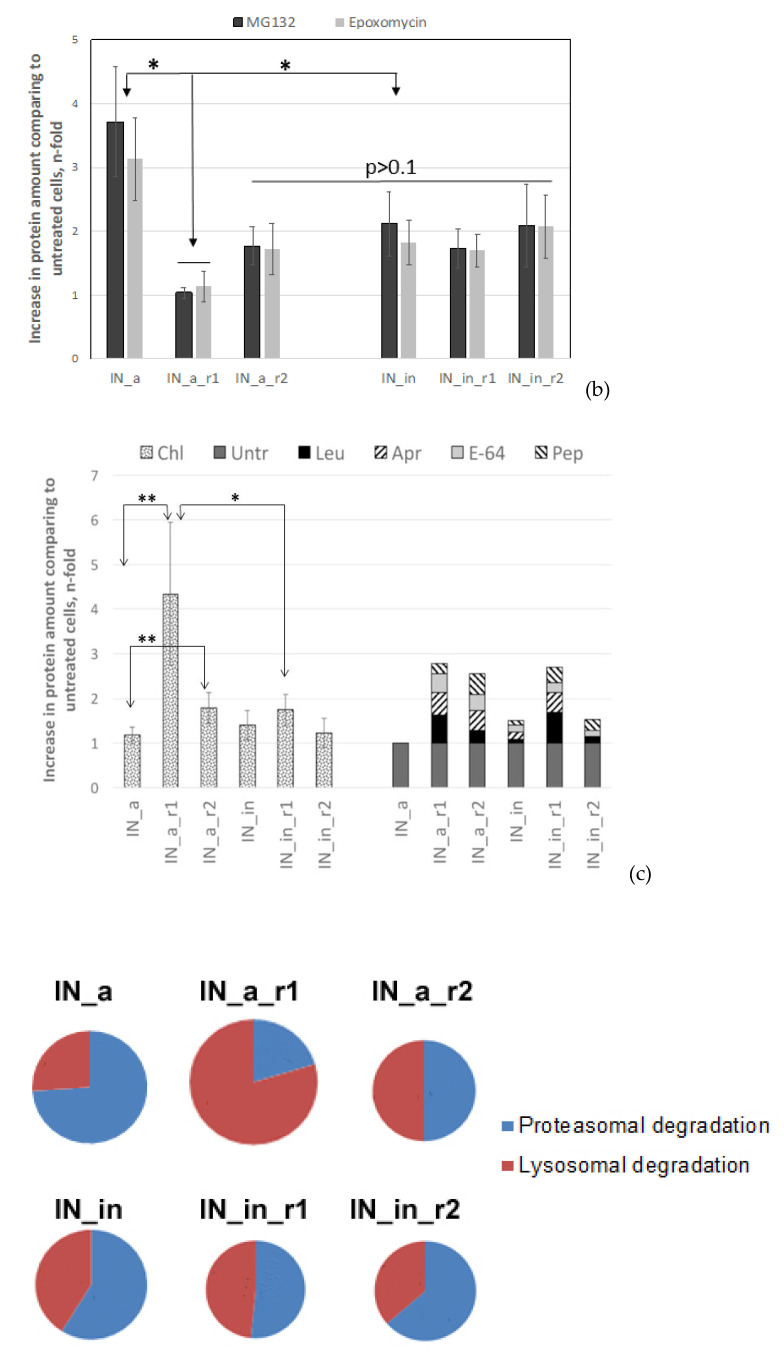

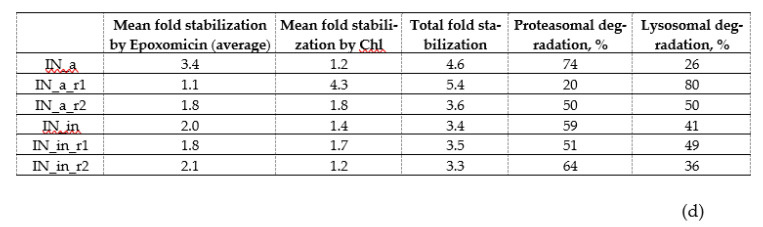
Proteolytic degradation of IN variants. Half-life of IN variants determined by pulse-chase (**a**); degradation pathway probed using proteasome (**b**) and Lysosome (**c**) inhibitors; proportion of proteasomal and lysosomal degradation patterns in the overall IN degradation (**d**). HeLa cells were transiently transfected with pVaxIN_a, or pVaxIN_a_r1, or pVaxIN_a_r2, or pVaxIN_in, or pVaxIN_in_r1, or pVaxIN_in_r2, and subjected to cycloheximide chase to determine half-life (**a**), or 48 h post-transfection treated with proteasome inhibitors MG132 (10 μM) or Epo (0.5 μM) (**b**), or lysosome inhibitors chloroquine (Chl; 10 μM), leupeptin (Leu; 10 μg/mL), aprotinin (Apr; 100 μg/mL), pepstatin (Pep; 5 μg/mL), or E-64 (10 μМ), or left untreated (“Untr”) (**c**). Graphs (**b**,**c**) demonstrate the relative amount of IN protein in samples incubated with MG132 and epoxomicin (**b**) or with chloroquine ((**c**), left panel) or with inhibitors of individual classes of proteases active in the lysosomal compartment represented as a pile-up of the effects of individual inhibitors ((**c**), right panel) compared to the untreated samples. (**d**) Size of the circles is proportional to the total increase in the content of IN variants achieved after application of proteasome (Epo) and lysosome (Chl) inhibitors (total fold stabilization). Sectors of the circles show contributions of Epo and Chl inhibitors to the total stabilization of each IN variant, in %. The values used for the plotting of pie diagrams are indicated in the table below. Data represent the results of at least three independent experiments. IN variants demonstrated no significant difference in half-life in the CHI (**a**), or in response to treatment with MG132 versus Epoxomicin (**b**), or in response to treatment with Chl as compared to sum of treatments with individual inhibitors of lysosomal proteases (**c**) (*p* > 0.05, Mann–Whitney test). *—*p* < 0.05, **—*p* < 0.01, difference between individual IN variants indicated on (**b**,**c**). The differences that were not shown on the (**c**) for the IN variants treated with Chl: IN_a_r2 versus IN_in_r2, *p* < 0.05; [IN_a, IN_in, and IN_in_r2] versus [IN_a_r1, IN_a_r2, and IN_in_r1], *p* < 0.01; IN_a_r1 versus all the other IN variants, *p* < 0.01 (**c**). Statistical analysis was done first by Kruskal–Wallis test and then by Mann–Whitney test.

We examined proteolytic stability of IN variants by assessing their half-life using cycloheximide (CHI)-chase assay [70]. IN variants had similar half-lives in the range of 3.7 to 4.6 h (Figure 3a; examples of Western blots from the cycloheximide chase are presented in Supplementary Figure S7).

Further, we determined if drug resistance (DR) and inactivation mutations have any specific impacts on the pathways of intracellular degradation of IN variants. For this, we treated HeLa cells transiently transfected with IN-encoding plasmids, with proteasomal inhibitors MG132 or epoxomicin, or with a panel of lysosomal inhibitors, and analyzed the accumulation of IN variants by Western blotting (Figure 3b,c; examples of Western blots illustrating degradation in the presence of proteasomal and lysosomal inhibitors are given in Supplementary Figure S8a,b). Enzymatically active IN_a was predominantly degraded by proteasome, as could be seen from its 3–4-fold stabilization by both MG132 and epoxomicin (Epo) [71] (Figure 3b, Supplementary Figure S8a). We saw no difference between the effects of MG132 and Epo (a more specific proteasome inhibitor epoxomicin than MG132 [70]) confirming that stabilization by MG132 was due to the specific inhibition of the proteasomal route of IN degradation. Incorporation of inactivation mutation and RAL resistance mutations into IN_a led to a two-fold decrease in the stabilization by both MG132 and Epo (Figure 3b, Supplementary Figure S8a). As overall stability of mutant IN variants was retained (Figure 3a), this indicated a shift to lysosomal route of degradation. IN_a_r1 was the least sensitive to proteasomal degradation, and sensitivity to proteasomal degradation of other mutants was equal (Figure 3b; Supplementary Figure S8a). 

Lysosomal degradation was probed by chloroquine, and by the inhibitors of individual classes of proteases active in the lysosomal compartment: aprotinin inhibiting the serine; pepstatin, the aspartic acid; and E-64, the cysteine; and leupeptin inhibiting the serine and cysteine proteases. Each of the inhibitors of individual classes of lysosomal proteases exerted a weak stabilizing effect, their combined effect was equal to the effect of chloroquine (Figure 3c). Total stabilizing effect of lysosome protease-specific inhibitors and stabilizing effect of chloroquine were highly correlated (Supplementary Figure S9). Active consensus integrase IN_a was insensitive to the inhibitors of lysosomal proteolysis (Figure 3c; Supplementary Figure S8b). Inactivation mutation D64V had no effect on the lysosomal degradation of the parental IN_a (Chl and individual inhibitors of lysosomal proteases had similar effect on IN_in as on IN_a; *p* > 0.05 in Mann–Whitney test; Figure 3c, Supplementary Figure S8b). Incorporation of both of RAL resistance mutation patterns enhanced IN_a degradation by the lysosome: treatment with lysosomal inhibitors caused a 2- to 4-fold increase in the content of IN_a_r1 and of IN_a_r2 compared to IN_a (*p* < 0.05; Figure 3c; Supplementary Figure S8b). However, introduction of D64V led to a 2-fold decrease in protein stabilization by the lysosomal inhibitors, i.e., partially reversed the lysosomal re-routing (Figure 3b,c, Supplementary Figure S8b). 

Stabilization of IN variants by proteasome and lysosome inhibitors was then represented as a pie diagram in which the overall fold stabilization of IN variant by the proteasomal (Epo) and lysosomal inhibitor (Chl) was taken for 100%, and effect of each class of inhibitors was represented as % of the total (Figure 3d). For example, for IN_in, we observed 2-fold stabilization by Epo, and 1.4-fold stabilization by chloroquine compared to untreated enzyme variant; overall fold stabilization was 3.4, and the input of each of the pathways, 59% (2/3.4) and 41% (1.4/3.4), respectively (Figure 3d). The size of the pie was made proportional to the total stabilization fold. This graphical representation demonstrated that IN_in_r1 was the most stable, and IN_a_r1 was the least stable, predominantly degraded by the lysosomal proteases (Figure 3d). Other variants had comparable proteolytic stability, with an equal contribution of degradation by lysosome and proteasome (Figure 3d). 

### 3.4. Eukaryotic Expression of IN Variants Induces Production of Reactive Oxygen Species

**Figure 4:**
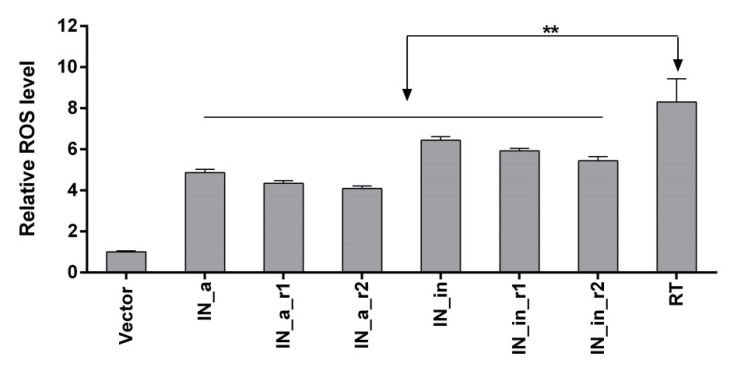
Transient expression of IN in eukaryotic cells induces moderate levels of ROS. HEK293T cells were transfected with pVax1-based plasmids expressing IN_a, IN_a_r1, IN_a_r2, IN_in, IN_in_r1, and IN_in_r2 or HIV-1 reverse transcriptase of clade B (HXB2) [15,72]. Production of ROS was detected using sensor dye 2′,7′-dichlorodihydrofluorescein diacetate (DCFH-DA). The level of fluorescence emitted by reaction products of DCFH-DA and ROS in IN and RT expressing cells were normalized to those in the cells transfected with empty vector pVax1. Data represent the results of two independent experiments, each done in triplicate, mean +SD. Results are compared using Kruskal–Wallis and Mann–Whitney tests; difference between different IN variants, *p* > 0.1; difference between IN variants and HIV-1 RT, **—*p* < 0.01 (Kruskal–Wallis, Mann–Whitney tests).

Capacity to induce ROS is an important protein property shaping its immunogenicity [15,72], but their excess could be harmful [73]. Having this in mind, we evaluated the level of oxidative stress induced by IN variants when expressed in transiently transfected eukaryotic cells by measuring the production of ROS captured by fluorogenic dye 2′,7′-dichlorodihydrofluoresceine diacetate (DCFH-DA). DCFH-DA penetrates the cells, gets de-esterified DCFH-DA into DCFH which is oxidized by different types of ROS generating fluorescent product dichlorofluorescein (DCF) with emission of fluorescence signal proportional to the levels of ROS [74]. Expression of all IN variants induced 4- to 6.5-fold increase in DCF fluorescence/levels of ROS in expressing HEK293T cells compared to cells transfected with empty vector, with no significant difference between individual IN variants (*p* > 0.1, Figure 4). The level of ROS was 25 to 50% lower than levels induced by transient expression of HIV-1 reverse transcriptase (Figure 4), which we have earlier shown to induce high levels of ROS in eukaryotic cells of different origins [15,72]. 

### 3.5. Expression of IN Variants does Not Change the Tumorigenic or Metastatic Potential of Murine Adenocarcinoma Cells

Integrase activity is genotoxic [69]. We launched an experiment to find if this is the case for HIV-1 IN, testing the extreme case of overexpression of enzymatically active integrase variants. For this end, we created subclones of the murine mammary gland adenocarcinoma cells 4T1luc2 expressing IN variants and compared their properties with the properties of the parental cell line. Lentiviral vectors were designed encoding enzymatically active IN variants (Supplementary Figure S2) and used to transduce 4T1luc2 cells at multiplicity of infection (MOI) 1, 5, and 20. Transduction experiments demonstrated that IN variants were toxic to the expressing cells. The level of toxicity depended on IN variant (Supplementary Figure S3). Lentiviruses expressing non-mutated IN_a were toxic at MOI > 1, and DR IN, at MOI > 5, registered in the form of massive cell death and small size of the rare viable colonies (compare Supplementary Figure S3a to Supplementary Figure S3c,e, and Supplementary Figure S3b,d to Supplementary Figure S3f, respectively). Thus, we obtained subclones expressing IN_a and IN_a_r2 (4T1luc2_IN_a_1.2 and 4T1luc2_IN_a_r2_1.5, respectively). 

**Figure 5:**
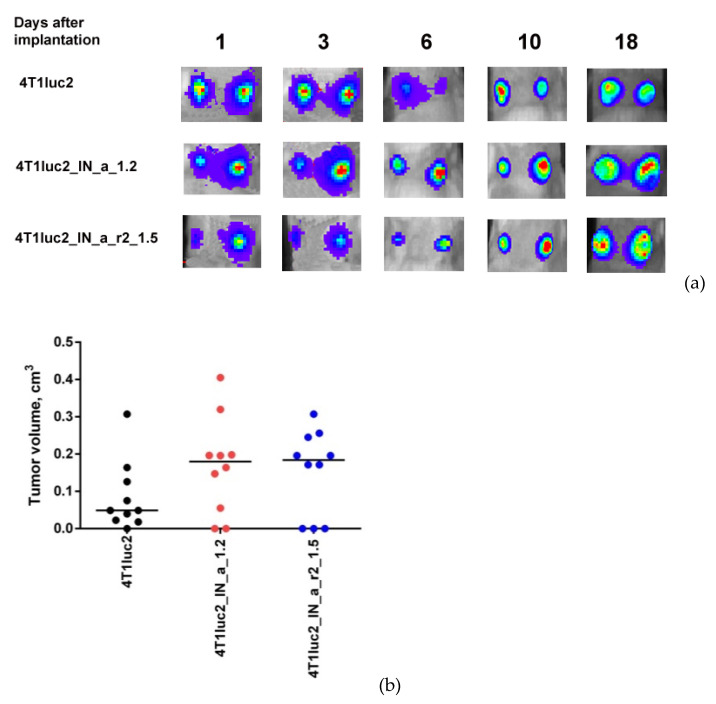

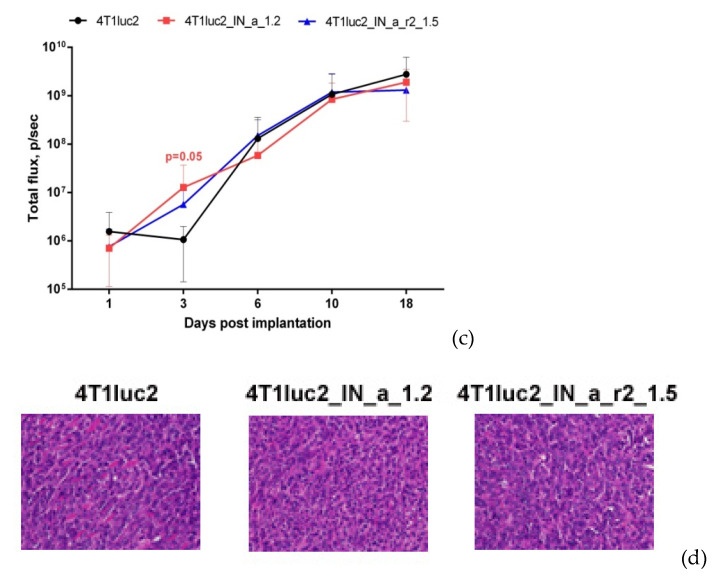
Stable expression of enzymatically active consensus HIV-1 clade A integrase with or without drug resistance mutations has no effect on the tumorigenic potential of murine mammary gland adenocarcinoma 4T1luc2 cells. Bioluminescent images of areas of implantation of tumor cells by in vivo bioluminescence showing growth of representative tumors formed by 4T1luc2 cells (parental) and 4T1luc2_IN_a_1.2 and 4T1luc2_IN_a_r2_1.5 clones (**a**); quantification of photon flux from the areas of implantation of tumor cells monitoring tumor growth (**b**); palpable tumor size at the experimental end-point by day 19 (**c**); histochemical characterization of the solid tumors formed by 4T1luc2, IN_a expressing 4T1luc2_IN_a_1.2, and IN_a_r2 expressing 4T1luc2_IN_a_r2_1.5 cells (**d**). Scales on the right side of bioluminescent images demonstrate the intensity of BLI in color. Growth of tumors was assessed as the average total photon flux (*p*/sec) from the sites of implantation on days 1–18 post-implantation; (**b**) data represent mean ± SD; (**c**) line bars represent the median values. Images of H&E stained tumor sections were done at magnification × 400 by Leica DM500 camera (Wetzlar, Germany). Statistical comparison was done by Kruskal–Wallis and Mann–Whitney tests and did not detect significant differences between the groups (*p* ≥ 0.05), except for the tendency for difference between 4T1luc2_IN_a_1.2 and parental 4T1luc2 cells on day 3 after implantation (**b**).

Subclones were assessed for in vivo tumorigenicity. For this, BALB/c mice were ectopically implanted with 10^4^ 4T1luc2_IN_a_1.2 and 4T1luc2_IN_a_r2_1.5 cells into two sites, and followed until the signs corresponding to the humane endpoint (3 weeks post-injection). The rate of tumor growth was monitored by in vivo bioluminescence imaging (BLI) starting from the day of implantation (Figure 5a). BLI demonstrated that tumors formed by IN expressing clones and by parental 4T1luc2 cells grew with the same rate (Figure 5a,b). Morphometric measurements done at the experimental endpoint demonstrated that tumors formed by IN expressing 4T1luc2 cells were somewhat larger than tumors formed by the parental cell line; however, the difference was not statistically significant (Figure 5c). 

Microscopic examination of H&E stained formaldehyde-fixed paraffin-embedded (FFPE) blocks prepared from IN_a and IN_a_r2 expressing tumors revealed that all tumors were high grade (G3) poorly differentiated adenocarcinomas with mostly epithelioid or mixed epithelioid and sarcomatoid appearance, well vascularized, with increased nuclear pleomorphism and stromal desmoplasia (Figure 5d). Tumors formed by the parental 4T1luc2 cells exhibited multifocal necrosis, and tumors formed by IN-expressing 4T1luc2 cells exhibited wide areas of necrosis localized in center of the tumors (Figure 5a). Single tumors of each type were excised and used to confirm the presence of IN encoding inserts in genomic DNA (see Supplementary Figure S10 for illustration) and to measure the expression of IN mRNA. Analysis of total RNA extracted from tumor tissues by real-time PCR demonstrated similar levels of expression in tumors of mRNA of IN_a and IN_a_r2 (Supplementary Table S6). Thus, overall, subclones of murine adenocarcinoma cells expressing active IN variants did not differ in growth properties from the parental tumor cells, i.e., expression of enzymatically active integrase variants did not enhance their tumorigenic potential. 

**Figure 6:**
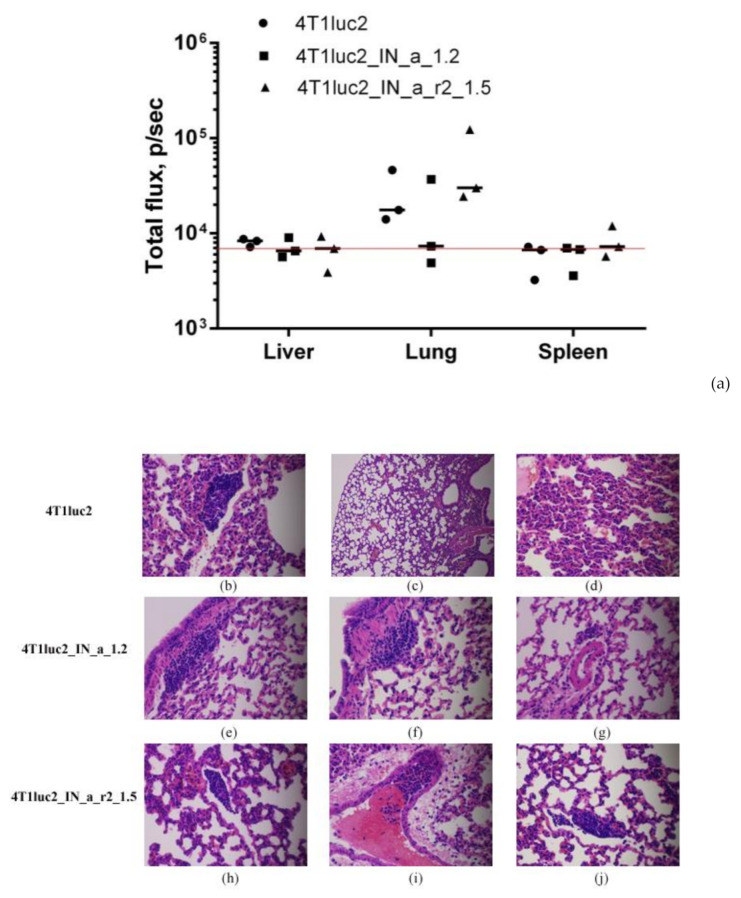
Murine adenocarcinoma 4T1luc2 cells expressing HIV-1 integrase have low metastatic activity. Infiltration of tumor cells into mouse organs (**a**) and histochemical characterization of lungs of mice implanted with the parental 4T1luc2 cells and their IN expressing derivatives (**b**–**j**) showing single metastases. Infiltration of Luc-expressing cells into organs was assessed by ex vivo BLI of the liver, lungs, and spleen; values represent a median of total flux from three organ samples (*p*/sec); red line intercepts the background bioluminescence characteristic to one Luc-expressing 4Tl cell (**a**). H&E staining of FFPE sections of the lungs of mice implanted with 4T1luc2 (**b**–**d**), 4T1luc2_IN_a_1.2 (**e**–**g**), 4T1luc2_IN_a_r2_1.5 (**h**–**j**). Magnification ×100 (**c**), and ×400 elsewhere. Slides were examined and images were created using Leica DM500 microscope (Wetzlar, Germany) equipped with a digital camera. No significant difference was detected between organ infiltration in mice implanted with IN-expressing and parental 4T1luc2 cells (*p* > 0.1, Kruskal–Wallis, Mann–Whitney tests).

Next, we evaluated if expression of IN could influence the metastatic potential of tumor cells. For that, we measured ex vivo total photon flux emitted from the organs at the experimental end-point, as we have previously shown that it adequately represents the number of organ infiltrating tumor cells expressing luciferase and can be used as high throughput approach of assessing the metastatic activity [28]. At the experimental endpoint, we excised lungs, livers, and spleens of mice implanted with IN-expressing and parental 4T1luc2 cells and subjected them to ex vivo BLI. Total flux exceeded the signal level ascribed to one Luc-expressing 4T1 cell (6000 photons/sec; Reference [28]) was detected only in the murine lungs (Figure 6a). BLI could not reliably register photon emission from either liver or spleen (Figure 6a), indicating that these organs were infiltrated with very few tumor cells, corroborating our earlier findings of a compromised metastatic potential of Luc expressing 4T1 cells [60]. Total flux emitted from lungs of mice bearing IN expressing tumors did not differ from that for mice with tumors induced by 4T1luc2 (*p* > 0.1; Figure 6a). We performed histological assessment of H&E sections of FFPE blocks prepared from the lung tissues to find single metastases in the lungs of mice in all three groups (Figure 6 b–j; on the average 1 ± 0,5 per mouse). Metastases were constituted by single tumor cells surrounded by severe inflammatory infiltrates, specifically pronounced in mice implanted with parental 4T1luc2 cells (as in Figure 6b–d,i). The inflammatory pattern had a multifocal rather than a diffused appearance (Figure 6c), resembling the acute interstitial pneumonia affecting alveolar septa (http://www.pathologyoutlines.com/topic/lungnontumoracuteinterstitialp.html) (accessed on 30 May 2017). Overall, the subclones of murine adenocarcinoma cells expressing active IN variants did not differ from the parental tumor cells in the ability to infiltrate organs of mice, form distal metastases, cause infiltration of immune cells with inflammation. Thus, expression on even enzymatically active HIV-1 IN by tumor cells had no effect on the in vivo growth or metastatic potential of tumor cells. Accordingly, the use of HIV-1 integrase as DNA immunogen is not associated with the risk of enhancing the tumorigenic/carcinogenic potential of expressing cells, opening the gate for DNA immunization experiments.

### 3.6. Integral Immune Response against INs Assessed by Bioluminescent Imaging

**Figure 7:**
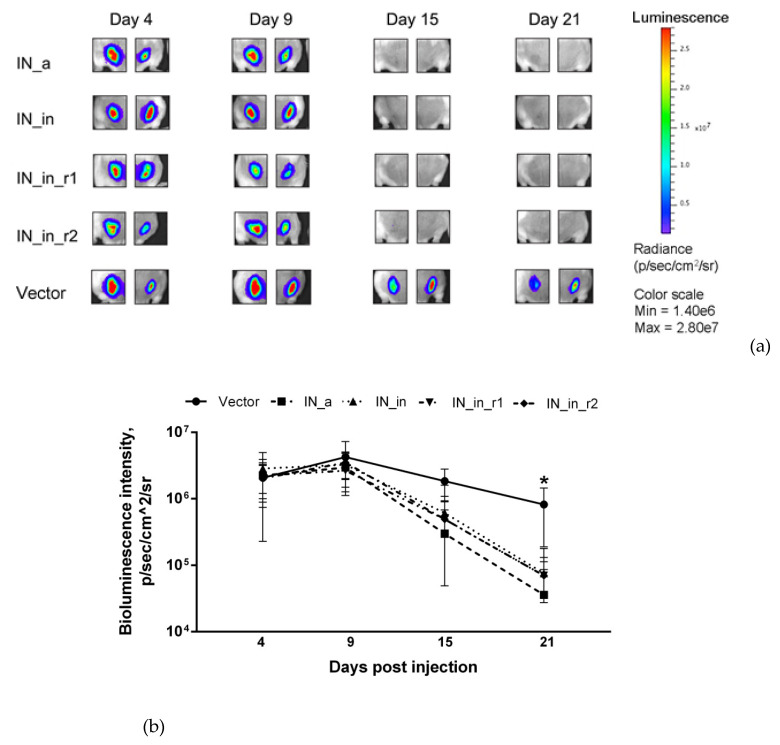
Immunization of mice with the plasmids encoding IN variants mixed with the reporter plasmid pVaxLuc results in rapid loss of photon flux from the sites of immunization. In vivo monitoring of luciferase activity at days 4, 9, 15, and 21 after the administration of plasmids encoding the consensus IN (IN_a), inactivated consensus IN (IN_in), inactivated consensus IN with raltegravir resistance mutations (IN_in_r1, IN_in_r2), or empty vector pVax1 (Vector), each mixed with Luc reporter gene (1:1) (**a**); change of photon flux reflecting Luc expression over time (**b**). Images demonstrate two representative injection sites per group followed throughout the immunization. The scale to the right represents the level of luminescent signal in pixels/sec/cm^2/sr. (**b**) Curves represent mean ± SD (*n* = 8) (Table 1, Series I, II). *—*p* < 0.05 for BLI in vector-immunized mice versus all IN variants-immunized mice. Statistical analysis was performed by Kruskal–Wallis and Mann–Whitney tests.

First, we assessed the integral immune response against IN variants by in vivo BLI. For this, we immunized mice with the plasmids encoding IN variants mixed with plasmid encoding Luc (Table 1, Series I, II) and monitored the levels of photon flux from the sites of immunization. We have earlier shown that introduction of a mixture of potent DNA immunogen with Luc-encoding plasmid results in immune clearance of immunogen/Luc co-expressing cells registrable as an accelerated loss of total photon flux from the sites of immunization [75]. Here, we immunized mice with the plasmids encoding IN variants mixed with the plasmid encoding Luc (Table 1, Series I, II). DNA immunization with IN_a, IN_in, IN_in_r1, and IN_in_r2 resulted in a complete loss of photon flux from immunization sites by day 15, whereas photon emission in control mice receiving Luc DNA mixed with empty vector continued up to day 21 (Figure 7a,b). IN variants demonstrated equal capacity to reduce reporter expression (*p* > 0.1), indicating similar integral immunogenicity. Primary assessment of IN immunogenicity and of the specificity of anti-IN immune response was done in mice receiving single DNA immunization with inactivated IN variants IN_in, IN_in_r1, and IN_in_r2, and parental IN_a for comparison (Table 1, Series I, II). 

### 3.7. DNA Immunization with IN Variants Induces Cross-Reactive Antibody Response 

**Figure 8:**
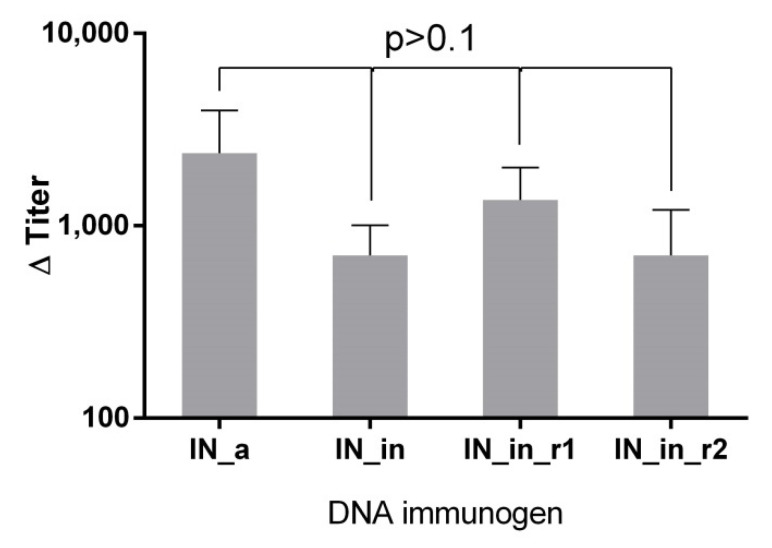
DNA immunization with IN gene variants induces IN-specific antibody response. Endpoint titers of IgG against IN variant used in immunization in the sera of BALB/c mice DNA-immunized with consensus HIV-1 FSU-A integrase (IN_a), consensus IN inactivated by D64V mutation (IN_in) and consensus inactivated integrases carrying mutations conferring resistance to raltegravir (IN_in_r1, IN_in_r2). Data represent the mean of two independent ELISA runs, each done in duplicate. Values on Y-axis (Titer bar) represent the mean end-point antibody titers of individual mice DNA immunized with IN variants with subtracted mean end-point antibody titer of the vector group, +SD. No statistically significant differences were detected between endpoint titers of sera of mice immunized with different IN genes variants, *p* > 0.1 (Mann–Whitney test with Holm multiple comparisons correction).

To assess antibody response, sera of mice DNA immunized with IN variants (Table I, Series I) were analyzed for the presence of total anti-IN IgG by indirect ELISA. Firstly, we evaluated antibody response with specificity to IN proteins encoded by the plasmids used for immunization. Anti-IN IgG in the average titer 10^3^ were detected in all groups (*p* > 0.1); the lowest antibody titers were found in IN_in-immunized mice, but the difference did not reach the level of significance (Figure 8). 

We have also evaluated the cross-reactivity of IN-specific antibody response, i.e., if mice were able to recognize enzymatically active integrases, corresponding to variants expressed in HIV-1 infection, IN_a, IN_a_r1, IN_a_r2, and also IN of HIV-1 of subtype B HXB2 (IN_B). DNA-immunized mice had a similar level of antibodies recognizing enzymatically active IN variants in titers 1–2×10^3^ (*p* > 0.1), with somewhat weaker recognition of subtype B integrase IN_B (Supplementary Figure S11a–d). Interestingly, the overall humoral immunogenicity of IN_a, IN_in, and IN_r1 was similar, while IN_in_r2 tended to induce a weaker total antibody response (Supplementary Figure S11e,f; *p* = 0.09). Altogether, IN immunizations stimulated moderate IN-specific antibody response effective in cross-recognition of different variants of IN proteins of subtype A, including active and inactive, wild type, and drug resistant variants. 

### 3.8. Immunization with IN Genes Induces Potent Cellular Immune Response with a Lytic Potential 

**Figure 9:**
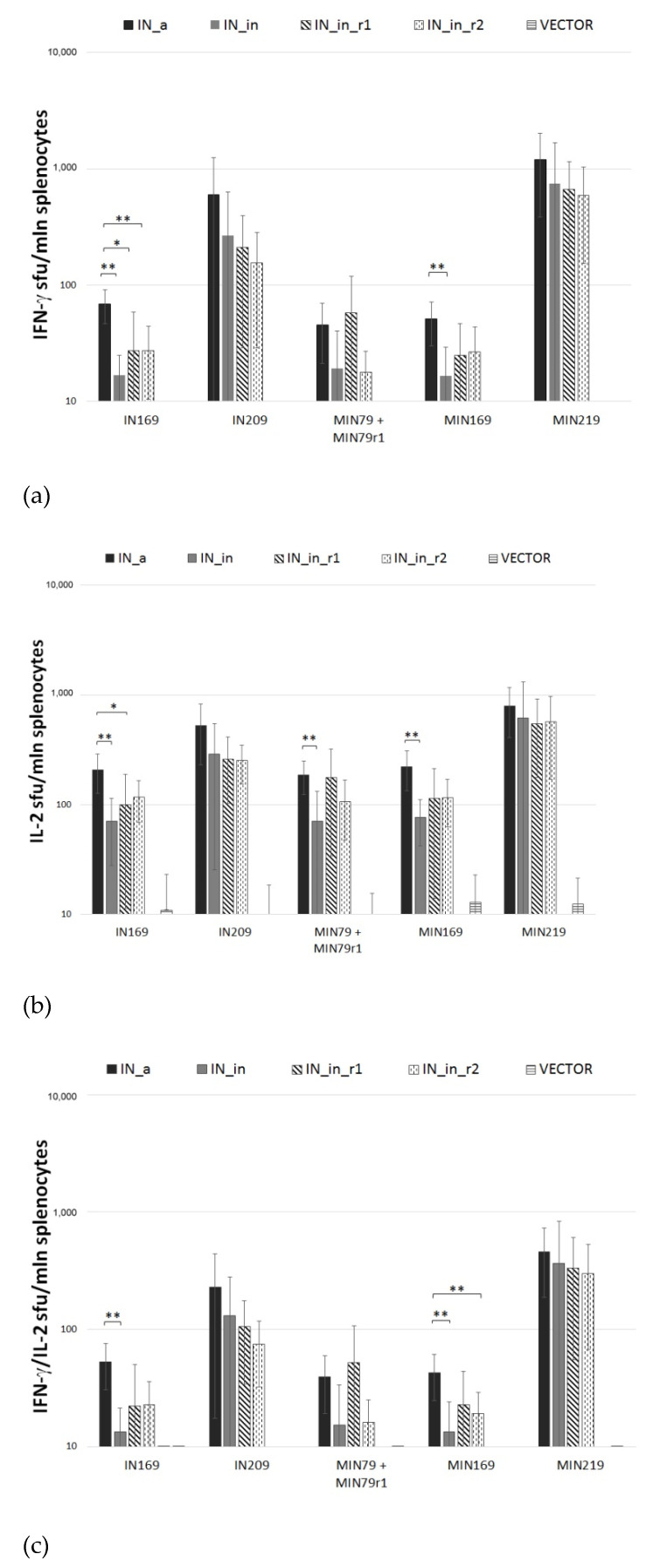
Splenocytes of mice DNA immunized with IN variants produce IFN-γ/IL-2 in response to stimulation with IN peptides in Fluorospot assay. Mice (*n* = 4) were immunized with single injections of pVax1-based plasmids encoding consensus IN of HIV-1 clade A (IN_a), inactivated IN (IN_in), inactivated IN with mutations conferring resistance to raltegravir (IN_in_r1, IN_in_r2), or empty vector (Table 1, Series I). Splenocytes of mice were stimulated in vitro for 18 h with individual or pooled IN-derived peptides representing known human (IN series) and murine (MIN series) epitopes (Supplementary Table S1). In vitro secretion of IFN-γ (**a**), IL-2 (**b**), and dual secretion of IFN-γ/IL-2 (**c**) was measured as the number of signal-forming units (sfu) per mln splenocytes. Data, the average number of sfu/mln splenocytes, ±SD, represent the results of two independent runs, each done in duplicate. **—*p* < 0.01; *—*p* < 0.05 (Mann–Whitney test, Holm/Hochberg multiple comparisons corrections).

Next, we assessed the cellular component of anti-IN immune response. IFN-γ/IL-2 Fluorospot tests demonstrated that splenocytes of mice DNA immunized with IN variants exhibited potent IFN-γ, IL-2, and IFN-γ/IL-2 response to stimulation with peptides representing epitope-rich regions of integrase (Supplementary Figure S1; Supplementary Table S1) (*p* < 0.01 compared to empty vector and naïve mice, Mann–Whitney test; Figure 9a–c). All IN variants induced an equally high response (>10^3^ IFN-γ secreting cells per mln) to in vitro stimulation with peptides representing a promiscuous CD8+ T cell IN epitope at aa 209–239 (MIN219 and IN209 peptides, Supplementary Table S1) (Figure 9). Mice DNA-immunized with IN_a also developed weak predominantly IL-2 response against subdominant CD4+ epitopes at aa 79–98 and 169–196 (peptides MIN79, IN169, and MIN169; Supplementary Table S1) (Figure 9). No IFN-γ or IL-2 response was detected against epitope clusters at aa 8–33, 36–50, 137–161, 187–213, and 242–272 (Supplementary Figure S1, Supplementary Table S1). 

Introduction of inactivation mutation D64V led to a decrease in IFN-γ, IL-2, and IFN-γ/IL-2 production, with shrinkage of the spectrum of recognized epitopes (compare mice DNA immunized with IN_a to mice immunized with IN_in; Figure 9a–c). Introduction of RAL resistance mutations had an inverse effect, partially recovering recognition of murine epitopes at aa 79–98 and 169–190 (Figure 9a–c). Inactivated RAL resistant IN variants were equally immunogenic (*p* > 0.1; Figure 9a–c).

**Figure 10:**
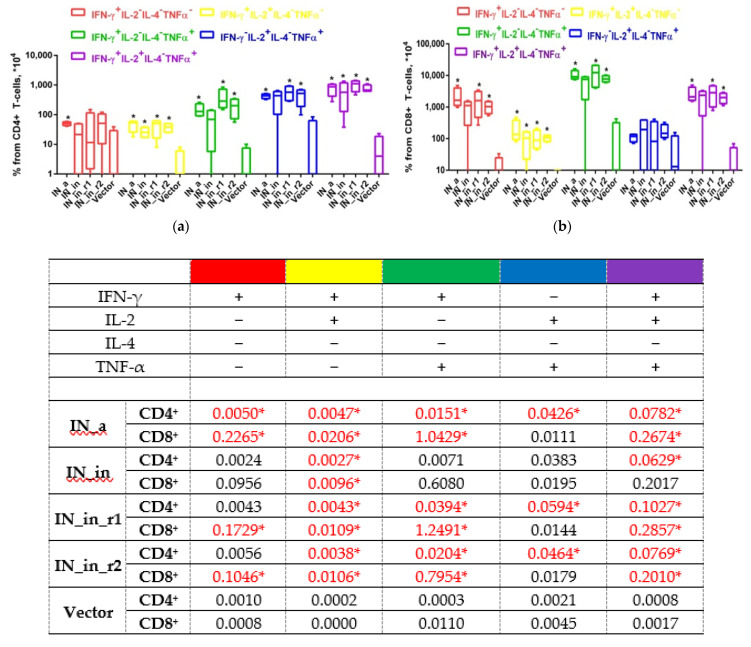

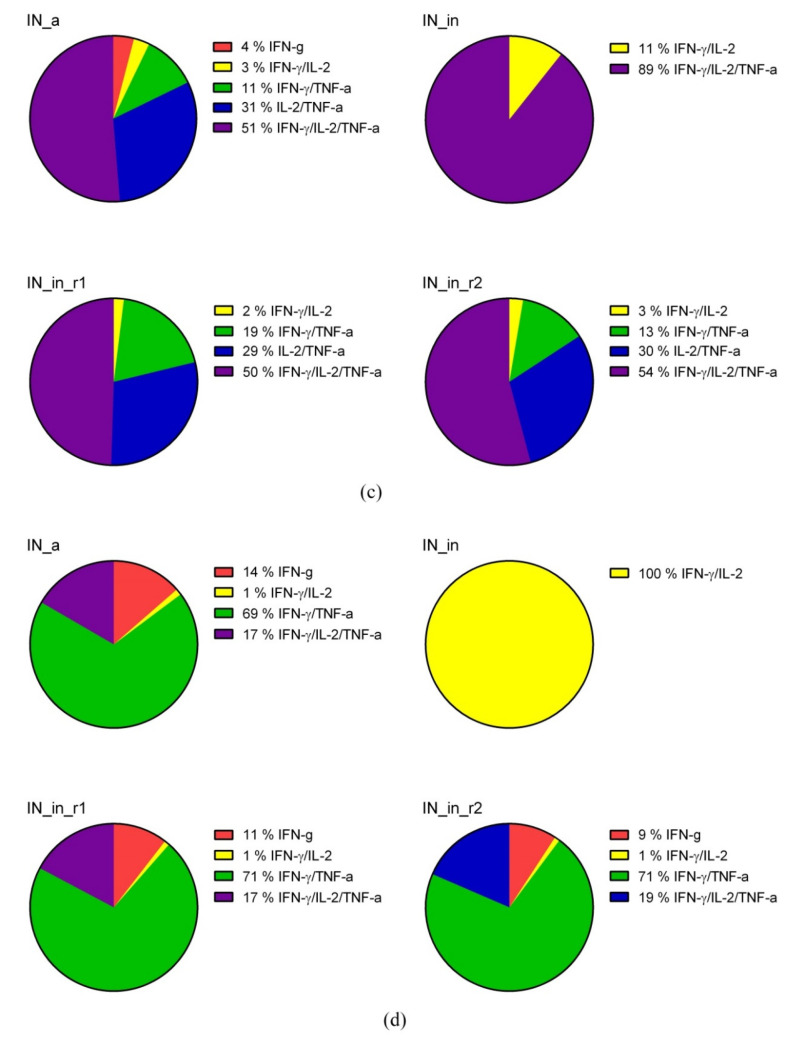
DNA immunization of mice with HIV-1 integrase variants induces multicytokine response of CD4+ and CD8+ T cells. CD4+ (**a**,**c**) and CD8+ (**b**,**d**) T cell response of mice DNA immunized by IN_a, IN_in, IN_in_r1, and IN_in_r2 (Table 1, Series I) to in vitro stimulation with MIN pool of IN-derived peptides (Supplementary Table S1) detected by flow cytometry with ICCS assessment presented as percent of total CD4+ (**a**), total CD8+ T cells (**b**), and pie diagrams illustrating distribution of populations of CD4+ (**c**) and CD8+ (**d**) T cells responding to stimulation by producing different cytokines, in %. Percent of the total responsive CD4+ or CD8+ T-cells was determined for every population of responding cells after subtraction of % of cells stimulated by medium alone; data represent two independent experiment runs, each done in duplicate. (**a**,**b**) Whisker bars represent median, quartiles, and minimum and maximum values of % of cells registered in each group; median values used to build whisker bars are presented in the table beneath the panels. (**c**,**d**) To build pie diagrams, all MIN-specific T cell responses by individual mice in each group were summed, taken for 100% and response of each type was represented as % of the total. *—*p* < 0,05 in (**a**,**b**), comparison of IN DNA- and vector-immunized mice, Kruskal–Wallis and Mann–Whitney tests; respective values in the data table are also indicated with * and given in red. Mouse groups were similar with respect to the levels of unspecific reactivity and cell viability demonstrating equal % of cytokine-producing T cells mock-stimulated with medium, or stimulated with mitogen ConA.

Further, we determined the reactive T cell populations, assessing production of IFN-γ, IL-2, IL-4, TNF-α (Series I), or IFN-γ, IL-2, perforin, and Granzyme B (GrB) (Series II, Table 1) by CD4+ and CD8+ T cells using flow cytometry with intracellular cytokine staining (ICCS). All IN variants induced strong IN-specific cellular response manifested by production of IFN-γ and of IFN-γ/IL-2/TNF-α, IFN-γ/IL-2, IFN-γ/TNF-α, and IL-2/TNF-α combinations, but no secretion of IL-4 (Figure 10). The response by both CD8+ and CD4+ T cells significantly exceeded the background levels registered in vector-immunized mice (*p* < 0.05, Figure 10). IN-specific response involved mainly CD8+ T cells reaching 1% of CD8+ T cell population; the levels of reactive CD4+ T cells were 10 times lower (≤ 0.1% for any given profile of cytokine secretion; Figure 10a,b). Of note, up to 1% of CD8+ and 0.1% of CD4+ T cells responded by triple production of IFN-γ/IL-2/TNF-α (Figure 10a,b). Confirming the data obtained in Fluorospot assays, the least immunogenic was IN_in, deficient in the capacity to induce multiple cytokine response by CD8+ and CD4+ T cells (Figure 10b,c). Other IN variants were equally immunogenic (*p* > 0.1; Figure 10). Thus, the loss of immunogenicity due to D64V mutation was restored by introduction of RAL resistance mutation patterns.

**Figure 11:**
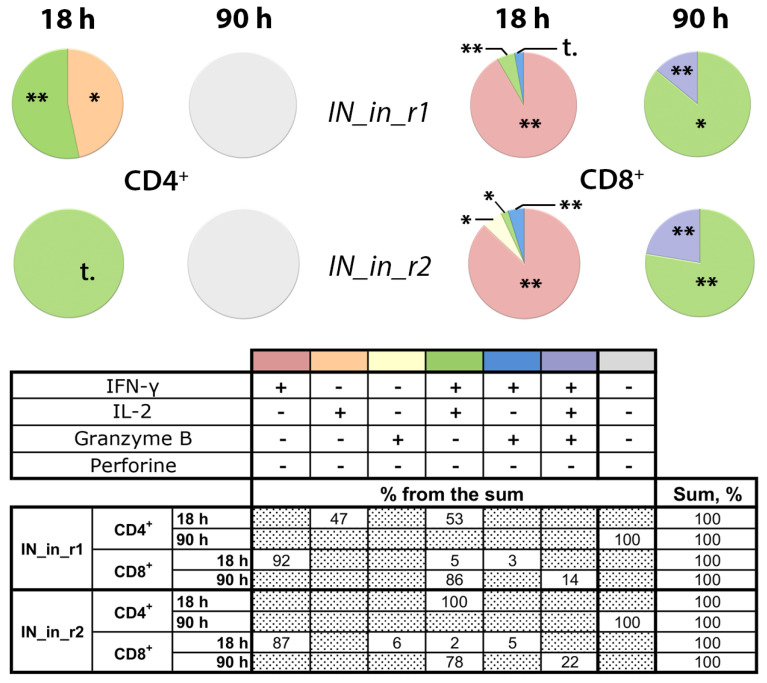
DNA immunization of BALB/c mice with consensus HIV-1 clade A integrase with mutations conferring resistance to raltegravir induces potent lytic response of CD8+ T-cells. Mice were DNA immunized with IN_in_r1 or IN_in_r2, or empty vector (Table 1, Series II). Splenocytes of mice were in vitro stimulated with MIN peptide pool (Supplementary Table S1) for 18 or 90 h (see Materials and Methods). Percent of CD4+ and CD8+ T cells producing IFN-γ, IL-2, Granzyme B, and perforin was assessed by flow cytometry with ICCS. Media values were subtracted, and data entries for two independent runs, each done in duplicate, were pooled, and statistical analysis was performed of the differences between values exhibited by MIN stimulated splenocytes of IN and of vector-immunized mice. Thereafter, for each T cell population, we subtracted mean percent of responding cells in vector-immunized mice from respective mean values exhibited by IN DNA-immunized mice. Resulting values are presented in circular diagrams as shares of the total responsive CD4+ or CD8+ T cell populations, altogether constituting 100%, and are presented in the table below the graph; the colors of the sectors of the pie diagram match the color used for this combination of cytokines in the table; “+” and “−“ in the head of the table below the diagrams; dotted boxes correspond to no detectable cells of a given type. Groups did not differ in cell viability, levels of unspecific reactivity, and response to stimulation with mitogens ConA and PMA. **—*p* < 0.01, *—*p* < 0.05, statistically significant difference, and “t”, tendency for difference with vector-immunized mice (*p* < 0.1) using Mann–Whitney test after the Holm/Hochberg multiple comparisons correction.

We performed additional analysis of T cell reactivity in mice DNA immunized with IN_in_r1 and IN_in_r2 (Table 1, Series II) to follow the kinetics of CD4+ and CD8+ T-cell response. Splenocytes of mice collected 18 days after DNA immunization were stimulated with MIN peptide pool (Supplementary Table S1) for 18 or 90 h, and assessed for a response to specific stimulation by production of IFN-γ, IL-2, perforin, and Granzyme B (GrB), to characterize lytic cellular immune response. After 18 h of stimulation, CD4+ T cells of mice in both groups responded mainly by production of IFN-γ/IL-2 (IN_in_r1, IN_in_r2) or IL-2 alone (IN_in_r1; Figure 11). No responsive CD4+ T cells were detected after 90 h stimulation (Figure 11). Transient CD4+ T cell response preceded lytic IFN-γ/IL-2 and IFN-γ/IL-2/GrB response of CD8+ T cells. Early reactive CD8+ T cells responding by mono-production of IFN-γ were detected already at 18 h. After 90 h stimulation, ≥80% CD8+ T cells in both groups produced IFN-γ/IL-2 (Figure 11). A proportion of CD8+ T cells produced GrB: after 90 h incubation, the proportion of IFN-γ/IL-2/GrB+ positive CD8+ T cells in IN_in_r1-immunized mice reached 14%, and, in IN_in_r2-immunized mice, 22% (*p* > 0,1; Figure 11), manifesting potent IN-specific lytic activity [76]. 

Thus, we have shown that DNA immunization with IN variants induces moderate antibody response, moderate response of CD4+ T cells, and potent response of CD8+ T cells with lytic potential. The weakest immunogen in terms of antibody response was IN_in_r2 (Figure 8), and, in terms of cellular immune response registered as IFN-γ and IL-2 production by Fluorospot and multicytokine production by CD4+ and CD8+ T cells, IN_in, whereas active IN_a and inactivated RAL-resistant IN variants demonstrated similarly high cellular immunogenicity (Figure 9, Figure 10 and Figure 11). 

### 3.9. Composite Profiles of In Vitro and In Vivo Properties of IN DNA Immunogens and Their Comparison and Correlation of Immunogenicity with In Vitro Properties of Integrase Variants

To understand the correlates of immunogenic performance of IN variants, we decided to present their properties as “composite profiles” uniting the results of in vitro and in vivo immunogenicity tests, and compare if these profiles of IN variants differ. The in vitro properties of IN variants included the level of expression, half-life, percent of degradation by proteasome and by lysosome, and levels of ROS. Comparative analysis of the groups revealed a tendency to difference mostly due to the difference between IN_in_r1 compared to IN_in_r2 (assigned the lowest and the highest ranks, respectively) (*p* = 0.092) However, pair-wise analysis revealed no difference between the cumulative in vitro properties of IN variants (Friedman ANOVA with Kendall coefficient of concordance followed by Sign test; Supplementary Table S4-1). 

The in vivo properties of IN variants included % of CD4+ and of CD8+ T cells responding to stimulation with MIN peptide pool by production of one, two, and three cytokines, sum of reactive CD4+ and CD8+ T cell populations, % of reactive CD4+ and CD8+ T cells of the total CD4+ and CD8+ T cell population, ratio of reactive CD4+ and CD8+ T cells, and titers of antibodies against IN variant used for immunization, IN_A and IN_B variants (mean values; from immunization Series I, Table 1). As in the case of in vitro properties, comparative analysis of the groups revealed a tendency to difference mostly due to the difference between IN_in_r1 compared to IN_in_r2, receiving the lowest and the highest ranks, respectively (*p* = 0.1 by Friedman ANOVA with Kendall coefficient of concordance; Supplementary Table S4-2). Pair-wise analysis pinpointed that difference between the immunogenic performance of IN variants was due to a difference between IN_in_r1 and IN_in_r2, that approached the level of significance (*p* = 0.059 Sign test; Supplementary Table S4-2). 

Finally, we compared IN variants by composite profiles uniting in vitro and in vivo properties (Supplementary Table S4-3) to find significant difference between IN_a, IN_in, IN_in_r1, and IN_in_r2 (*p* = 0.045 by Friedman ANOVA) due to the difference between the properties of IN_in_r1 and IN_in_r2 (*p* = 0.02, Sign test) (Supplementary Table S4-3). Thus, despite similarity in single tests, IN_in_r1 and IN_in_r2 were significantly different in the composite profiles uniting their in vitro and in vivo properties.

**Figure 12:**
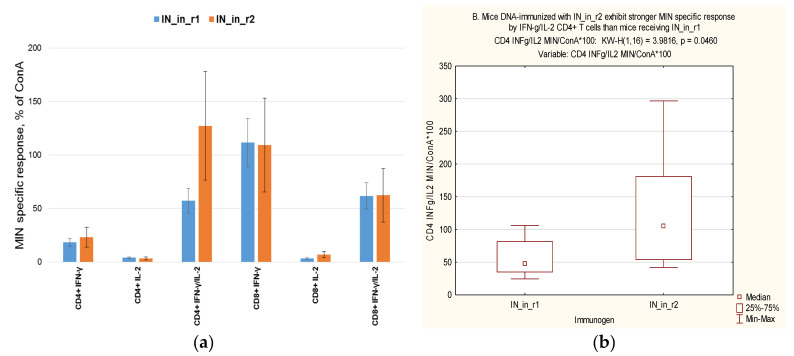
DNA immunogens encoding inactivated HIV-1 integrases with two patterns of resistance to raltegravir (IN_in_r1, IN_in_r2) differ in the ability to induce integrase-specific IFN-γ/IL-2 response by CD4+ T cells. IFN-γ, IL-2, IFN-γ/IL-2 response of CD4+ and CD8+ T cells of mice DNA-immunized with IN_in_r1 or IN_in_r2 (Series I, II; Table 1) were presented as % of response of respective cell populations to ConA (**a**); statistically significant difference in proportion of MIN-specific IFN-γ/IL-2 secreting CD4+ T cells (**b**). Data represent average values for mice DNA-immunized with IN_in_r1 (Series I/group 3, *n* = 4; Series II/group 1, *n* = 6) and IN_in_r2 (Series I/group 4, *n* = 4; Series II/group 2, *n* = 6) ±SD.

We attempted to dissect the inter-relation between in vitro properties of IN variants and their immunogenicity. No correlations were found between in vitro properties of IN variants and levels of anti-IN antibodies (Spearman rank order test; R values <0.2, *p* values >0.1). To perform the analysis for cellular immune response, we united data on cellular immune response found to be specific in immunization Series I and II (Table 1), namely IFN-γ/IL-2 response of CD4+ and CD8+ T cells to stimulation with MIN peptide pool, and to mitogen ConA. Series I and II were independent immunization experiments done at different time points, with T cell response assessed by flow cytometry with ICCS using different panels of antibodies, and, therefore, could not be directly pooled to dissect the in vitro correlates of immune response. To normalize the data, we used the levels of cytokine production in response to ConA. The latter characterizes T cell viability and functionality, and it had the same magnitude in Series I and Series II (Series I, valid *n* = 16, Series II, valid *n* = 12; adjusted Z for CD4+ T cells = -0.91, for CD8+ T cells = 1,53; *p*-value for both >0.1). This enabled us to present IFN-γ and IL-2 response of CD4+ and CD8+ T cells to MIN as percent of respective response to ConA (Figure 12a). Significant difference between IN_in_r1 and IN_in_r2 was revealed in IFN-γ/IL-2 CD4+ T cell response to MIN, which, in IN_in_r1 DNA-immunized mice, was lower than in mice receiving IN_in_r2, while no difference was observed between other responses of CD4+ or CD8+ T cells (Figure 12a,b).

Layouts of Series I and II, focusing in different T cell populations, did not allow to relate the difference in representation of IN-specific IFN-γ/IL-2 CD4+ T cells to changes in the proportions of other MIN-specific T cell populations. However, analysis of the data obtained in Series I gave some indications. Whereas the proportions of MIN-specific IFN-γ/TNF-α secreting CD4+ and CD8+ cells were correlated (R = 0.54, *p* < 0.05), inverse correlations were found between the proportions of IFN-γ/TNF-α and IFN-γ/IL-2 secreting CD4+ (R= -0.509) and CD8+ T cells (R= -0.797) (Spearman rank order correlation test, *p* < 0.05; Supplementary Table S5). Altogether, this pointed towards distinct patterns of CD4+ T cell response, one with predominance of IFN-γ/IL-2, the other, of IFN-γ/TNF-α production. The latter indicated that higher percent of IFN-γ/IL-2 secreting CD4+ T cells induced by IN_in_r2 compared to IN_in_r1 may reflect an opposite situation for IFN-γ/TNF-α CD4+ T cells, i.e., higher levels of IFN-γ/TNF-α CD4+ T cells in response to IN_in_r1 than in response to IN_in_r2. Indeed, that was the case, % of IFN-γ/TNF-α CD4+ T cells in response to IN_in_r1 was two times higher than in response to IN_in_r2 (Figure 10a), although the difference was not significant due to too few observations.

Further, we correlated in vitro properties of IN variants (Figure 2, Figure 3 and Figure 4) to cellular responses to IN variants from fused experiments of Series I and II (data from single experiments of Series I or II were insufficient to run correlation tests). The only parameter of cellular immune response influenced by the in vitro properties of integrases was IFN-γ/IL-2 response of CD4+ T cells, and it tended to increase IFN-γ response of CD8+ T cells (Table 2). Protein stability had an inverse effect, as increased stability of IN variants reduced, and preferential proteasomal processing increased IFN-γ/IL-2 response by CD4+ T cells. Production of ROS had negative effect on IFN-γ/IL-2 response of CD4+ T cells. IFN-γ/IL-2 response of CD4+ and CD8+ T cells did not depend on the level of IN expression (Table 2). No correlations were found between in vitro properties of IN variants and antibody response against IN used in immunization and homologous IN variants (Table 2). Thus, out of the panels of IN-based DNA immunogens designed in this study, IN_in_r1 and IN_in_r2, representing two patterns of primary and secondary mutations to RAL, were the most different with respect to in vitro and in vivo immunogenic properties. The main difference lays in their capacity to induce IFN-γ/IL-2 response by CD4+ T cells, which was correlated to the degree of their proteasomal processing (inversely correlated to stability) and also inversely correlated to their capacity to induce ROS. The latter corroborated our earlier findings for HIV-1 RT [72] and HCV nucleocapsid (core) protein [77].

### 3.10. DNA Immunization With RAL-Resistant IN Variants, Safety Issues

We further tested potential toxicity of enzymatically inactivated IN gene variants. For this, BALB/c mice were DNA-immunized with three times higher dose of the plasmids than in Series I, II (60 µg compared to 20 µg per animal) using optimized DNA immunization protocol [16] (Table 1, Series III). Mice received either IN_in, or an equimolar mix of plasmids encoding RAL resistant IN_in_r1 and IN_in_r2, or empty vector, or PBS, introduced into three sites as a prime followed three weeks later by an identical boost. All injections were followed by electroporation using CUY21EditII electroporator (BEX, Japan) equipped with fork/plate electrodes under strict control of the electric current. We assessed the effect of DNA immunization on the general health condition, body mass, parameters of complete blood count, biochemical blood tests, bone marrow composition and mass of lymphoid organs (Supplementary Tables S7–S12, respectively). General condition of the animals was physiologically normal throughout the study period. Animals in all groups actively consumed feed and water. Social behavior, pose, coordination, breathing, defecation, urination, reaction to stimuli, muscle tone, the condition of the coat, mucous membranes, eyes, nasal cavity, oral cavity and other parameters corresponded to the physiological norm (Supplementary Table S7). IN DNA immunization did not lead to any differences in the body mass of the animals (Supplementary Table S8).

**Figure 13:**
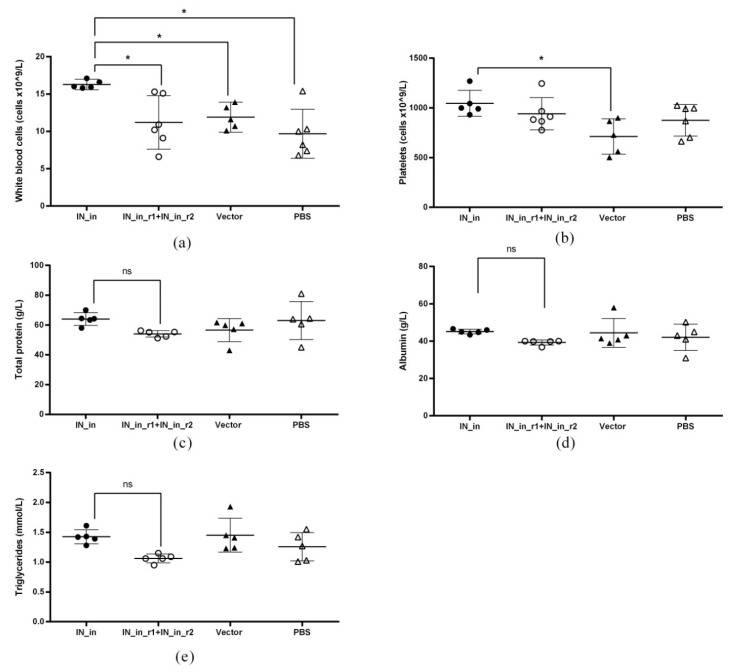
Parameters of biochemical analysis and complete blood counts of mice DNA-immunized with inactivated consensus HIV-1 integrase of clade A (IN_in) and a mixture of drug resistant inactivated IN variants IN_in_r1 and IN_in_r2. Scheme of the experiment is presented in Table I; see Series III. Panels represent individual values of immunized and control mice receiving vector DNA or PBS: white blood cell counts (**a**), platelet counts (**b**), and content of the total protein (**c**), albumin (**d**), and triglycerides (**e**), and average group values with SD. *—*p* < 0.05 in Kruskal–Wallis and Mann–Whitney tests with Bonferroni correction.

Complete blood count and biochemical blood analysis were performed on day 13 after the boost. White blood cells and platelet counts in mice DNA-immunized with IN_in were significantly higher than in mice receiving IN_in_r1 + IN_in_r2 and control vector and PBS (Figure 13a,b).

Counts of other populations of blood cells and relative proportion of granulocyte and lymphocyte populations did not differ (Supplementary Table S9). Biochemical blood analysis revealed that mice DNA immunized with IN_in had significantly higher levels of total protein, albumin and triglycerides than mice DNA immunized with IN_in_r1 + IN_in_r2 mixture (Figure 13c,d,e; *p* < 0,05; Supplementary Table S10); respective parameters in other groups did not differ (Figure 13c,d,e; Supplementary Table S10; *p* > 0.05 in pair-wise comparisons). There was no difference between the groups in the composition of bone marrow, nor mass of the lymph nodes, thymus, and liver (Supplementary Tables S11 and S12; *p* > 0.05).

Thus, all parameters of mice receiving DNA encoding RAL-resistant IN variants were undistinguishable from the controls. Increased levels of white blood cell and platelet counts, total protein, albumin, and triglycerides in IN_in group pointed at adverse effect(s) of IN_in DNA administration. The results of immunogenicity and toxicity assessments motivated exclusion of IN_in DNA immunogen from further studies.

### 3.11. DNA Immunization with RAL-Resistant Inactivated IN Hinders In Vivo Growth of IN Expressing Tumor Cells

**Figure 14:**
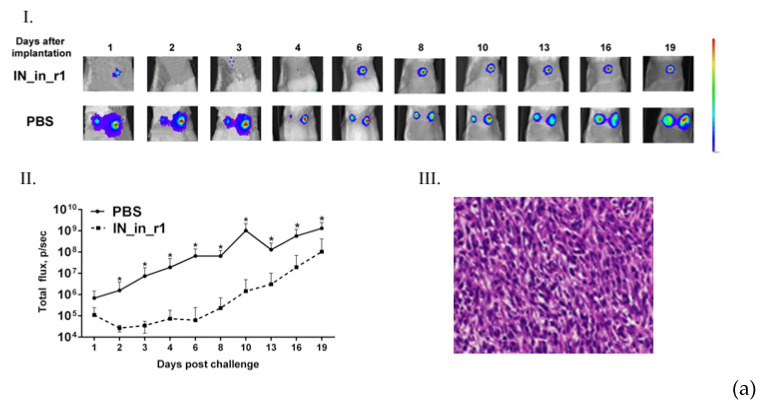

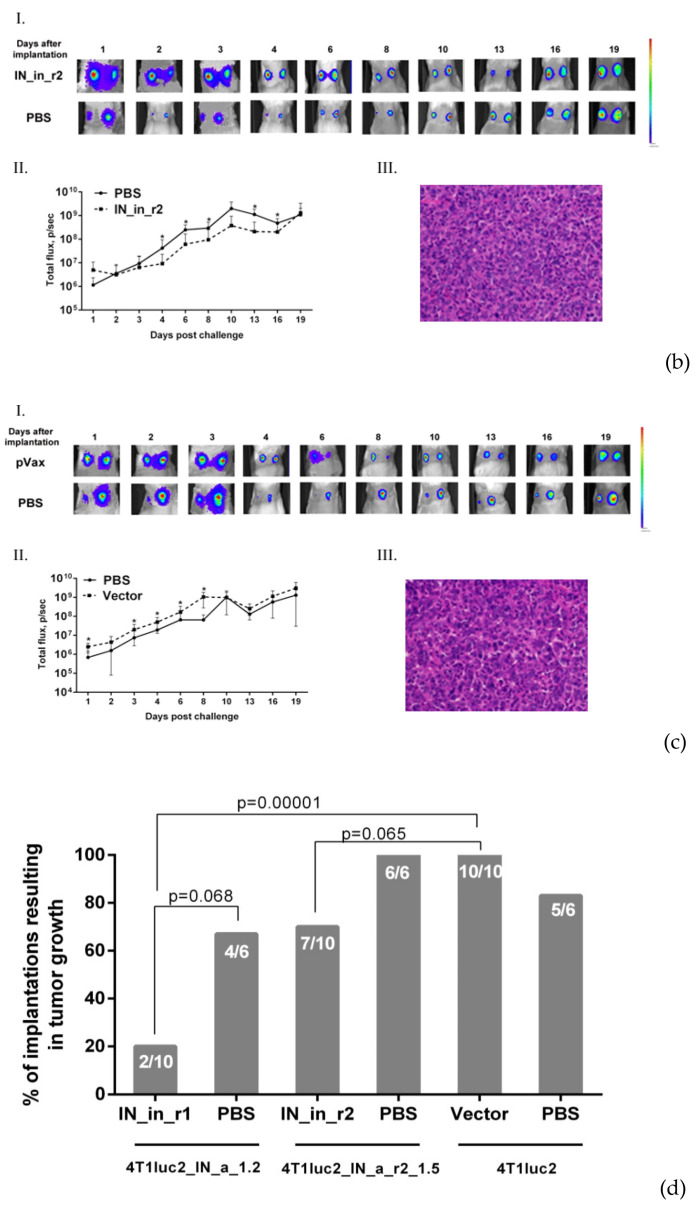

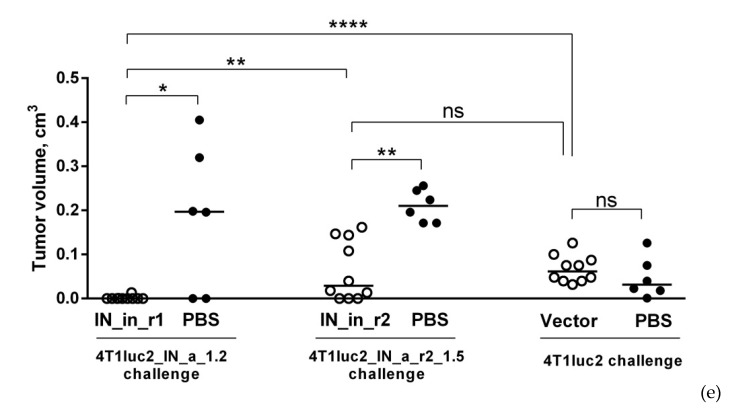
DNA immunization with RAL resistant IN variants hinders growth of IN expressing tumor cells. Growth of mammary gland adenocarcinoma 4T1luc2 cells expressing IN_a (4T1luc2_IN_a_1.2) (**a**) or RAL resistant IN_a_r2 (4T1luc2_IN_a_r2_1.5) (**b**) or Parental cells (4T1luc2) (**c**) in mice DNA immunized with IN_in_r1 (**a**), IN_in_r2 (**b**), or empty vector (**c**) compared to PBS-immunized animals challenged with the same cells (**a**–**c**); % of implantations resulting in tumor growth, the white figures over the bars represent the number of developing tumors to the total number of implantations with tumorigenic cells (**d**); tumor volumes, line bars represent the median values, cm^3^ (**e**). In vivo BLI monitoring was done on days 1, 2, 4, 6, 8, 10, 13, 16, 19 post-implantation. Panel I in (**a**–**c**) show the growth of representative tumors, and color scale to the right demonstrates BLI intensity. Panel II illustrates growth of tumors assessed as the average total photon flux (*p*/sec) from the sites of implantation on days 1–19 post-cell lines implantation, mean ± SD. Panel III shows representative images of H&E stained sections of FFPE blocks of the solid tumors formed in mice immunized with IN_in_r1 and challenged with 4T1luc2_IN_a_1.2 (**a**), immunized with IN_in_r2 and challenged with 4T1luc2_IN_a_r2_1.5 (**b**), immunized with pVax1 and challenged with 4T1luc2 (**c**); magnification × 400 (Leica DM500, Wetzlar, Germany). Statistically significant differences not indicated on the graph: IN_in_r1 versus pVax1-immunized mice—*p* < 0.001 for total flux on days 1, 3, 4, 6, 8, 10, 13, 16, 19, *p* < 0.01 on day 2; IN_in_r2 versus pVax1-immunized mice—*p* < 0.05 on days 3, 4, 6, 8, 10, 16, *p* = 0.06 on day 19; IN_in_r1 versus IN_in_r2-immunized mice—*p* < 0.05 (on days 4, 13), *p* < 0.01 (days 3, 10), *p* < 0.001 (days 1, 2, 6, 8). Numerical comparisons are done with *t*-test, *p*-values are indicated on the graph. *—*p* < 0.05, **—*p* < 0.01, ****—*p* < 0.0001 by Kruskal–Wallis and Mann–Whitney tests.

Finally, we launched an experiment to determine the protective potential of immune response against IN. For this end, we subjected mice DNA immunized with IN_in_r1 and IN_in_r2 and with vector pVax1 to challenge with murine mammary gland adenocarcinoma 4T1luc2 cells expressing IN_a (4T1luc2_IN_a_1.2) or IN_a_r2 (4T1luc2_IN_a_r2_1.5) or parental cells 4T1luc2 (Table 1, Series IV). Choice of the clone for challenge was based on a match between IN variants used as immunogens versus those expressed by tumor cells: 100% match (except for inactivation mutation) in the case of IN_in_r2 immunization and IN_a_r2 challenge, and the best possible match for IN_in_r1 immunization and IN_a challenge, as we could not generate 4T1luc2 cells expressing IN_a_r1. IN_in_r1 and IN_a differ in six, and IN_in_r1 and IN_a_r2, in nine, amino acid residues. Tumor growth was monitored by in vivo BLI and morphometrically (Figure 14a–c, panels I, II and panel III, respectively). Mice DNA-immunized with IN variants demonstrated lower levels of total photon flux from tumors compared to mock/PBS-immunized mice receiving same tumor cells (compare data in panels I and II in Figure 14a–c). The phenomenon was specifically pronounced for mice DNA-immunized with IN_in_r1 (Figure 14a, panels I, II). Mice immunized with empty vector demonstrated elevated rate of tumor growth compared to IN DNA-immunized mice and mice receiving PBS (Figure 14c; panels I, II; *p*-values are indicated in the figure caption).

IN_in_r1-immunized mice were also statistically significant different, and IN_in_r2 tended to differ from the vector-immunized controls in the number of “successful” tumor cell implantations (Figure 14d). While all vector- and PBS-immunized mice developed tumors, 1/5 of IN_in_r1 and 3/5 of IN_in_r2 DNA-immunized mice were totally protected from tumor growth (Figure 14d). BLI data was supported by the morphometric measurements demonstrating significantly reduced tumor volumes in mice DNA-immunized with IN_in_r1 and IN_in_r2 compared to vector- or PBS-immunized controls (Figure 14e). Tumors that developed in mice immunized with IN_in_r1 and challenged with 4T1luc2_IN_a_1.2, were >15 times smaller, and, in IN_in_r2-immunized mice challenged with 4T1luc2_IN_a_r2_1.5, 4–10 times smaller than tumors formed in the PBS-immunized animals (Figure 14e). In addition, tumors in IN_in_r1 DNA-immunized mice were significantly smaller than in mice receiving IN_in_r2 (Figure 14e; *p* < 0.01, F-test, and Mann–Whitney tests). Overall, DNA immunizations with RAL resistant IN variants provided resistance to growth of IN-expressing tumors, more pronounced for IN_in_r1 based on both the results of BLI and tumor morphometric assays. Thus, DNA immunization with IN_in_r1 was able to significantly suppress growth of 4T1luc2 cells expressing heterologous IN variant, while IN_in_r2 showed only limited suppression of growth of tumor cells expressing homologous IN (except for inactivation mutation) (Figure 14).

At the experimental endpoint, mice were euthanized, and tumors were excised, fixed, paraffin embedded (FFPE), sectioned, H & E stained, and examined by light microscopy. The histological evaluation of tumors formed in IN_in_r1 and IN_in_r2 DNA-immunized mice revealed no significant differences from the tumors formed by these cell lines in PBS-immunized (panel III of Figure 14a–c) or naive animals (Figure 5).

### 3.12. DNA Immunization with RAL-Resistant Inactivated IN Variants Suppresses Metastatic Activity of IN-Expressing Tumor Cells

Further, we evaluated if DNA immunization with IN variants could affect the metastatic potential of IN expressing tumor cells compared to that in control mice. For this, lungs, livers, and spleens of mice collected at the experimental end-point were subjected to ex vivo imaging to assess total number of organ-infiltrating tumor cells [28]. Ex vivo BLI assessment of lung, liver, and spleen samples, in all groups, registered photon flux exceeding the level ascribed to one Luc-expressing 4T1 cell only in the case of lungs (http://www.caliperls.com/assets/014/7158.pdf) (accessed on 20 February 2015); Supplementary Figure S12). This observation corroborated our initial evaluation of the metastatic activity of IN expressing 4T1luc2 clones (Figure 6). Based on these observations, we concentrated on the assessment of metastatic activity in the lungs.

**Figure 15:**
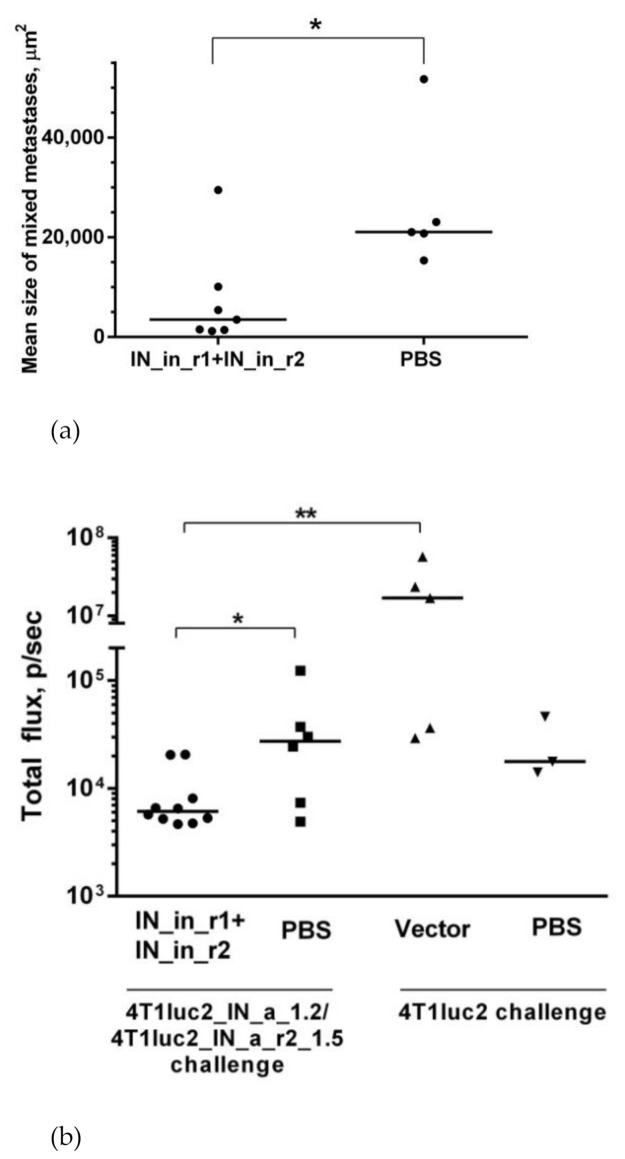
DNA immunization with RAL resistant IN variants suppresses metastatic activity of IN expressing murine adenocarcinoma cells. Assessment of infiltration of tumor cells into the lungs by ex vivo BLI, where line bars represent the median of total flux from the lungs of mice DNA-immunized with IN variants (*n* = 10) or mock-immunized with PBS (*n* = 6) and implanted with IN-expressing 4T1luc2 cells (**a**); mean size of the lung metastases, µm^2^, in mice DNA-immunized with IN variants or mock-immunized with PBS and implanted with 4T1luc2 cells expressing IN_a or IN_a_r2 (**b**). Number and size of metastases were calculated in 25 high power (400×) microscope fields of hematoxylin–eosin-stained slides by computer-assisted morphometry. Line bars represent median values. *—*p* < 0.05, **—*p* < 0.01, no *p* values indicate no statistically significant differences; Kruskal–Wallis, Mann–Whitney tests.

Photon flux from lungs of mice DNA immunized with IN_in_r1 challenged with IN_a expressing tumor cells, and DNA immunized with IN_in_r2 and challenged with IN_a_r2 expressing tumor cells, did not differ (*p* > 0.1), allowing a merge of two datasets. Pooled data was compared to the total flux from the lungs of mice immunized with IN_in_r1 and IN_in_r2 and challenged with 4T1luc2_IN_a_1.2 and 4T1luc2_IN_a_1.5, and immunized with pVax1 and challenged with 4T1luc2 cells (Figure 15a). The ex vivo total flux in lungs of IN-immunized mice was significantly lower than the total flux from lungs of both PBS-immunized (*p* < 0.05) or vector-immunized mice (*p* < 0.05) (Figure 15a).

Histological examination of the lungs of IN-immunized and control mice discovered single metastases composed of tumor and inflammatory cells with no qualitative or quantitative difference either from non-immunized mice implanted with 4T1luc2 parental cells or their IN expressing clones examined by us earlier (Figure 6b–j), or between the groups of IN_in_r1 and IN_in_r2-immunized mice. Thus, IN DNA immunization had no effect on either the number (low), nor morphology, of lung metastases. We assessed the number and the size of these mixed type metastases (immune infiltrates). Mice in all the groups had very few or no lung metastases (mean nn of metastases 1 ± 0.5 per mouse with no difference between the groups, *p* > 0.2). The size of metastases in IN DNA-immunized groups was smaller than that in the mock-immunized mice implanted with the same cells (*p* < 0.05, Figure 15b). Thus, IN DNA immunization hindered infiltration of IN expressing tumor cells into the lungs, and it reduced the size of infrequent lung metastases.

### 3.13. Suppression of In Vivo Growth of IN Expressing Tumor Cells by DNA Immunization with RAL-Resistant Inactivated IN Variants Correlates with Cellular Response Against IN Epitopes

**Figure 16:**
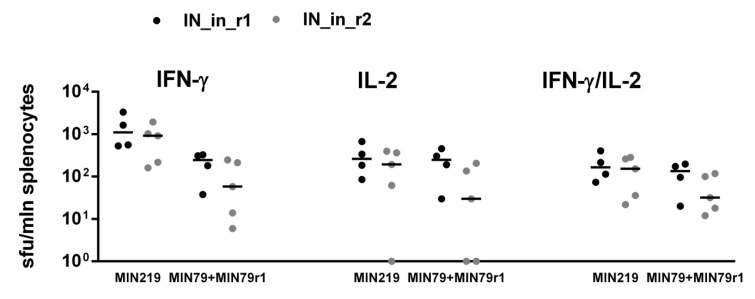
Splenocytes of mice DNA immunized with IN variants and implanted with murine adenocarcinoma cells expressing IN, demonstrate potent IFN-γ/IL-2 response against IN epitopes at aa 79–98 (MIN79 + MIN79r1) and aa 219–238 (MIN219). For the description of immunization scheme, see Figure 14. Twenty-one days after challenge splenocytes of mice were stimulated in vitro with individual IN-derived peptides (Supplementary Table S1) for 18 h, as described in Materials and Methods. In vitro secretion of IFN-γ, IL-2, and dual secretion of IFN-γ/IL-2 was measured as the number of signal-forming units (sfu) per mln splenocytes, done in duplicates. Line bars represent the median of sfu/mln splenocytes; no statistically significant differences were registered (*p* > 0.1; Mann–Whitney test).

Splenocytes of DNA-immunized and control mice implanted with IN-expressing and parental 4T1luc2 cells were assessed for the ability to secrete IFN-γ/IL-2 in response to stimulation with IN-derived peptides using Fluorospot. No IN-specific response was detected in vector- or PBS-immunized mice, indicating that implantation of IN-expressing tumors as such did not generate any IN-specific immunity. Both protected, partially protected, and non-protected IN-immunized mice exhibited IN-specific cellular response manifested by IFN-γ, IL-2, and dual IFN- γ/IL-2 production in response to in vitro stimulation of splenocytes by peptides representing immunodominant CD8+ (MIN219) and CD4+ T-cell epitope (MIN79 + MIN79r1 corresponding to IN carrying the mutations of drug resistance). Cellular response induced by IN_in_r1 and IN_in_r2 immunizations did not differ (*p* > 0.1, Figure 16). The magnitude of cellular response to IN peptides evaluated in IN-immunized mice was inversely correlated with the tumor size and total photon flux from the tumors at the experimental endpoint (Supplementary Table S13). Specifically, strong inverse correlation was found between total photon flux from tumor areas and cellular response to peptide representing immunodominant epitope of IN at aa 219–238 (R ≥ −0.933; Supplementary Table S13), although Fluorospot did not allow to identify the nature of immunoreactive cells.

## 4. Discussion

We synthesized an expression optimized gene encoding consensus integrase (IN) of HIV-1 clade A, and using it as a platform, two drug resistant variants with alternative patterns of resistance to raltegravir combining primary and early occurring secondary/adaptive mutations L74M/E92Q/V151I/N155H/G163R (IN_r1_in) or E138K/G140S/Q148K (IN_r2_in). Consensus IN was significantly more active than viral integrases (such as IN of HIV-1 HXB2) in the reactions of DNA cleavage and integration. Enzymatic tests confirmed that both integrases are insensitive to the inhibitors of strand transfer, as was expected [25], therefore representing ideal components of DNA vaccine against drug resistant HIV-1 [9]. Integrase is genotoxic due to its capacity to integrate foreign DNA into host genome [68,69]. Introduction of RAL resistance mutations led to considerable, but not total, loss of both LTR processing and joining activities [25], requesting further inactivation. Hence, we modified both enzymes introducing inactivating mutation D64V [78], and proved the loss of 3′-processing and strand transfer activities, making IN variants safe to use as DNA immunogens.

Level of expression and route of intracellular processing are critical for DNA vaccine efficiency [79,80,81]. We evaluated these properties of IN variants in a series of in vitro tests. All IN variants, parental, drug resistant, active, and inactivated, were expressed in eukaryotic cells at a similar level of 0.4–0.6 pg per cell independently of cell origin. Further, we investigated if inactivation and/or drug resistance mutations have any effect on the proteolytic processing. IN of HIV-1 subtype B has been shown to be a substrate of N-end rule pathway, degraded by the proteasome [82,83,84,85]. The half-life of IN of HIV-1 subtype B with Met instead of Phe on its N-terminus increases from 15 min to more than 3 h [82,86]. Although a recent study questioned N-end rule pathway-mediated degradation during HIV-1 infection [87], our in vitro data confirms the original findings. The half-life of the consensus IN of HIV-1 subtype A with Met-Gly dipeptide preceding Phe on the enzyme N-terminus exceeds 3 h. None of the mutations made IN instable; on the contrary, incorporation of drug resistance mutations in both IN variants led to a loss of sensitivity to proteasomal inhibitors. Interestingly, they have also led to an enhanced IN degradation by the lysosome. Stabilizing effect of pan-lysosomal inhibitor chloroquine was equal to the combined effect of the inhibitors of individual classes of lysosomal proteases, indicating that lysosomal degradation of IN variants was carried out by a cocktail of lysosomal proteases of different specificities. Interestingly, incorporation of the inactivation mutation D64V partially reversed both effects, restoring IN sensitivity to proteasome and inhibiting its sensitivity to lysosome. As a result, both inactivated RAL resistant IN variants were moderately stable, with half-life exceeding 3 h, with a balanced degradation by proteasome and lysosome, which allowed us to predict their good immunogenic performance along cellular and humoral axis of immune response.

Oxidative stress is a potent modulator of the immune response [72,77]. We have shown that oxidative stress induced by HCV nucleocapsid (core) protein interferes with its immunogenicity in DNA immunization [77]. Several HIV proteins, such as gp120, Tat, Nef, and reverse transcriptase (RT), were shown to induce oxidative stress [15,72,88,89]. Capacity to induce oxidative stress and oxidative stress response shapes the immunogenic performance of HIV-1 RT in DNA immunization, reducing cellular and increasing humoral immunogenicity [17,72]. The capacity of HIV-1 IN to induce oxidative stress was not known. Here, we have shown that expression of all IN variants with and without drug resistance or inactivating mutations, induces oxidative stress. Reactive oxygen species (ROS) production in IN expressing cells increased 4–6 times compared to control (vector transfected) cells. The levels of ROS were 1.5–2-fold lower than that induced by HIV-1 RT, and could be expected to cause less interference with IN immunogenicity in DNA immunization. The panel of IN variants with varying levels of ROS induction also allowed us to determine/quantify the dependence of immunogenicity on the capacity of the immunogen to induce ROS.

It is important to keep in mind the interrelation of proteasomal processing and ROS. ROS affects proteasome (dysfunction of 26S proteasome) and proteasomal processing [90]. When oxidized protein is processed by the proteasome, it does not follow the same route as in the non-oxidative environment. Oxidized proteins are processed by specific proteasomal variants that process only oxidized proteins, while “normal” proteasomal processing in the context of oxidative stress is impaired [91,92]. Oxidative stress or rather oxidative environment can be also causative, or consecutive, to protein aggregation, leading to increased stability of the involved/affected proteins [93]. Here, we observed that inactivated IN_a (IN_in), causing the highest level of ROS, had the longest half-life, and, overall, the levels of ROS were directly correlated to the protein half-life and inversely correlated to the degree of its processing by proteasome (data in Figure 3a–d, Figure 4; R values of 0.76 and -0.89, *p* < 0.05, Spearman rank test). Hence, one cannot expect processing of oxidized proteins to lead to the same level of T cell response, and to the same products, as in the case of their non-oxidized counterparts. Modifications may result in a loss of tolerance to self-antigens [94], as well as loss of recognition of microbial antigens [95]. The effect could be more pronounced for CD4+ T cells; they are longer and, hence, statistically more prone to oxidative modifications and generation of neo-epitopes.

Altogether, we collected a set of in vitro characteristics of IN variants, to try using them as predictors of immunogenic performance in DNA immunization, with positive input of proteasomal processing, and negative input of oxidative stress. Comparison of the integral in vitro properties (Friedman ANOVA test with Kendall coefficient of concordance) revealed a difference, specifically between RAL resistant inactivated INs (IN_in_r1 versus IN_in_r2), although it did not reach the level of significance (*p* = 0.09, Friedman ANOVA test with Kendall coefficient of concordance). DNA immunization with these variants opened a possibility to see if this difference would translate into a difference in immunogenic performance.

More and more data are accumulated on the direct carcinogenic effects of HIV-1 proteins, such as Nef, gp120, Tat, p17, and RT, associated with their ability to induce oxidative stress [89]. We have shown that expression of HIV-1 RT promotes tumor growth and enhances metastatic activity of tumor cells [28]. Since IN is found here to induce production of ROS, albeit at a lower level than RT, it was important to show that it does not result in tumorigenic effects (apart from the effects caused by genotoxicity). We launched experiments to test this by obtaining clones of murine adenocarcinoma 4T1luc2 cells and making them express enzymatically active integrases (IN_a and IN_a_r2) by lentiviral transduction. Expression of integrases was toxic to the transduced cells. Toxic effect was more pronounced for IN_a (we could obtain transduced clones only at MOI 1) and less pronounced for IN_a_r2 (expressing subclones were obtained at MOI 1 and 5), the effect explainable by difference in the integrase activity, grossly compromised for IN_a_r2 due to introduction of RAL resistance mutations. Implanted into syngeneic BALB/c mice, 4T1luc2 clones expressing IN_a and IN_a_r2 produced tumors undistinguishable from that formed by the parental 4T1luc2 cells, with similar rates of tumor growth and similar metastatic activity. From this, we could conclude that, contrary to observations made for HIV-1 RT, expression of INs, even if enzymatically active, is not associated with an enhanced carcinogenicity, which supports their safety as DNA immunogens.

Lastly, we tested if introduction of DNA-encoding inactivated IN variants causes immunotoxicity. Here, immunotoxicity refers to any adverse effect/change on the structure or function of the immune system, or on other systems as a result of immune system dysfunction. “Change” in an immune function or level of immunological mediator may not necessarily appear as an “adverse effect”, but can also be an immunostimulation. Caution must be exercised in such cases because a non-specific enhancement of the immune response, interpreted as a beneficial effect for an immunogen, may result in exaggerated immune reaction, and enhancement of specific, as well as unspecific, immune response and excess inflammation, resulting sensitization and/or autoimmune disease. DNA immunization with RAL resistant IN variants caused no adverse effects. Analysis of other parameters, including general condition of the animals, body mass, parameters of complete blood count, biochemical blood tests, bone marrow composition and mass of lymphoid organs, revealed no significant differences between the groups that received DNA immunization with HIV integrase sequences compared to empty vector and the control group, indicating favorable safety profile of inactivated RAL resistant integrases as DNA immunogens. On the contrary, DNA immunization with inactivated non-resistant IN resulted in increase in WBC and platelet counts in clinical, and total protein, albumin and triglyceride content in biochemical blood tests. Increase in WBC and platelet counts in rodents are associated with inflammation [96]. Increases in total protein also indicate inflammation, specifically hyperglobulinemia [97]. High albumin levels may be caused by acute infections, burns, trauma and stress. Albumin, as a protein made by the liver, serves as markers for its biosynthetic activity and function, and an increase in albumin levels indicates alterations of liver function [98]. Increased levels of triglycerides could also result from hepatic disorders [99]. Thus, our data demonstrated the immunotoxicity of inactivated non-mutated IN, which caused alterations in murine blood associated with inflammation, and safety of DNA immunogens based on inactivated RAL resistant IN variants.

Next, we evaluated the immunogenicity of IN variants in DNA immunization; their genes were introduced into mice by single or repeated intradermal injections followed by electroporation. Immune response was assessed by IFN-γ/IL-2. In Fluorospot assays, splenocytes of mice DNA immunized with IN variants strongly recognized peptides MIN219 and IN209 representing at aa 209–239 of IN. Aa 209–239 contain CD8+ T cell epitopes recognized in both mice and man, indicating its promiscuity [19,36,38]. Mice DNA-immunized with IN_a also weakly recognized peptides MIN79, IN169, and MIN169, representing aa 79–98 and 169–196 of IN. Other IN peptides representing known IN epitopes were not recognized. This indicated that mice DNA-immunized with IN variants had a strong hierarchy of T cell responses with dominant T cell epitope cluster localized at aa 209–239 and subdominant T cell epitopes aa to aa 79–98 and 169–196. Introduction of inactivation mutation D64V led to a decrease in peptide-specific cytokine production, and shrinkage of the spectrum of recognized epitopes compared to that induced by enzymatically active IN variant, although the epitopes above were not directly affected by the mutation. This fell in line with the observation of a decreased proteasomal processing of IN_in (in favor of lysosomal degradation). Interestingly, introduction of RAL resistance mutations had an inverse effect, partially restoring the levels of peptide-specific cytokine production, and recovering recognition of the epitopes at aa 79–98 and 169–190. As a result, parental IN_a and RAL resistant IN variants with D64V mutation were equally potent in inducing IFN-γ/IL-2 production by splenocytes of mice stimulated with IN-derived peptides.

Further, we determined which T cell populations were involved in the cytokine response by assessing production of IFN-γ, IL-2, IL-4, TNF-α (Series I immunization) or IFN-γ, IL-2, and Granzyme B (GrB) (Series II immunization) by CD4+ and CD8+ T cells using flow cytometry with ICCS. All IN variants induced multicytokine response in the form of production of combinations of IFN-γ/IL-2, IFN-γ/TNF-α, IL-2/TNF-α, IFN-γ/GrB, IFN-γ/IL-2/TNF-α, and IFN-γ/IL-2/GrB, mainly by CD8+ T cells. Reactive CD8+ T cells constituted up to 1% of CD8+ T cell population; the levels of the reactive CD4+ T cells were 10 times lower. Kinetic studies performed for RAL resistant IN variants demonstrated that the immune response ex vivo was initiated by IL-2 or IFN-γ/IL-2 production by CD4+ T cells, with further expansion of IFN-γ/GrB and IFN-γ/IL-2/GrB CD8+ T cells. Such cytokine secretion profiles are characteristic to the effector CD8+ T cells and multi-functional CD4+ T cells [76]. Detection of GrB production by IN-specific CD8+ T cells is specifically interesting. One of the major functions of CD8+ T cells specific to HCMV is the lysis of virus-infected cells through the secretion of perforin and GrB. GrB mediates cleavage of HCMV infected cells and activation of proapoptotic target proteins, such as caspases and Bid [100]. Memory cells have high transcript levels of GrB, but express it only upon activation, while HCMV-specific CD8+ T cells carry high levels of preformed GrB molecules stored in intracellular granules that allows them to quickly respond and efficiently lyse infected cells [76,101]. We observed GrB production by CD8+ T cells early after their stimulation (< 1day), expanding 4–5-fold by day 3, which altogether characterized strong lytic potential of anti-IN immune response. Multi-functional CD4+ T cells are equally or even more important. They are critical for formation of memory B cells, the generation and differentiation of CD8+ cytotoxic CTLs that control viral replication, and mobilization of CTLs to the peripheral sites of infection, as well as direct cytotoxic activity [102,103,104].

The magnitude of CD4+ or CD8+ T cell multicytokine response serves as a reliable correlate of vaccine-induced protection [102], also in HIV-1 vaccine development [105]. Generation of such response by DNA immunization with IN variants manifests their high immunogenicity and potential for efficient elimination of HIV-1 infected cells. We could not test this until lately, but we had an indirect instrument for the assessment of the capacity of IN-specific immune response to clear IN-expressing cells. This was assessed by co-immunizing mice with the plasmids encoding INs mixed with the plasmid encoding Luc reporter. We have earlier shown that introduction of a mixture of potent DNA immunogen with plasmid encoding Luc results in immune clearance of immunogen/Luc co-expressing cells registrable as an accelerated loss of total photon flux from the sites of immunization, as the loss of bioluminescence correlates with the induction of IFN-γ/IL-2 response against the immunogen [75]. Here, introduction of each of IN variants mixed with Luc DNA resulted in a complete loss of bioluminescent signal from the sites of immunization, pointing at the lytic capacity of anti-IN immune response with lytic potential predictable from the magnitude of CD4+ or CD8+ T cell multicytokine response.

IN variants were also immunogenic on the B cell level; they stimulated moderate IN-specific antibody response effective in cross-recognition of different variants of IN proteins of subtype A, including active and inactive, wild type and drug resistant variants. IN_in_r2 was the least immunogenic on the B cell level, inducing lower cumulative levels of anti-IN antibodies (Supplementary Figure S11e).

In flow cytometry with ICCS, as in the Fluorospot assays, IN_in was the least immunogenic, being deficient in the capacity to induce multiple cytokine response by CD8+ and CD4+ T cells. The loss of immunogenicity due to D64V mutation was restored by introduction of RAL resistance mutation patterns; RAL resistant IN variants were equally immunogenic. This observation, repeated in two immunization series and uncovered by different tests, stimulated us to look for the correlations between immunogenicity and in vitro IN properties (the level of expression, proteasomal degradation and induction of ROS). The in vitro properties did not correlate with either CD8+ T cell, or antibody response, but were statistically significantly correlated to IN immunogenicity for CD4+ T cells. Protein stability and high levels of ROS decreased, and preferential proteasomal processing increased IN-specific IFN-γ/IL-2 response of CD4+ T cells (Table 2). This falls in line with earlier studies demonstrating ROS-mediated alterations of protein processing, as well as altered CD4+ T cell recognition of the oxidized proteins [106,107]. At the same time, contrary to earlier observations [16], anti-IN immune response, either cellular or antibody, did not depend on the level of IN immunogen expression. This could be due to a narrow range of variation of IN expression levels (0.4–0.6 pg/cell), an impact on immunogenicity may emerge if the difference reaches one or two orders in magnitude [16].

Further, we created immunogenic profiles of integrases combining all quantitative characteristics of their immunogenic performance. Comparative analysis of the immunogens revealed a tendency for difference, due to differential performance of IN_in_r1 and IN_in_r2 (*p* = 0.059, Supplementary Table S4-2), which was not evident in single immune tests, but was revealed by their combination. Interestingly, complex assessment of in vitro and in vivo properties in IN variants, made this difference statistically significant (*p* = 0.045 for group comparison on IN_a, IN_in, IN_in_r1, and IN_in_r2; *p* = 0.02 for pair-wise comparison of IN_in_r1 and IN_in_r2 by Sign test). The key question arising in this context was whether these differences would translate into differential performance in the tests of vaccine efficacy.

Finally, we tested the protective potential of two RAL resistant inactivated IN variants (IN_a was excluded due to genotoxic enzymatic activity, and IN_in, due to immunotoxicity) in a murine tumor model of HIV-1 infection based on the 4T1luc2 cells expressing IN variants. Mice were DNA immunized with IN_in_r1 and IN_in_r2 using optimized DNA immunization protocol [16] and subjected to ectopic subcutaneous challenge with murine mammary gland adenocarcinoma 4T1luc2 cells expressing IN_a or IN_a_r2. Murine cells expressing enzymatically active INs served as a model of challenge with HIV-1 infected cells. Optimal sites and doses of 4T1luc2 cells to be implanted were defined, allowing reproducible linear growth of tumors over the first week, and humane end-point, after three weeks of the observation [28,60]. Of note, IN_in_r2 DNA-immunized mice received a challenge with 4T1luc2 cells expressing IN identical to the immunogen with the exception of D64V mutation. We could not obtain 4T1luc2 cells expressing IN_in_r1; hence, IN_in_r1 DNA-immunized mice were challenged with 4T1luc2 cells expressing IN_a, different from the immunogen in six amino acid residues, of which one, E92Q, was localized in the T cell epitope at aa 79–98 recognized by IN-immunized mice (Figure 9).

Among IN_in_r1-immunized mice, 2/5 had tumors 15-times smaller than in mock-immunized animals, and 3/5 were tumor-free. Among IN_in_r2-immunized mice, 1/5 had tumors comparable to mock-immunized mice, 3/5 showed 3–5 reduction in tumor size, and one mouse was protected. Protected and partially protected mice exhibited weak IN-specific cellular response manifested by IFN-γ, IL-2, and dual IFN-γ/IL-2 production in response to in vitro stimulation of their splenocytes by peptides representing T cell epitopes of IN at aa 79–98 and 219–238. IFN-γ/IL-2 response inversely correlated to the size of the tumors. IN-expressing 4T1luc2 cells had low tumorigenic activity, forming no metastases in spleens, nor in livers, and few metastases in lungs. IN DNA-immunized mice had single metastases in lungs, with no difference from the control PBS-immunized animals. However, the size of metastases was significantly smaller than in controls. Thus, DNA immunization with IN_in_r1 conferred 60% protection against tumor growth, and, in 40%, significantly hindered growth of integrase-expressing tumors, with resistance to tumor growth depending on the cellular immune response against epitopes of integrase. DNA immunization with IN_in_r2 was less effective. This demonstrated the potential of DNA-immunization with drug-resistant INs to confer partial protection/resistance to establishment of IN expressing tumors, despite the difference between INs immunogen and IN expressed by the tumor cells.

This data also answered the question of whether a difference in in vitro properties of proteins encoded by DNA immunogens and differences in their immunogenic performance, detectable only after their cumulative assessment, can result in differences in their performance in the challenge tests. Yes, IN variants with different cumulative sets of in vitro and in vivo properties offered different levels of resistance against challenge with IN expressing tumor cells, with one acting even against a deviant IN, and the other, inefficient against challenge with nearly identical IN variant. Flow cytometry with ICCS could not detect significant difference in the cellular immunogenicity of RAL resistant INs, but observationally, IN-specific IFN-γ/TNF-α response in the case of IN_in_r1 involved 1.67% and, in the case of IN_in_r2, 1.09%, and IFN-γ/IL-2 response 0.4% and 0.3% of the total T cell population, respectively (see data in Figure 10), pointing at a stronger overall cellular immunogenicity of IN_in_r1. It could not be proven statistically in single tests but was revealed by the analysis of cumulative properties of the proteins (Supplementary Table S4). Reduced size of IN-expressing tumors correlated with the numbers of IN-specific IFN-γ/IL-2 secreting cells in Fluorospot test. Since ≥1% of the reactive T cells are CTLs (CD4+ T cells constitute ≤0.1%), the major input into the immune control was made by CD8+ T cells.

At the same time, by normalizing the results of flow cytometry assays in Series I and II, we revealed significant difference between IN_in_r1 and IN_in_r2 in the levels of IN-specific IFN-γ/IL-2 producing CD4+ T cells (higher in the case of IN_in_r2). Since IN_in_r2 mice exhibited low level of resistance to tumor growth, despite a match between IN immunogen and IN expressed by tumor cells (except for D64V mutation), this type of response was apparently unfavorable. Of note, the percent of IN-specific IFN-γ/IL-2 CD4+ T cells was inversely correlated with the percent of CD4+ T cells expressing IFN-γ/TNF-α. Thus, lower level of IFN-γ/IL-2 producing CD4+ T cells of IN_in_r1-immunized mice may have indicated higher levels of CD4+ T cells producing IFN-γ/TNF-α, with the latter, or both events, contributing to a better protection of these mice against tumor challenge. TNF-α/TNFR1–mediated signals on APCs and TNF-α/TNFR2 signals on T cells are critically required for effective priming, proliferation, and recruitment of tumor-specific T cells in mice. In the absence of TNF-α signaling, tumor immune surveillance is severely abrogated [108]. Importantly, TNF-α production occurs as early as 1 h following T cell activation and precedes IL-2 and IFN-γ release; furthermore, TNF-α release and activation of TNFR2 affects the efficiency of the subsequent T cell proliferation and cytokine production [108], supporting the findings of inverse correlation between the populations of IFN-γ/TNF-α and IFN-γ/IL-2 producing CD4+ T cells. Furthermore, IFN-γ/IL-2 profile of cytokine production (without TNF-α) could be ascribed to regulatory T cells that suppress the anti-tumor functions of CD4 +, CD8 +, and NK cells, leading to an absence of effective anti-tumor immune response [109]. IN_in_r1 induces a preferable profile of immune response rich in IFN-γ/TNF-α/IL-2 and IFN-γ/IL-2/GrB producing CD8+ T cells, and IFN-γ/TNF-α and IFN-γ/IL-2/TNF-α producing CD4+ T cells, allowing better control of tumor cell challenge.

Altogether, we demonstrated that DNA-immunization with drug resistant INs can confer resistance to establishment of tumors expressing homologous IN variants which does not rely on 100% identity between the immunogen and antigen present in the challenge, but rather on the profile of immune response against IN as tumor associated antigen, with dominance of the specific multicytokine expressing CD4+ and CD8+ T cells. Tumor cell lines expressing HIV-1 antigens analogous to the ones used in this study can serve as a powerful tool to test the efficacy of therapeutic HIV vaccine candidates compensating for the absence of easily accessible HIV-1 challenge models running in mice.

## Figures and Tables

**Table 1:** Schemes of immunization of mice with IN-encoding plasmids.

Groups	Mice (*n*)	DNA Immunogen	Electroporation Technique	Injections Per Mouse per Time Point	Total Dose of Immunogen per Mouse per Time Point	Challenge with IN Expressing Tumorigenic Cells
SERIES I—SINGLE IMMUNIZATION						
I	4	IN_a *	DermaVax, multi-needle electrodes [65]	2	20 µg	No
II	4	IN_in *		2	20 µg	
III	4	IN_in_r1 *		2	20 µg	
IV	4	IN_in_r2 *		2	20 µg	
V	4	Empty vector (pVax1) *		2	20 µg	
SERIES II—SINGLE IMMUNIZATION						
I	6	IN_in_r1 *	DermaVax, multi-needle electrodes [65]	2	20 µg	No
II	6	IN_in_r2 *		2	20 µg	
III	4	Empty vector (pVax1) *		2	20 µg	
SERIES III—PRIME-BOOST IMMUNIZATION **						
I	6	IN_in	CUY21EditII, fork/plate electrode [16]	3	60 µg	No
II	6	IN_in_r1 + IN_in_r2 (1:1, *v*/*v*) ***		3	60 µg	
III	6	Empty vector (pVax1)		3	60 µg	
IV	6	PBS		3	60 µg	
SERIES IV—PRIME-BOOST IMMUNIZATION						
Ia	5	IN_in_r1	CUY21EditII, fork/plate electrode [16]	4	40 µg	4T1luc2_IN_a_1.2
Ib	3	PBS		4	0 µg	4T1luc2_IN_a_1.2
IIa	5	IN_in_r2		4	40 µg	4T1luc2_IN_a_r2_1.5
IIb	3	PBS		4	0 µg	4T1luc2_IN_a_r2_1.5
IIIa	5	Empty vector (pVax1)		4	40 µg	4T1luc2
IIIb	3	PBS		4	0 µg	4T1luc2

* DNA immunogens were mixed with plasmid encoding luciferase reporter (pVaxLuc) 1:1 (*w*/*w*); ** DNA prime, followed 3 weeks later by identical DNA boost; *** Ratio of IN_in_r1+ IN_in_r2 encoding plasmids 1:1 *v*/*v* corresponds to equimolar proportion due to nearly identical molecular mass of the plasmids.

**Table 2:** In vitro correlates of potency of T cell response in mice DNA immunized with variants of consensus HIV-1 integrase. Table depicts correlation coefficients in the Spearman rank order correlation test between average values of each in vitro and in vivo parameter characteristic to IN variants. Statistically significant correlation coefficients (*p* < 0.05) are given in red, tendency to correlate, in brown (*p* < 0.1).

Proportion of T Cells Reacting to MIN Stimulation by Cytokine Secretion, % to ConA	Expression, pg/Cell (Data in Figure 2)	Half-Life, h (Data in Figure 3a)	% Proteasomal Degradation (Data in Figure 3b)	ROS, Relative Units (Data in Figure 4)
CD4^+^ IFN-γ	0.117359	−0.085143	0.289618	−0.305397
CD4^+^ IL-2 *	−0.184019	0.060563	−0.118215	−0.036105
CD4^+^ IFN-γ/IL-2 *	−0.063069	−**0.427204**	**0.536682**	−**0.449813**
CD8^+^ IFN-γ *	0.147779	−0.216828	**0.351055**	−0.218764
CD8^+^ IL-2 *	−0.287745	−0.147626	−0.017515	−0.027523
CD8^+^ IFN-γ/IL-2 *	0.151065	−0.088770	0.319262	−0.316147
IgG against IN immunogen **	−0.229193	−0.101328	−0.092884	0.101328
IgG against IN_a **	0.299564	−0.123632	0.143061	0.200818
IgG against IN_B **	−0.021122	0.203095	−0.220968	0.108859

* Specific to IN DNA-immunized compared to vector-immunized mice (*p* < 0.05) (Figure 10 and Figure 11); ** Specific to IN DNA-immunized compared to vector-immunized mice (*p* < 0.05) (Figure 8).

## Data Availability

Not applicable.

## Supplementary Materials

The following are available online at https://www.mdpi.com/article/10.3390/microorganisms9061219/s1, Figure S1: Epitopic map of HIV-1 integrase, Figure S2: The scheme of lentiviral vector used for cloning of IN variants, Figure S3: 4T1luc2 cells after transduction with lentiviral particles encoding enzymatically active consensus HIV-1 clade A integrase, Figure S4: Detection of the genomic inserts of sequences encoding HIV-1 clade A FSU_A integrase under the control of PGK promoter in the daughter clones of 4T1luc2 cells, Figure S5: Determination of the catalytic activity of consensus HIV-1 FSU_A integrase (IN_a) and its variants with two patterns of mutations to raltegravir L74M/E92Q/V151I/N155H/G163R (IN_a_r1) and E138K/G140S/Q148K (IN_a_r2), Figure S6: Level of expression of variants of integrases of HIV-1 clade A FSU_A strain in human (HEK293T) and murine (NIH3T3) cells, pg/cell, Figure S7: The kinetics of integrase degradation determined by cycloheximide chase, Figure S8: Effect of the proteasome and lysosome inhibitors on accumulation of IN, Figure S9: The effect of a non-specific lysosomal inhibitor chloroquine is correlated to the effect of the combination of protease-specific inhibitors leupeptin, aprotinin, pepstatin, and E-64, Figure S10: The results of PCR of genomic DNA of tumors produced by 4T1luc2 and their IN-expressing derivatives grown after ectopic implantation of BALB/c mice, Figure S11: DNA immunization with the IN gene variants induces cross-reactive IN-specific IgGs, Figure S12: Infiltration of tumor cells into organs of mice DNA immunized with IN variants challenged with 4T1luc2 cells derivatives expressing IN (4T1luc2_IN_a_1.2 and 4T1luc2_IN_a_r2_1.5) compared to mock-immunized mice challenged with the same cell lines, Table S1: Peptides and peptide pools used in in vitro T-cell stimulation tests, Table S2: Synthetic oligonucleotide duplexes used to assess enzymatic activities of HIV-1 integrase, Table S3: Primers used for generation of lentiviral vectors and confirmation of genomic IN inserts, Table S4: Comparison of in vitro and in vivo properties of IN-based DNA immunogens, Table S5: Correlations of the proportions of IFN-γ/IL-2 and IFN-γ/TNF-α secreting CD4+ and CD8+ T cells specific to integrase (MIN pool) in mice DNA-immunized with IN gene variants, Table S6: Expression of IN variants in tumors formed by implantation of 4Tlluc2_IN_a_1.2 and 4T1luc2_IN_a_r2_1.5 cells into BALB/c mice, Table S7: Animal welfare chart for the parameters assessed daily and weekly, Table S8: Body mass assessment in mice receiving plasmid encoding inactivated integrase (IN_in), a mixture of plasmids encoding drug resistant inactivated integrases IN_in_r1 and IN_in_r2 (1:1, *v*/*v*), empty vector pVax1, or PBS, followed by electroporation, Table S9: Complete blood counts of mice receiving plasmid encoding inactivated integrase (IN_in), a mixture of drug resistant inactivated integrases IN_in_r1 and IN_in_r2 (1:1, *v*/*v*), empty vector pVax1, or PBS, followed by electroporation, Table S10: Biochemical blood analysis of mice receiving plasmid encoding inactivated integrase (IN_in), a mixture of drug resistant inactivated integrases IN_in_r1 and IN_in_r2 (1:1, *v*/*v*), empty vector pVax1, or PBS, followed by electroporation, Table S11: Bone marrow composition of mice receiving plasmid encoding inactivated integrase (IN_in), a mixture of drug resistant inactivated integrases IN_in_r1 and IN_in_r2 (1:1, *v*/*v*), empty vector pVax1, or PBS, followed by electroporation, Table S12: Organ mass of mice receiving plasmid encoding inactivated integrase (IN_in), a mixture of drug resistant inactivated integrases IN_in_r1 and IN_in_r2 (1:1, *v*/*v*), empty vector pVax1, or PBS, followed by electroporation, Table S13: Inverse correlation of IFN-γ/IL-2 secretion by splenocytes in response to stimulation with IN peptides to mean tumor volume (cm3) and total photon flux from tumor area (*p*/sec) of mice immunized with IN gene variants and challenged with tumorigenic cell lines stably expressing IN.
