# Supplementary material for: Cellular Immune Response Induced by DNA Immunization of Mice with Drug Resistant Integrases of HIV-1 Clade A Offers Partial Protection against Growth and Metastatic Activity of Integrase-Expressing Adenocarcinoma Cells
Source: Microorganisms. 2021 Jun 4;9(6):1219. doi: 10.3390/microorganisms9061219 (PMC8226624; doi:10.3390/microorganisms9061219)
Supplement: Supplementary file 1 [file microorganisms-09-01219-s001.zip › microorganisms-1223555-supplementary.pdf]

SUPPLEMENTARY FILES

```
                                    10        20        30        40        50        60        70        80        90       100
                                     |         |         |         |         |         |         |         |         |         |
IN_a                       FLEGIDKAQEEHEKYHSNWKAMASDFNLPPIVAKEIVASCDKCQLKGEAMHGQVDCSPGIWQLDCTHLEGKVIIVAVHVASGYIEAEVIPAETGQETAYF
IN_i                       FLEGIDKAQEEHEKYHSNWKAMASDFNLPPIVAKEIVASCDKCQLKGEAMHGQVDCSPGIWQLVCTHLEGKVIIVAVHVASGYIEAEVIPAETGQETAYF
IN_iR1                     FLEGIDKAQEEHEKYHSNWKAMASDFNLPPIVAKEIVASCDKCQLKGEAMHGQVDCSPGIWQLVCTHLEGKVIMVAVHVASGYIEAEVIPAQTGQETAYF
IN_iR2                     FLEGIDKAQEEHEKYHSNWKAMASDFNLPPIVAKEIVASCDKCQLKGEAMHGQVDCSPGIWQLVCTHLEGKVIIVAVHVASGYIEAEVIPAETGQETAYF
hINb40_807_817             -------------------------------------------------------------------------------------------ETGQETAYF
zmaIN_848_856Bjo            -------------------------------------------------------------------------------SGFIEAEVI-------
mINh2k_786_793Sid          ----------------------------------------------GEAMHGQV-------
mamuIN_826_835Bjo          ----------------------------------------------------------GTWQMDCTHL--------------------------
INb71_66_74                -----------------------------------------------------------------THLEGKIIL--------------------------
maIN_835_844Sid            -------------------------------------------------------------------LEGKIIIVAV-----------------------
hINb54_792_800             -----------------------------------------------------------------------------HVASGYIEA---------
hINhlaDR_70_84             ---------------------------------------------------------------------GKIILVAVHVASGYI----------------
hIN_786_808                -----------------------------------------------------------------------IILVAVHVASGY-EAEVIPAETG------
mINcd4_789_798                     ----------------------------------------------------------------------VASGYIEAEVIPAETGQETA--
maIN_840_854Dzu            -------------------------------------------------------------------------IVAVHVASGFIEAEV------------
hINb40_799_808             -----------------------------------------------------------------------------------IEAEVIPAETG------
hINb18i40_851_856          -----------------------------------------------------------------------------------IEAEVIPQET-------
hINb18i40_799_806          -----------------------------------------------------------------------------------IEAEVIPA---------
INb42i51_28_36              --------------------------LPPIVAKEI------------------------------------------------------------
hINmhc2_16_30              ---------------HSNWRAMASDFNLPP----------------------------------------------------------------------
hINdrb1i5_1174_1178W       ---------------HSNWRAMASDFNLPP----------------------------------------------------------------------
hINa2i68_1169_1178Al       ---------------------MASDFNLPPV---------------------------------------------------------------------
hINa02_1167_1175Alt        -------------------RAMASDFNL------------------------------------------------------------------------
INb72_135_143              -------IQQEFGIPY------------------------------------------------------------------------------------
hINa2pre_7                 ------KAQEEHEKY-------------------------------------------------------------------------------------
mamuIN776_786Sid           --------QEEHDKYHSNV---------------------------------------------------------------------------------
hIN_98-114Lub             --------------------------------------------------------------------------------------------------AYF

Prim.cons.                 FLEGIDKAQEEHEKYHSNWKAMASDFNLPPIVAKEIVASCDKCQLKGEAMHGQVDCSPGIWQLVCTHLEGKVIIVAVHVASGYIEAEVIPAETGQETAYF
```

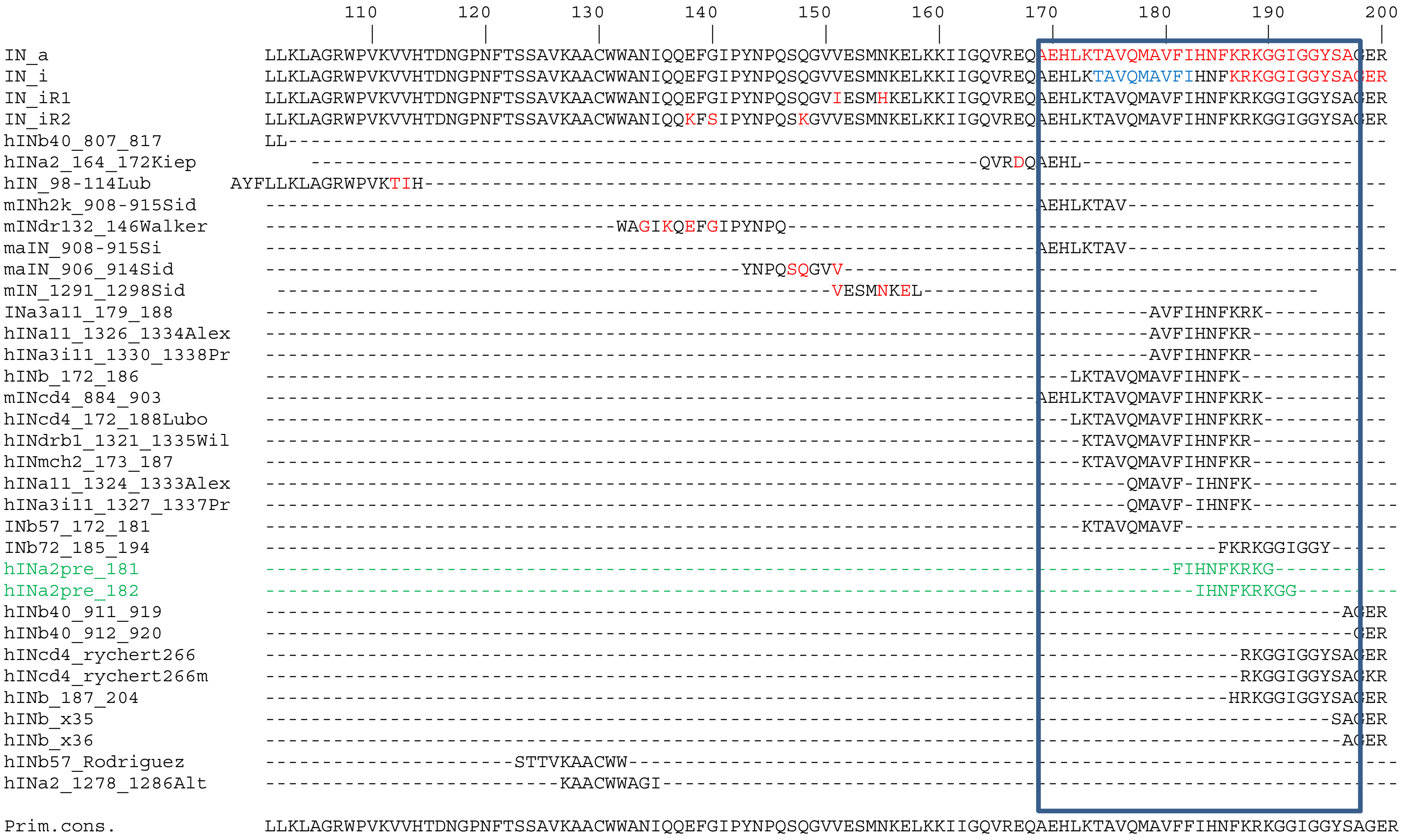
110 120 130 140 150 160 170 180 190 200
IN_a LLKLAGRWPVKVVHTDNGPNFTSSAVKAACWWANIQQEFGIPYNPQSQGVVESMNKELKKIIGQVREQAEHLKTAVQMAVFIHNFKRKGGIGGYSAGER
IN_i LLKLAGRWPVKVVHTDNGPNFTSSAVKAACWWANIQQEFGIPYNPQSQGVVESMNKELKKIIGQVREQAEHLKTAVQMAVFIHNFKRKGGIGGYSAGER
IN_iR1 LLKLAGRWPVKVVHTDNGPNFTSSAVKAACWWANIQQEFGIPYNPQSQGVIESMHKELKKIIGQVREQAEHLKTAVQMAVFIHNFKRKGGIGGYSAGER
IN_iR2 LLKLAGRWPVKVVHTDNGPNFTSSAVKAACWWANIQQKFSIPYNPQSKGVVESMNKELKKIIGQVREQAEHLKTAVQMAVFIHNFKRKGGIGGYSAGER
hINb40_807_817 LL
hINa2_164_172Kiep QVRDQAEHL
hIN_98-114Lub AYFLLKLAGRWPVKTIH
mINh2k_908-915Sid AEHLKTAV
mINdr132_146Walker WAGIKQEFGIPYNPQ
maIN_908-915Si AEHLKTAV
maIN_906_914Sid YNPQSQGVV
mIN_1291_1298Sid VESMNKEL
INa3a11_179_188 AVFIHNFKRK
hINa11_1326_1334Alex AVFIHNFKR
hINa3i11_1330_1338Pr AVFIHNFKR
hINb_172_186 LKTAVQMAVFIHNFK
mINcd4_884_903 AEHLKTAVQMAVFIHNFKRK
hINcd4_172_188Lubo LKTAVQMAVFIHNFKRK
hINdrb1_1321_1335Wil KTAVQMAVFIHNFKR
hINmch2_173_187 KTAVQMAVFIHNFKR
hINa11_1324_1333Alex QMAVF-IHNFK
hINa3i11_1327_1337Pr QMAVF-IHNFK
INb57_172_181 KTAVQMAVF
INb72_185_194 FKRKGGIGGY
hINa2pre_181 FIHNFKRKG
hINa2pre_182 IHNFKRKGG
hINb40_911_919 AGER
hINb40_912_920 GER
hINcd4_rychert266 RKGGIGGYSAGER
hINcd4_rychert266m RKGGIGGYSAGKR
hINb_187_204 HRKGGIGGYSAGER
hINb_x35 SAGER
hINb_x36 AGER
hINb57_Rodriguez STTVKAACWW
hINa2_1278_1286Alt KAACWWAGI
Prim.cons. LLKLAGRWPVKVVHTDNGPNFTSSAVKAACWWANIQQEFGIPYNPQSQGVVESMNKELKKIIGQVREQAEHLKTAVQMAVFFIHNFKRKGGIGGYSAGER

```
                                      210       220       230       240       250       260       270       280
                                        |         |         |         |         |         |         |         |
IN_a                        IIDIIATDIQTKELQKQIIKIQNFRVYYRDSRDPIWKGPAKLLWKGEGAVVIQDNNDIKVVPRRKAKIIRDYGKQMAGDDCVASRQDED
IN_i                        IIDIIATDIQTKELQKQIIKIQNFRVYYRDSRDPIWKGPAKLLWKGEGAVVIQDNNDIKVVPRRKAKIIRDYGKQMAGDDCVASRQDED
IN_iR1                      IIDIIATDIQTKELQKQIIKIQNFRVYYRDSRDPIWKGPAKLLWKGEGAVVIQDNNDIKVVPRRKAKIIRDYGKQMAGDDCVASRQDED
IN_iR2                      IIDIIATDIQTKELQKQIIKIQNFRVYYRDSRDPIWKGPAKLLWKGEGAVVIQDNNDIKVVPRRKAKIIRDYGKQMAGDDCVASRQDED
INa11_203_211               ---IIATDIQTK-----------------------------------------------------------------------------
hINb40_921_928              ------TDIQTKEL---------------------------------------------------------------------------
hINb40_911_919              IVDII------------------------------------------------------------------------------------
hINb40_912_920              IVDIIA-----------------------------------------------------------------------------------
hINcd4_rychert266           IVDIIATDIQTK-----------------------------------------------------------------------------
hINcd4_rychert266m          IVDIIATDIQTK-----------------------------------------------------------------------------
hINcd4_rychert273           ------------------------------------------LWKGEGAVVIQDNSDIKVVPRRKAKI---------------------
mINdr_247_261Walk           -----------------------------------------------GAVVIQDNSDIKVVP---------------------------
hINa2imus_241_249Reche      -----------------------------------------LLWKGEGAV---------------------------------------
hINb_187_204                IVDII------------------------------------------------------------------------------------
hINb_x35                    IVDIIATDIQTK-----------------------------------------------------------------------------
hINb_x36                    IVDIIATDIQT------------------------------------------------------------------------------
hINc14_Wang                 -----------KELQKQITK---------------------------------------------------------------------
hINb_242_259                ------------------------------------------LWKGEGAVVIQDNSDIKV-----------------------------
hINb_250_267                --------------------------------------------------VIQDNSDIKVVPRRKAKI---------------------
INa30_219_227               -------------------KIQNFRVYY-------------------------------------------------------------
hINa30_                     -------------------KIQNFRVYY-------------------------------------------------------------
hINmhc2_214_228             --------------QKQITKIQNFRVYYR------------------------------------------------------------
hINdrb1_1362_1376Wil        --------------QKQITKIQNFRVYYR------------------------------------------------------------
mINcd4_924_943              ---------QTKELQKQITKIQNFRVYYR------------------------------------------------------------
IN_210_227Lubo              ----------TEKLQKQITKIQNFRVYY---------------------------------------------------------
hINb_215_227                ---------------KQITKIQNFRVYY-------------------------------------------------------------
mINcd8_934_953              -------------------KIQNFRVYYRDSRNPLWKGP--------------------------------------------------
hIN_218_235Lubo             ------------------TKIQNFRVYYRDSRDPLW-----------------------------------------------------
INcw18_165_172              ---------------------------VRDQAEHL------------------------------------------------------
hINa2pre_225                -------------------------VYYRDSRDP-------------------------------------------------------
hINa2pre_226                --------------------------YYRDSRDPI------------------------------------------------------
INb72_263_271               ---------------------------------------------------------------RKAKIIRDY-----------------
INb42_260_268               ------------------------------------------------------------VPRRKAKII--------------------
Prim.cons.                  IVDIIATDIQTKELQKQIIKIQNFRVYYRDSRDPIWKGPAKLLWKGEGAVVIQDNNDIKVVPRRKAKIIRDYGKQMAGDDCVASRQDED
```

Supplementary Figure S1. Epitopic map of HIV-1 integrase. The alignment was performed basing on the information from epitope databases: Los Alamos HIV database (www.hiv.lanl.gov), Immune Epitope Database (http://www.immuneepitope.org), prediction of HLA-A*2 in integrase done by strength of binding and literature data (for links see Materials and methods section). Epitopes predicted from the strength of binding with HLA-A*2 are indicated in green. The boxes encompass the regions of IN with the maximum concentration of IN epitopes.

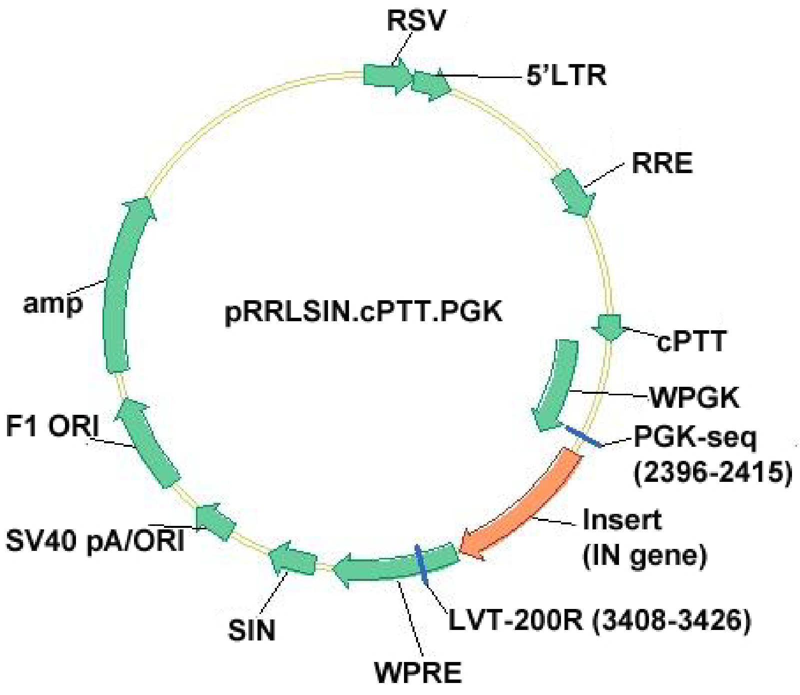

Supplementary Figure S2. The scheme of lentiviral vector used for cloning of IN variants. Position of the inserts of IN coding sequence is shown in orange. PGK-seq and LVT-200R primers (Supplementary Table S3) were used for PCR to confirm the insertion of IN coding sequences carried by lentiviral vectors into the genomic DNA of 4T1luc2 cells.

4T1luc2_IN_a_1.2 **4T1luc2_IN_a_r2_1.5**

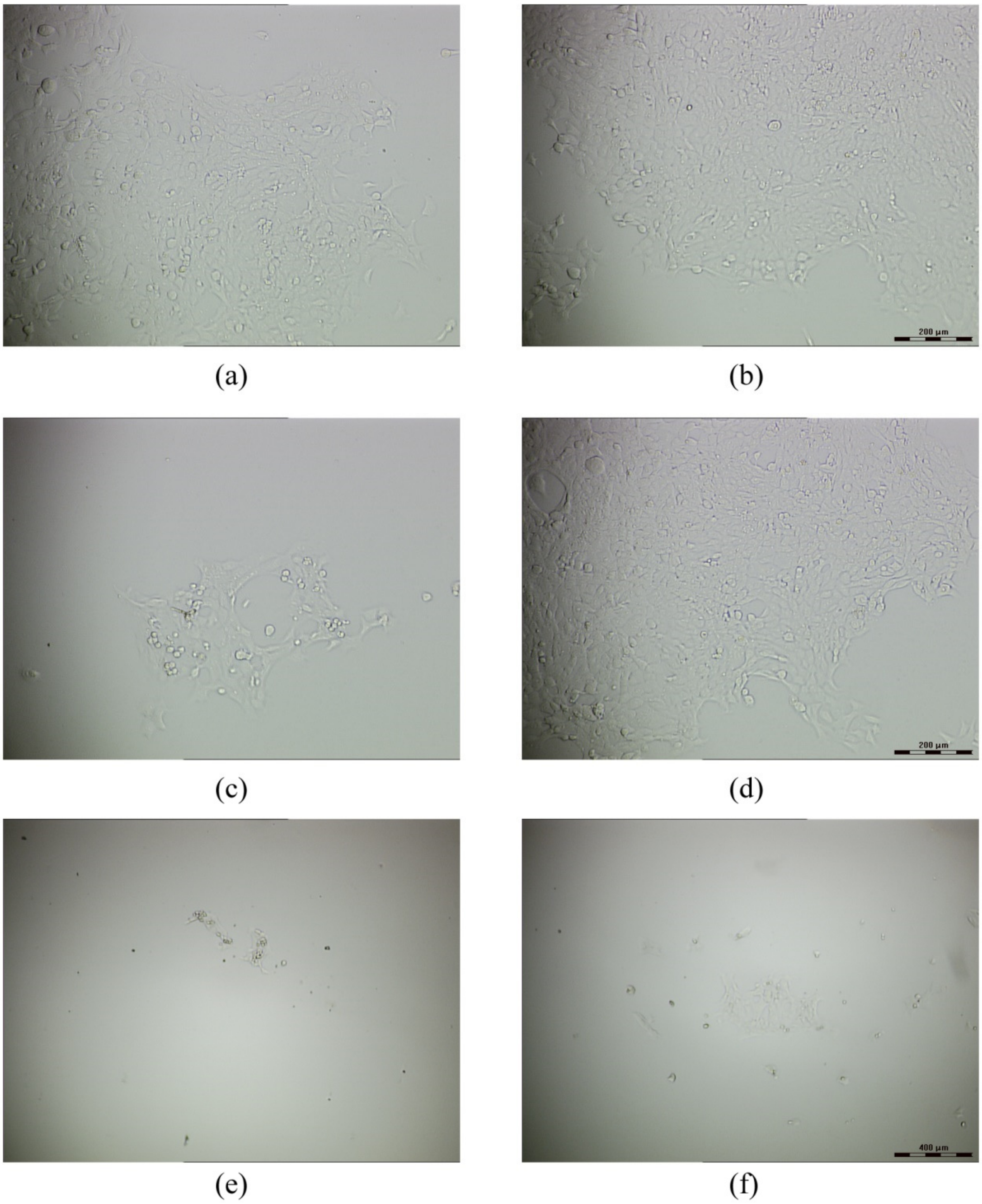

Supplementary Figure S3. 4T1luc2 cells after transduction with lentiviral particles encoding enzymatically active consensus HIV-1 clade A integrase. Transduction was done with lentiviral particles expressing enzymatically active parental integrase (IN_a) (a, c, e) or its variant with mutations of resistance to raltegravir E138K/G140S/Q148K (IN_a_r2) (b, d, f) with multiplicity of infection 1 (a, b), 5 (c, d), 20 (e, f).

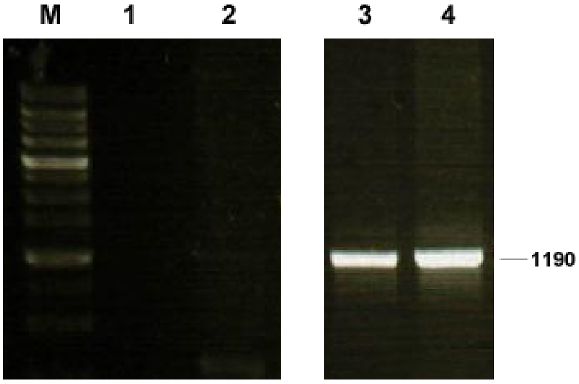

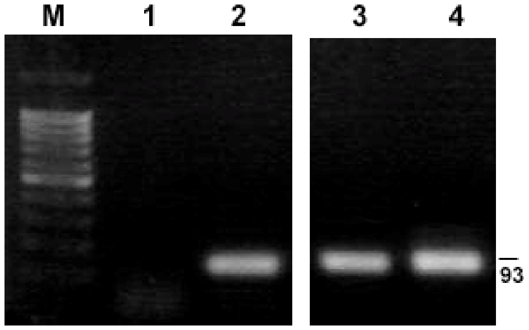

(a) (b)

Supplementary figure S4. Detection of the genomic inserts of sequences encoding HIV-1 clade A integrase under the control of PGK promoter in the daughter clones of 4T1luc2 cells. PCR of genomic DNA of 4T1luc2 cells transduced with lentiviral particles encoding enzymatically active consensus HIV-1 clade A integrase (IN_a; clone 4T1luc2_IN_a_1.2) or integrase with mutations of resistance to raltegravir E138K/G140S/Q148K (IN_a_r2; clone 4T1luc2_IN_a_r2_1.5). PCR was performed with specific primers PGKseq and LVN200 (a), and beta-actin primers for normalization (b). Control without template (lane 1); DNA of parental 4T1luc2 (lane 2), DNA of clone 4T1luc2_IN_a_1.2 (lane 3), and DNA of clone 4T1luc2_IN_a_r2_1.5 cells (lane 4), Kb molecular mass marker (lane M). Primer sequences are given in Supplementary Table S3. Figures on the right indicate the expected molecular mass of DNA corresponding to coding sequences of integrases (a) and actin (b).

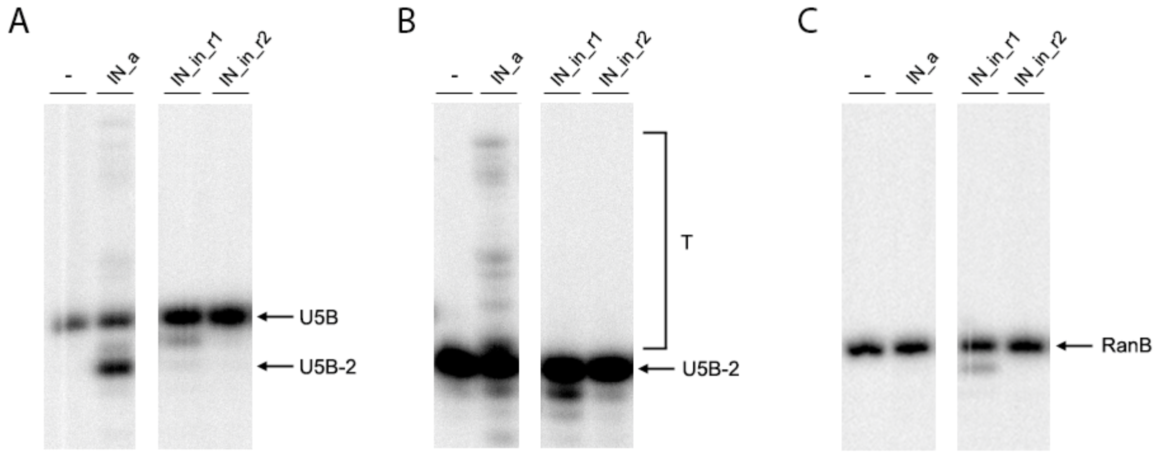

Supplementary Figure S5. Determination of the catalytic activity of consensus HIV-1 integrase (IN_a) and its variants with two patterns of mutations to raltegravir L74M/E92Q/V151I/N155H/G163R (IN_a_r1) and E138K/G140S/Q148K (IN_a_r2). Products of 3'-processing, strand transfer and non-specific exonuclease cleavage of the synthetic DNA duplexes with ^{32}P-labeled B-strands by the consensus HIV-1 clade A integrase (IN_a) and its inactivated variants with raltegravir resistance mutations (IN_in_r1, IN_in_r2) were separated by gel electrophoresis and quantified using Image-QuantTM 4.1 software. The 3'-processing assay: U5 substrate in the absence of integrases (-) and in the presence of IN_a, IN_in_r1 and IN_in_r2 (a); Strand transfer reaction: U5-2 substrate in the absence of integrases (-) and in the presence of IN_a, IN_in_r1, and IN_in_r2; T – the strand transfer products (b); Incubation of the non-specific DNA Ran in the absence of integrases (-), and in the presence of IN_a, IN_in_r1 and IN_in_r2 (c). Tests were performed with 100 nM integrases and 10 nM DNA. Products were separated in denaturing 20% PAAG with 7M urea (see Materials and Methods for details). Data are representative of two independent experiments. Oligonucleotide duplexes used to assess integrase activities are listed in the Supplementary Table S2.

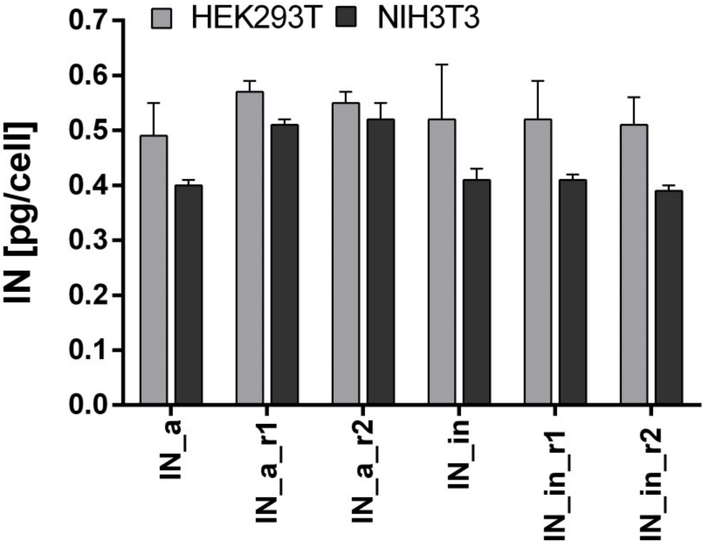

Supplementary Figure S6. Level of expression of variants of integrases of HIV-1 clade A in human (HEK293T) and murine (NIH3T3) cells, pg/cell. Plasmids based on pVax1 encoding enzymatically active integrase (IN_a), its variants with two patterns of mutations to raltegravir L74M/E92Q/V151I/N155H/G163R (IN_a_r1) and E138K/G140S/Q148K (IN_a_r2) and their inactivated analogies containing mutation D64V in the integrase active site, IN_in, IN_in_r1 and IN_in_r2, respectively, were transfected into HER293T or NIH3T3 cells. Expression levels were evaluated as described in the Materials and Methods. Data represent the results of two independent runs, each done in duplicate, mean± SD.

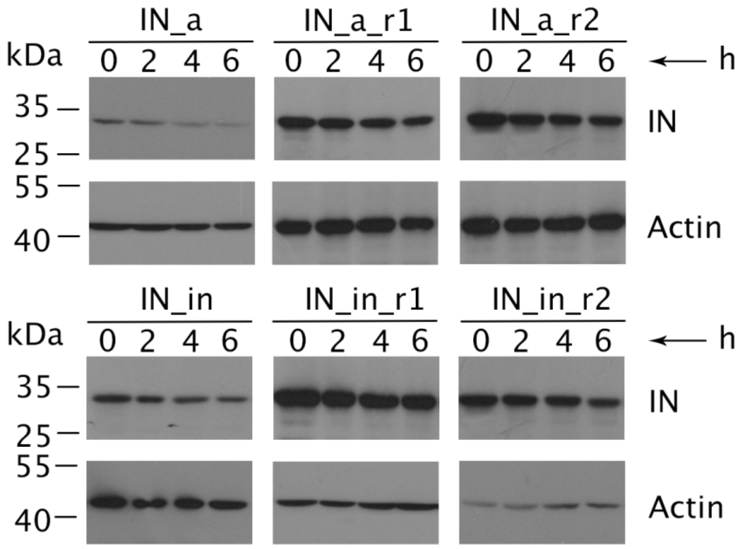

Supplementary Figure S7. The kinetics of integrase degradation determined by cycloheximide chase. HeLa cells were transiently transfected with pVax1-based plasmids expressing enzymatically active integrase (IN_a), its variants with two patterns of mutations to raltegravir L74M/E92Q/V151I/N155H/G163R (IN_a_r1) and E138K/G140S/Q148K (IN_a_r2) and their inactivated analogies containing mutation D64V in the integrase active site, IN_in, IN_in_r1 and IN_in_r2, respectively. At 48 h post transfection, transfected cells were treated with cycloheximide (CHI) at concentration 100 µg/ml. Cell lysates, prepared after 0, 2, 4 and 6 hours of incubation with CHI, were subjected to Western blot analysis using anti-IN antibody to detect expression of integrase (upper panels), and monoclonal anti-actin antibody for signal normalization (lower panels). Molecular mass markers as defined by the protein ladder (Page Ruler Prestained Protein Ladder, Fermentas) are given to the left.

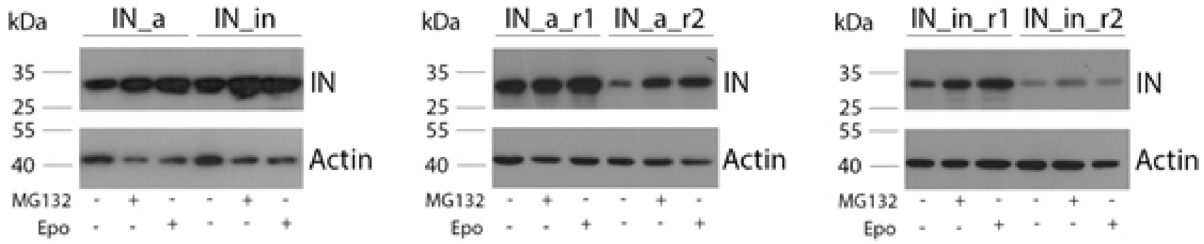

(a)

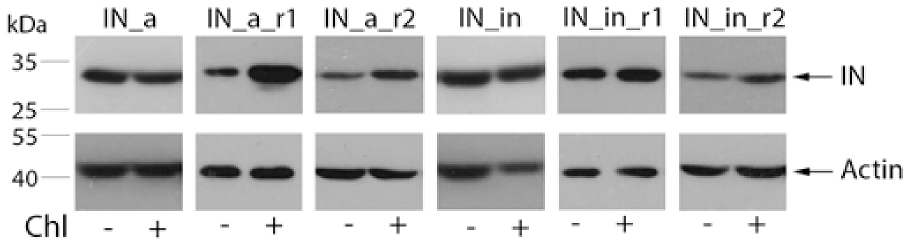

(b)

Supplementary Figure S8. Effect of the proteasome and lysosome inhibitors on accumulation of IN. HeLa cells were transiently transfected with pVax1-based plasmids expressing enzymatically active integrase (IN_a), its variants with two patterns of mutations to raltegravir L74M/E92Q/V151I/N155H/G163R (IN_a_r1) and E138K/G140S/Q148K (IN_a_r2) and their inactivated analogies containing mutation D64V in the integrase active site, IN_in, IN_in_r1 and IN_in_r2, respectively. At 30 hours post transfection transfected cells were treated with proteasomal inhibitors MG132 at 10 μM, or epoxomicin at 0.5 μM (a), or lysosomal inhibitor chloroquine (Chl) at 10 μM (b). Cell lysates prepared 18 hours after incubation with inhibitors were subjected to Western blot analysis with the mouse monoclonal anti-IN antibodies (upper panels), stripped, and the monoclonal anti-actin antibodies (lower panels). Lanes with treated samples are marked with "+", and lanes containing control untreated samples as "-". Molecular mass markers as defined by the protein ladder (Page Ruler Prestained Protein Ladder, Fermentas) are given to the left. Graphs representing the data of at least three independent experiments are presented in the main text of the article (Figure 3).

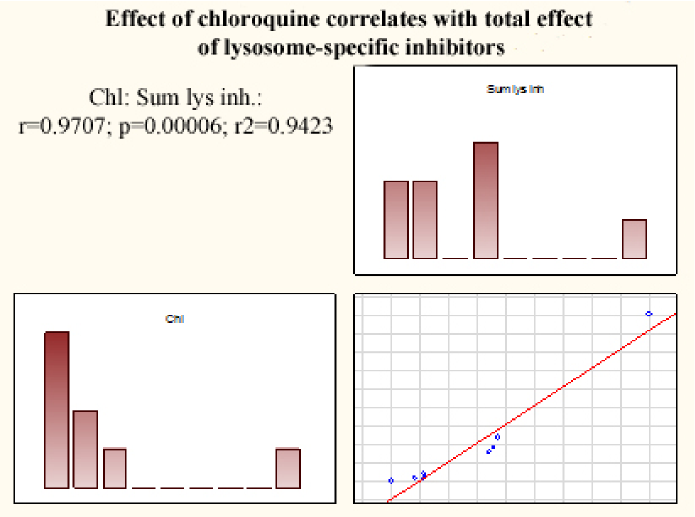

Supplementary Figure S9. The effect of a non-specific lysosomal inhibitor chloroquine is correlated to the effect of the combination of protease-specific inhibitors leupeptin, aprotinin, pepstatin and E-64. Data on the effects of chloroquine, leupeptin, aprotinin, pepstatin and E-64 inhibitors, and sum of their effects (Sum lys inh.) is presented in Figure 3c. Correlation analysis was performed using the Spearman rank test, $p<0.05$ was considered as significant.

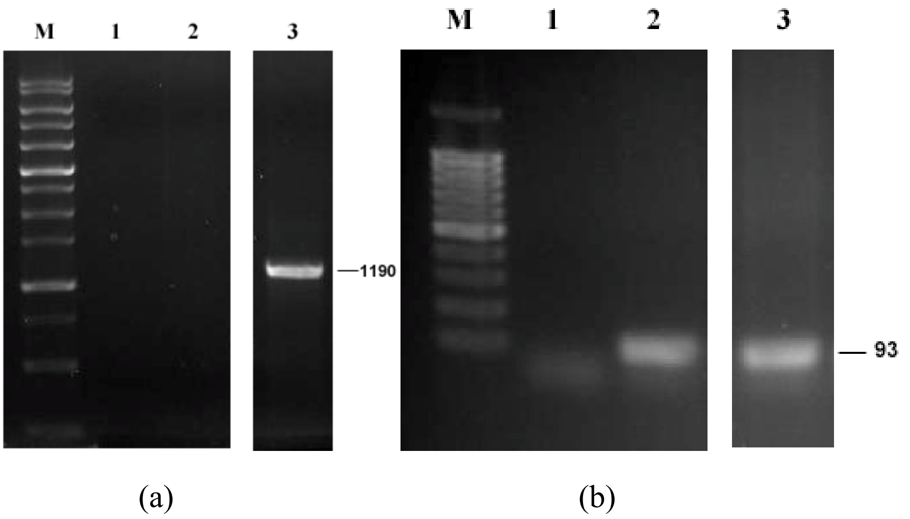

Supplementary figure S10. The results of PCR of genomic DNA of tumors produced by 4T1luc2 and their IN-expressing derivatives grown after ectopic implantation of BALB/c mice (the results for 4T1luc2_IN_a_r2_1.5 are shown). The PCR was performed with PGKseq and LVN200 primers (a) and beta-actin primers for normalization (b). The bands corresponding to 1 Kb marker (M), no template control (1) and cell lines 4T1luc2 (2), 4T1luc2_IN_a_r2_1.5 (3).

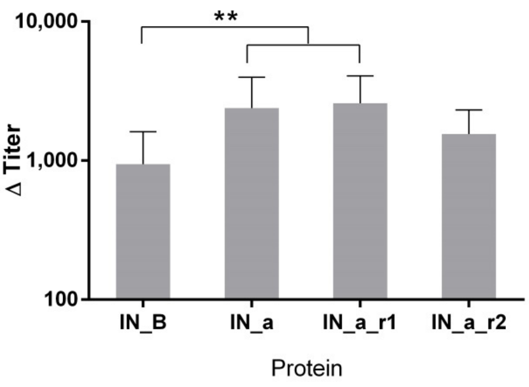
10,000
1,000
100
Δ Titer
**
IN_B
IN_a
IN_a_r1
IN_a_r2
Protein

(a)

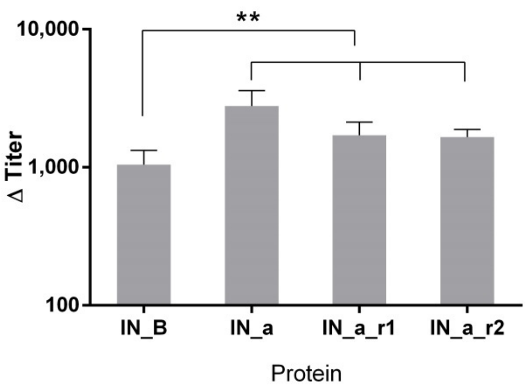
10,000
1,000
100
Δ Titer
**
IN_B
IN_a
IN_a_r1
IN_a_r2
Protein

(b)

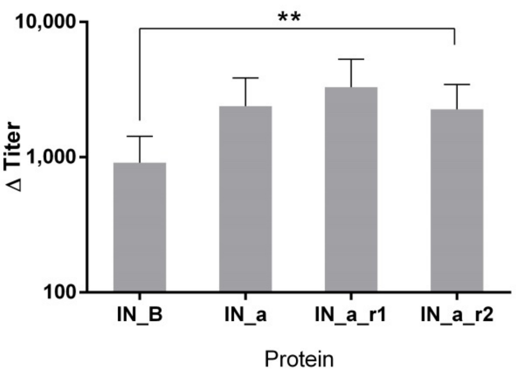
10,000
1,000
100
Δ Titer
**
IN_B
IN_a
IN_a_r1
IN_a_r2
Protein

(c)

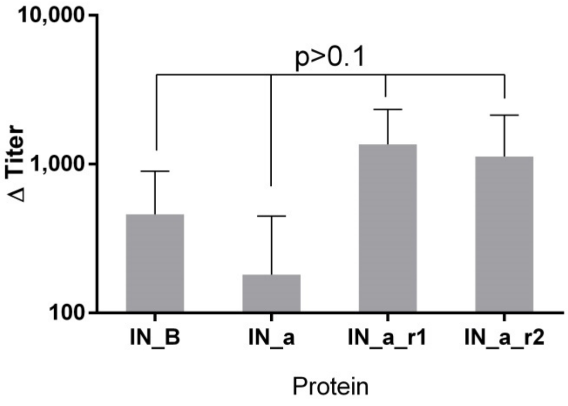
10,000
1,000
100
Δ Titer
p>0.1
IN_B
IN_a
IN_a_r1
IN_a_r2
Protein

(d)

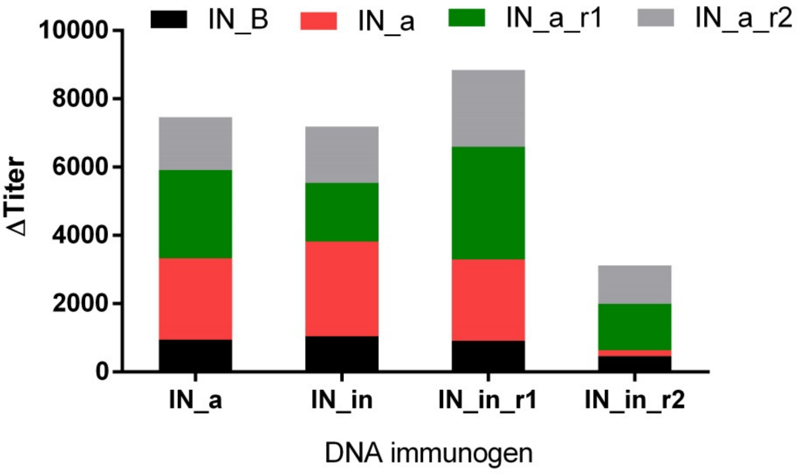
IN_B
IN_a
IN_a_r1
IN_a_r2
10000
8000
6000
4000
2000
0
ΔTiter
IN_a
IN_in
IN_in_r1
IN_in_r2
DNA immunogen

(e)

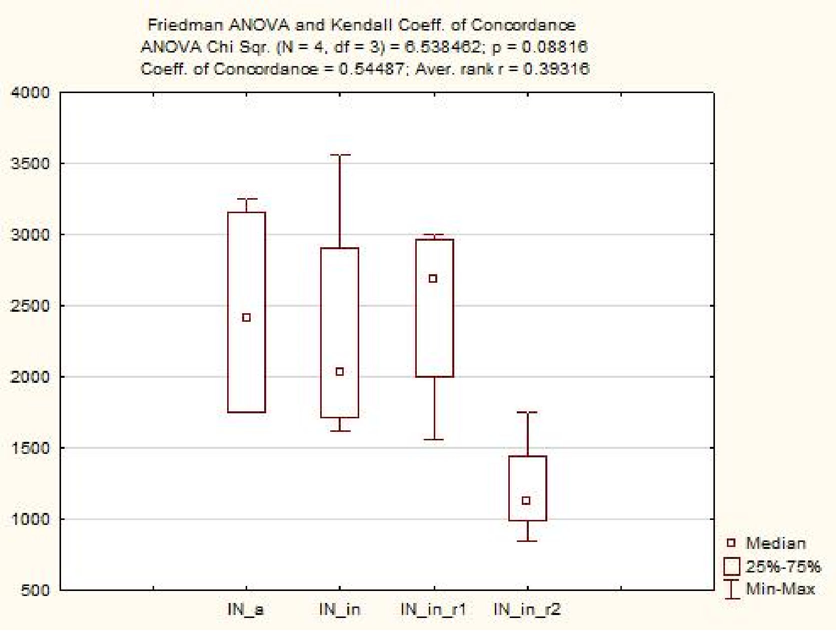

(f)

Supplementary Figure S11. DNA immunization with the IN gene variants induces cross-reactive IN-specific IgGs. End-point titers of the anti-IN IgG antibodies in the sera of BALB/c mice immunized with the genes encoding consensus HIV-1 integrase IN_a (a), Consensus IN inactivated by D64V mutation IN_in (b), and Consensus inactivated integrases carrying mutations conferring resistance to raltegravir IN_in_r1 (c) and IN_in_r2 (d); Pile up of antibody response against IN serving as immunogen and homologous IN protein variants (e); Statistical comparison of pile-up of antibody responses in mice DNA immunized with IN variants by Friedman ANOVA tests with Kendall coefficient of concordance (p=0,088) (f). Data represent the mean of two independent ELISA runs, each done in duplicate. Values on Y-axis (Δ Titer bar) represent the mean end-point antibody titers of individual mice DNA immunized with IN variants with subtracted mean end-point antibody titer of the vector group, ±SD. ** - p<0.01 (Mann-Whitney test); all the p-values shown passed the Holm multiple comparisons correction.

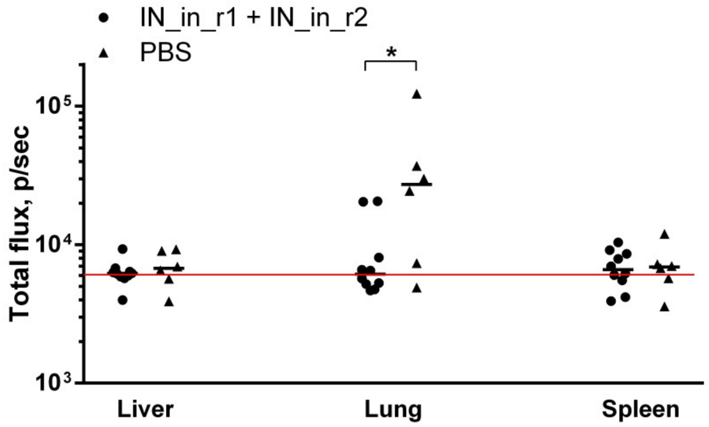

Supplementary Figure S12. Infiltration of tumor cells into organs of mice DNA immunized with IN variants challenged with 4T1luc2 cells derivatives expressing IN (4T1luc2_IN_a_1.2 and 4T1luc2_IN_a_r2_1.5) compared to mock immunized mice challenged with the same cell lines. Assessment of organ infiltrating tumor cells by *ex vivo* BLI of the liver, lungs and spleen; line bars represent the median of total flux from organs of mice DNA immunized with IN (n=10), or PBS (n=6) and implanted with IN-expressing 4T1luc2 cells; the red line intercepts the background bioluminescence characteristic of one cell emitting photons/sec; * - $p<0.05$, Kruskal-Wallis, Mann-Whitney tests.

Supplementary Table S1. Peptides and peptide pools used in *in vitro* T-cell stimulation tests. Choice of peptides was done based on the epitopic map (Supplementary Figure S1). IN series include peptides reported to be recognized by human T cells, and MIN series, by T cells of H2-K^d restricted BALB/c mice. Sources of the information on the epitopes are listed in Materials and Methods section. Peptides in the grey-shadowed zone were not recognized by the splenocytes of IN DNA immunized mice in the preliminary tests (data not shown) and were omitted from further analysis.

**Abbreviated name**			**Amino acid sequence**	**DR**
IN169		169-196	AEHLKTAVQMAVFIHNFKRKGGIGGYSA	N/A
Pool_MIN79	MIN79	79-98	VASGYIEAEVIPAETGQETA	N/A
	MIN79r1	79-98	VASGYIEAEVIPAQTGQETA	E92Q
MIN169		169-190	AEHLKTAVQMAVFIHNFKRKGG	N/A
MIN209		209-228	QTKELQKQIIKIQNFRVYYR	N/A
IN209		209-239	QTKELQKQIIKIQNFRVYYRDSRDPIWKGPA	N/A
MIN219		219-238	KIQNFRVYYRDSRDPIWKGP	N/A
MIN pool	MIN79	79-98	VASGYIEAEVIPAETGQETA	
	MIN79r1	79-98	VASGYIEAEVIPAQTGQETA	
	MIN169	169-190	AEHLKTAVQMAVFIHNFKRKGG	
	MIN209	209-228	QTKELQKQIIKIQNFRVYYR	
	MIN219	219-238	KIQNFRVYYRDSRDPIWKGP	
IN8	8-33	AQEEHEKYH	N/A	
IN36	36-50	IVASCDKCQL	N/A	
Pool_IN137	IN137	137-161	QEFGIPYNPQSQGVVESMNKELKKI	N/A
	IN137r1	137-161	QEFGIPYNPQSQGVIESMHKELKKI	V151I, N155H
	IN137r2	137-161	QKFSIPYNPQSKGVVESMNKELKKI	E138K, G140S, Q148K
	IN187	187-213	KRKGGIGGYSAGERIIDIIATDIQTKEL	N/A
	IN242	242-272	LWKGEGAVVIQDNNDIKVVPRRKAKIIRDY G	N/A

Supplementary Table S2. Synthetic oligonucleotide duplexes used to assess enzymatic activities of HIV-1 integrase.

Abbreviated name	Sequence	Function
**U5**	**U5A/U5B duplex**	**Specific integrase substrate in 3'-processing**
U5B	5'-GTGTGGAAAATCTCTAGCAGT-3'*	Strand processed by integrase
U5A	3'-CACACCTTTTAGAGATCGTCA-5'	Complementary to U5B
**U5-2**	**U5A/U5B-2 duplex**	**Integrase substrate in the strand transfer reaction**
U5A	3'-CACACCTTTTAGAGATCGTCA-5'	Complementary to U5B
U5B-2	5'-GTGTGGAAAATCTCTAGCA-3'	Result of U5B processing
**Ran**	**RanB/RanA duplex**	**Random non-specific DNA duplex**
RanB	5'-GGAATCTAGCGGCGCATAGGT-3'	Complementary to RanA
RanA	3'-CCTTAGATCGCCGCGTATCCA-5'	Complementary to RanB

**The dinucleotide removed by IN is underlined*

Supplementary Table S3. Primers used for generation of lentiviral vectors and confirmation of genomic IN inserts. Synthetic oligodeoxyribonucleotides were purchased from commercial companies (Evrogen, Russia).

**Purpose**	**Primer (probe) name**	**Target gene**	**Sequence**
Determination of infectious titers of lentiviral particles	GAG-F	Lentiviral gag	5’-GGAGCTAGAACGATTCGCAGTTA-3’
	GAG-R	Lentiviral gag	5’-GGTTGTAGCTGTCCCAGTATTTGTC-3’
	GAG-P	Lentiviral gag	5'-(FAM)-ACAGCCTTCTGATGTTTCTAACAGGCCAGG-(BHQ1)-3’
Determination of infectious titers of lentiviral particles	HB2-F	β-actin (human)	5’-TCCGTGTGGATCGGCGGCTCCA-3’
	HB2-R	β-actin (human)	5’-CTGCTTGCTGATCCACATCTG-3’
	HB2-P	β-actin (human)	5’-(HEX)-CCTGGCCTCGCTGTCCACCTTCCA-(BHQ2)-3’)
Confirmation of the insert of IN genes into lentiviral vector; assessment of presence of IN genes in DNA extracted from mouse tumors	PGKseq	Insert in lentiviral vector	5`-GGTGTTCCGCATTCTGCAAG-3’
	LVT-200R	Insert in lentiviral vector	5`-GACAACGGGCCACAACTCC-3’
Assessment of mRNA expression in mouse tumors	IN-dir	IN_a	5`-CTGGAAGGCAAGGTCATCAT-3`
	IN-rev	IN_a	5`-GCCAGTTTGAGCAGGAAGTA-3’
	GAPDH-D1	GAPDH	5’-GCATCCTGCACCACCAACTG-3’
	GAPDH-R1	GAPDH	5`-GAGCTTCCCGTTCAGCTCTG-3`

Supplementary Table S4. Comparison of *in vitro* and *in vivo* properties of IN-based DNA immunogens.

Table S4-1. Cummulative in vitro properties of IN DNA immunogens

Friedman ANOVA test with Kendall coefficeint of concordance.
ANOVA Chi Sqr. (N = 43, df = 3) = 6.449275; p=0.09169;
Coeff. of Concordance=0.04999; Aver. rank r=0.02738

	Average (Rank)	Sum of (Ranks)	Mean	Std.Dev.
IN_a	2.418605	104.0000	16.60954	27.16626
IN_in	2.581395	111.0000	16.09050	26.46058
IN_in_r1	2.837209	122.0000	16.77579	27.33850
IN_in_r2	2.162791	93.0000	16.59430	28.95471

Sign test

	No. of (Non-ties)	Percent (v < V)	Z	p-value
IN_a & IN_in	5	80.00000	0.894427	0.371093
IN_a & IN_in_r1	5	60.00000	0.000000	1.000000
IN_a & IN_in_r2	5	40.00000	0.000000	1.000000
IN_in & IN_in_r1	5	40.00000	0.000000	1.000000
IN_in & IN_in_r2	5	20.00000	0.894427	0.371093
IN_in_r1 & IN_in_r2	5	20.00000	0.894427	0.371093

Table S4-2. Cummulative in vivo properties of IN DNA-immunogens (T cell & Ab)

Friedman ANOVA test with Kendall coefficeint of concordance.
ANOVA Chi Sqr. (N = 38, df = 3) = 6.164835; p=0.1038
Coeff. of Concordance =0.05408 Aver. rank r=0.02851

	Average (Rank)	Sum of (Ranks)	Mean	Std.Dev.
IN_a	2.478261	114.0000	186.7220	670.9757
IN_in	2.630435	121.0000	168.5737	624.6345
IN_in_r1	2.804348	129.0000	167.8556	598.7066
IN_in_r2	2.086957	96.0000	82.9034	257.5373

Sign test

	No. of (Non-ties)	Percent (v < V)	Z	p-value
IN_a & IN_in	38	50.00000	-0.162221	0.871132
IN_a & IN_in_r1	38	57.89474	0.811107	0.417304
IN_a & IN_in_r2	38	36.84211	1.459993	0.144292
IN_in & IN_in_r1	34	58.82353	0.857493	0.391173
IN_in & IN_in_r2	34	38.23529	1.200490	0.229949
IN_in_r1 & IN_in_r2	34	32.35294	1.886484	0.059230

Table S4-3. Cummulative in vitro & in vivo properties of IN DNA-immunogens

Friedman ANOVA test with Kendall coefficeint of concordance.

ANOVA Chi Sqr. (N = 46, df = 3) = 8.027027; p=0.04546

Coeff. of Concordance = .05817 Aver. rank r = .03724

	Average (Rank)	Sum of (Ranks)	Mean	Std.Dev.
IN_a	2.478261	114.0000	186.7220	670.9757
IN_in	2.630435	121.0000	168.5737	624.6345
IN_in_r1	2.804348	129.0000	167.8556	598.7066
IN_in_r2	2.086957	96.0000	82.9034	257.5373

Sign test

	No. of (Non-ties)	Percent (v < V)	Z	p-value
IN_a & IN_in	46	54.34783	0.442326	0.658253
IN_a & IN_in_r1	46	58.69565	1.032094	0.302028
IN_a & IN_in_r2	46	39.13043	1.326978	0.184516
IN_in & IN_in_r1	42	54.76190	0.462910	0.643429
IN_in & IN_in_r2	42	35.71429	1.697337	0.089633
IN_in_r1 & IN_in_r2	42	30.95238	2.314550	0.020638

Supplementary Table S5. Correlations of the proportions of IFN-γ/IL-2 and IFN-γ/TNF-α secreting CD4+ and CD8+ T cells specific to integrase (MIN pool) in mice DNA-immunized with IN gene variants. Data from immunization Series I (Table 1). All MIN-specific T cell responses by individual mice in each group were summed, taken for 100% and response of each type in individual animals was presented as % of the total. Table depicts correlation coefficients in the Spearman rank order correlation test. Coefficients for statistically significant correlations ($p<0.05$) are given in red.

**Parameter**	**% $CD4^+$ IFN-γ/IL-2**	**% $CD4^+$ IFN-γ/TNF-α**	**% $CD8^+$ IFN-γ/IL-2**	**% $CD8^+$ IFN-γ/TNF-α**
% CD4+ IFN-γ/IL-2	1.000000	-0.508824	0.420588	-0.367647
% CD4+ IFN-γ/TNF-α	-0.508824	1.000000	-0.482353	0.541176
% CD8+ IFN-γ/IL-2	0.420588	-0.482353	1.000000	-0.797059
% CD8+ IFN-γ/TNF-α	-0.367647	0.541176	-0.797059	1.000000

Supplementary Table S6. Expression of IN variants in tumors formed by implantation of 4Tlluc2_IN_a_1.2 and 4T1luc2_IN_a_r2_1.5 cells into BALB/c mice. Total RNA was isolated from tumors formed in BALB/c mice after implantation with 4Tlluc2_IN_a_1.2 and 4T1luc2_IN_a_r2_1.5 cells and parental 4T1luc2 cells and transcription of IN genes was assessed by OneTube SYBR-RT PCR kit (Evrogen) using primers specific to IN gene, and murine GAPDH gene for normalization (Supplementary Table S3). Cycle threshold (Ct) values ± SD were determined from amplification curves and dCt was calculated. IN expression was detected in tumors formed by 4T1luc2_IN_a_1.2 and 4T1luc2_IN_a_r2_1.5 and not in tumors formed by 4T1luc2 cells. The experiment was performed in duplicates.

	**Ct GAPDH ± SD**	**Ct IN ± SD**	**dCt**
4T1luc2	34.12±0.46	N/A	N/A
4Tlluc2_IN_a_1.2	27.50±0.25	28.13±0.12	-0.63
4T1luc2_IN_a_r2_1.5	32.36±0.21	34.23±0.05	-1.87

Supplementary Table S7. Animal welfare chart for the parameters assessed daily and weekly. Mice DNA immunized with plasmid encoding inactive integrase IN_in, or a mixture of plasmids encoding RAL resistant inactivated integrases IN_in_r1 and IN_in_r2 (1:1, v/v), or empty vector pVax1, or PBS, followed by electroporation, were assessed by veterinary doctor for general health condition on daily and weekly basis. The general health condition was assessed daily according to the chart «Parameters assessed daily» without scoring. An in-depth clinical examination with scoring was performed weekly according to the chart «Parameters assessed weekly». General health condition was described by marking checkboxes corresponding to normal or altered condition; altered conditions were to be specified in the "Comments" section. The condition of all animals at all timepoints was marked as normal with no differences observed between study groups.

Parameters		Mark if yes	Comments
**Parameters assessed daily**			
Behavior	Normal	◻	
	Altered (depression/agitation)	◻	
Reaction to stimuli	Normal	◻	
	Altered (decrease/increase)	◻	
Skin	Normal	◻	
	Altered (redness/pallor/cyanosis/icteric)	◻	
Mucous membranes	Normal	◻	
	Altered (redness/pallor/cyanosis/icteric)	◻	
Discharge (nasal/eyes/anal/uretral)	Normal	◻	
	Altered	◻	
Muscle tone	Normal	◻	
	Altered (decrease/increase)	◻	
Coordination	Normal	◻	
	Altered (ataxia/hyperkinesis)	◻	
Breathing	Normal	◻	
	Altered (dyspnea/pathologic)	◻	
Feed and water consumption	Normal	◻	
	Altered (decrease/increase)	◻	
Other	Describe	◻	
Death		◻	
**Parameters assessed weekly in cage**			
Behavior	Normal	◻	
	Altered (depression/agitation)	◻	
Social interactions	Normal	◻	
	Altered (aggression)	◻	
**Parameters assessed weekly in hands**			
Reaction to stimuli	Normal	◻	
	Altered (decrease/increase)	◻	
Body composition	Normal	◻	

	Altered (wasting/obesity)	◻	
Muscle tone	Normal	◻	
	Altered (decrease/increase)	◻	
Fur	Normal	◻	
	Altered (disheveled/shedding/dull/dirty/discolored)	◻	
Skin	Normal	◻	
	Altered (describe: turgor, color, wounds, palpable mass)	◻	
Mucous membranes	Normal	◻	
	Altered (redness/pallor/cyanosis/icteric)	◻	
Eyes	Normal	◻	
	Altered (exophthalmos/wounds/redness/discharge)	◻	
Ears	Normal	◻	
	Altered (inflammation/discharge)	◻	
Nose	Normal	◻	
	Altered (serous discharge/purulent discharge/bloody discharge)	◻	
Mouth	Normal	◻	
	Altered (hypersalivation, blood, damaged teeth)	◻	
**Parameters assessed weekly in open field**			
Pose	Normal	◻	
	Altered (stereotypic movements, laying on side)	◻	
Coordination	Normal	◻	
	Altered (ataxia/hyperkinesis)	◻	
Breathing	Normal	◻	
	Altered (dyspnea/pathologic)	◻	
Urine	Normal	◻	
	Altered (discoloration)	◻	
Feces	Normal	◻	
	Altered (diarrhea/blood/discoloration)	◻	
Other	Describe	◻	

Supplementary Table S8. Body mass assessment in mice receiving plasmid encoding inactivated integrase (IN_in), a mixture of plasmids encoding drug resistant inactivated integrases IN_in_r1 and IN_in_r2 (1:1, v/v), empty vector pVax1, or PBS, followed by electroporation (Table 1).

Days of experiment	Body mass in study group, mean±SD, g			
	IN_in (n=5)	IN_in_r1 + IN_in_r2 (n=6)	Vector (n=5)	PBS (n=6)
0	24.6±1.6	23.6±2.0	23.8±1.3	24.3±1.2
1*	23.3±1.4	22.3±2.2	23.0±1.0	24.0±1.5
3	23.7±1.3	22.8±2.5	23.0±0.9	23.8±1.2
4	23.7±1.3	23.1±2.4	23.4±0.8	24.1±1.2
5	24.2±1.3	23.4±2.4	24.1±0.7	24.4±1.1
6	24.4±1.3	23.6±2.2	24.2±0.8	24.7±1.3
7	24.8±1.1	23.8±2.3	24.2±0.8	24.7±1.3
8	25.0±1.2	23.9±2.0	24.3±0.8	25.3±1.4
11	25.1±0.9	24.3±2.0	24.7±1.2	25.6±1.4
12	25.2±1.2	24.3±1.9	24.6±1.2	25.5±1.3
13	25.5±0.9	24.6±1.7	25.1±1.1	26.0±1.4
14	25.9±1.0	24.8±1.6	25.4±1.1	26.4±1.5
15	25.7±0.8	24.8±2.0	25.6±1.1	26.5±1.6
18	25.9±0.9	25.2±1.8	25.7±1.1	26.4±1.7
19	26.2±1.0	25.6±1.8	26.0±1.3	26.8±1.5
20	25.6±1.1	24.5±1.8	25.2±1.5	26.3±1.3
22**	25.5±0.9	24.3±1.6	25.0±1.4	26.0±1.2
25	25.6±1.1	24.6±1.3	24.8±2.0	26.5±1.5
26	25.5±0.9	24.6±1.5	24.8±2.3	26.8±1.5
27	25.8±1.3	24.9±1.7	24.9±2.7	27.0±1.4
28	26.2±1.0	25.1±1.6	24.9±2.3	27.3±1.3
29	25.9±1.2	24.8±1.5	24.9±2.4	26.6±1.2
36	25.3±1.3	24.4±0.9	24.3±2.4	26.4±1.1

** Day 1, priming immunization;*

*** Day 22, booster immunization.*

Supplementary Table S9. Complete blood counts of mice receiving plasmid encoding inactivated integrase (IN_in), a mixture of drug resistant inactivated integrases IN_in_r1 and IN_in_r2 (1:1, v/v), empty vector pVax1, or PBS, followed by electroporation (Table 1). Blood was collected on day 13 after booster immunization.

**Tested parameters***	Study groups, mean±SD			
	IN_in (n=5)	IN_in_r1 + IN_in_r2 (n=6)	Vector (n=5)	PBS (n=6)
**RBC (x10^12/L)**	10.18±0.47	9.93±0.53	10.1±0.32	9.86±0.3
**HCT (%)**	47.12±1.79	45.9±2.83	44.98±2.57	45.77±1.47
**PLT (x10^9/L)**	**1046.8±130.56** **	941.33±161.77	712.2±177.18	874.83±158.26
**HGB (g/L)**	150±6.28	147±6.03	144.2±8.04	147.67±5.5
**WBC (x10^9/L)**	**16.28±0.55** ***	11.2±3.43	11.9±1.62	9.68±3.13
**LYM (%)**	71.96±4.05	72.83±3.89	71.5±4.24	66.82±7.02
**MID (%)**	2.86±0.42	2.63±0.36	2.46±0.34	2.73±0.67
**GRAN (%)**	25.18±3.69	24.53±3.69	24.06±4.09	30.45±6.39

** Complete blood counts including red blood cells (RBC), hematocrit (HCT), platelets (PLT), hemoglobin (HGB), white blood cells (WBC), lymphocytes (LYM), unclassified leukocytes (MID) and granulocytes (GRAN). Two blood samples (one from group IN_in and one from pVax1 group) did not pass quality check procedure of the on the hematology analyzer and were excluded from analysis.*

*** $p<0.05$, statistically significant difference between mice DNA-immunized with IN_in and pVax1 Mann-Whitney tests with Bonferroni correction.*

**** $p<0.05$, statistically significant difference between mice DNA-immunized with IN_in and IN_in_r1+IN_in_r2; IN_in and pVax1; and IN_in and PBS; Kruskal-Wallis and Mann-Whitney tests with Bonferroni correction.*

Supplementary Table S10. Biochemical blood analysis of mice receiving plasmid encoding inactivated integrase (IN_in), a mixture of drug resistant inactivated integrases IN_in_r1 and IN_in_r2 (1:1, v/v), empty vector pVax1, or PBS, followed by electroporation (Table 1). Blood was collected on day 13 after booster immunization.

**Tested parameters***	Study groups, mean±SD			
	IN_in (n=5)	IN_in_r1 + IN_in_r2 (n=6)	Vector (n=5)	PBS (n=6)
**ALT**	26.88±7.69	31.74±10.54	46.2±13.06	41.7±14.7
**AST**	228.6±88.54	203.88±77.51	324.54±115.57	290.28±71.14
**ALP**	427±29.86	394.4±61.02	367±144.66	402.8±112.88
**LDH**	2298.4±206.4	2270.8±588.97	2931.7±416.91	2726.4±460.58
**TP**	**64±4.26** **	54.02±2.15	56.54±7.76	63±12.81
**ALB**	**45.16±1.23** **	39.28±1.39	44.44±7.71	42.04±7.06
**UREA**	8.99±1.34	6.81±1.38	8.93±2.57	8.93±2.57
**GLU**	5.34±1.66	7.04±1.53	7.08±3.17	6.85±1.37
**CHOL**	2.24±0.44	1.94±0.51	2.16±0.56	2.13±0.31
**TGC**	**1.43±0.12** **	1.06±0.07	1.45±0.28	1.26±0.24
**Na**	126.82±5.74	125.72±5.61	121.78±6.03	124.1±3.94
**K**	9.72±1.94	8.44±1.81	7.54±2.32	8.16±1.18

** Abbreviations: alanine aminotransferase, ALT; aspartate aminotransferase, AST; alkaline phosphatase, ALP; lactate dehydrogenase, LDH; total protein, TP; serum albumin, ALB; glucose, GLU; cholesterol, CHOL; triglycerides, TGC; blood electrolytes Na+, K+ ions, Na, K.*

*** Statistically significant difference in Kruskal-Wallis and Mann-Whitney tests with Bonferroni correction. Bold font represents statistically significant ($p<0.05$) values in Mann-Whitney test.*

Supplementary Table S11. Bone marrow composition of mice receiving plasmid encoding inactivated integrase (IN_in), a mixture of drug resistant inactivated integrases IN_in_r1 and IN_in_r2 (1:1, v/v), empty vector pVax1, or PBS, followed by electroporation (Table 1). Samples were collected on day 13 after booster immunization. Statistical analysis was done using Kruskal-Wallis and Mann-Whitney tests with Bonferroni correction. No statistically significant differences were observed between study groups.

Tested parameters*	Study groups, mean±SD			
	IN_in (n=5)	IN_in_r1 + IN_in_r2 (n=6)	Vector (n=5)	PBS (n=6)
**Erythroblasts**	0.4±0.55	0.33±0.52	0.2±0.45	0.33±0.52
**Proerythroblasts**	0.6±0.55	0.5±0.55	0.4±0.55	0.5±0.55
**Basophylic erythroblasts**	4.2±0.84	3.83±1.17	3.6±0.55	4±0.63
**Polychromatophilic erythroblast**	13.20±1.3	12.83±2.04	12.8±0.84	13±1.55
**Oxyphilic erythroblasts**	15±1.22	15.33±1.63	14.6±1.14	15±1.26
**Lymphocytes**	18.2±1.64	18.5±1.05	18.4±1.34	18.5±1.64
**Monocytes**	7±1.73	7.17±0.75	7.4±0.55	7.33±0.82
**Reticuloendothelial cells**	1.20±0.45	1.33±0.52	1.2±0.45	1.17±0.41
**Myeloblasts**	0.8±0.45	0.33±0.52	0.6±0.55	0.5±0.55
**Promyelocytes**	0.8±0.84	1.17±0.75	0.8±0.45	0.83±0.41
**Myelocytes**	4.6±1.34	4±0.63	3.8±0.84	4±0.89
**Metamyelocytes**	4.4±1.52	3.67±0.82	3.4±0.55	3.83±1.17
**Band neutrophils**	8.8±0.84	8.5±1.64	8.4±0.55	8.83±0.75
**Segmented neutrophils**	19±2.12	20±1.67	21.6±1.95	19.67±2.25
**Basophils**	0.6±0.55	0.67±0.52	0.4±0.55	0.5±0.55
**Eosinophils**	1±0.71	1.17±0.41	1.4±0.55	1.17±0.41
**Other cells**	0.6±0.55	0.67±0.52	0.8±0.45	0.83±0.41

Supplementary Table S12. Organ mass of mice receiving plasmid encoding inactivated integrase (IN_in), a mixture of drug resistant inactivated integrases IN_in_r1 and IN_in_r2 (1:1, v/v), empty vector pVax1, or PBS, followed by electroporation (Table 1). Mice were euthanized and organs were excised and weighed on day 13 after booster immunization. Statistical analysis was done using Kruskal-Wallis and Mann-Whitney tests with Bonferroni correction**.** No statistically significant differences were observed between study groups.

**Organ**	Study groups, mean±SD			
	IN_in (n=5)	IN_in_r1 + IN_in_r2 (n=6)	Vector (n=5)	PBS (n=6)
**Thymus**	0.11±0.05	0.11±0.02	0.12±0.02	0.1±0.04
**Spleen**	0.43±0.05	0.39±0.03	0.48±0.13	0.44±0.02
**Axillary lymph node**	0.02±0.01	0.02±0.01	0.02±0.01	0.02±0.01
**Inguinal lymph node**	0.03±0.01	0.02±0.02	0.04±0.01	0.12±0.25

Supplementary Table S13. Inverse correlation of IFN-γ/IL-2 secretion by splenocytes in response to stimulation with IN peptides to mean tumor volume (cm3) and total photon flux from tumor area (p/sec) of mice immunized with IN gene variants and challenged with tumorigenic cell lines stably expressing IN. R and p values are indicated in the table (Spearman rank correlation), * - $p<0.05$; ** - $p<0.0001$.

	**IFN-γ MIN219**	**IL-2 MIN219**	**IL-2 MIN79+MIN79r1**	**IFN-γ/IL-2 MIN219**	**IFN-γ/IL-2 MIN79+MIN79r1**
**Tumor volume**	R= -0.749 *	Non-significant	Non-significant	R= -0.67 *	Non-significant
**Total flux**	R= -0.933 **	R= -0.933 **	R= -0.748 *	R= -0.95 **	R= -0.667 *
